# Context-Specific Effects of TGF-β/SMAD3 in Cancer Are Modulated by the Epigenome

**DOI:** 10.1016/j.celrep.2015.11.040

**Published:** 2015-12-10

**Authors:** Ana Tufegdzic Vidakovic, Oscar M. Rueda, Stephin J. Vervoort, Ankita Sati Batra, Mae Akilina Goldgraben, Santiago Uribe-Lewis, Wendy Greenwood, Paul J. Coffer, Alejandra Bruna, Carlos Caldas

**Affiliations:** 1 Cancer Research UK Cambridge Institute, Department of Oncology, University of Cambridge, Cambridge CB2 0RE, UK; 2 Department of Cell Biology, Center for Molecular Medicine, University Medical Center Utrecht, 3584 CX Utrecht, the Netherlands

## Abstract

The transforming growth factor beta (TGF-β) signaling pathway exerts opposing effects on cancer cells, acting as either a tumor promoter or a tumor suppressor. Here, we show that these opposing effects are a result of the synergy between SMAD3, a downstream effector of TGF-β signaling, and the distinct epigenomes of breast-tumor-initiating cells (BTICs). These effects of TGF-β are associated with distinct gene expression programs, but genomic SMAD3 binding patterns are highly similar in the BTIC-promoting and BTIC-suppressing contexts. Our data show cell-type-specific patterns of DNA and histone modifications provide a modulatory layer by determining accessibility of genes to regulation by TGF-β/SMAD3. *LBH*, one such context-specific target gene, is regulated according to its DNA methylation status and is crucial for TGF-β-dependent promotion of BTICs. Overall, these results reveal that the epigenome plays a central and previously overlooked role in shaping the context-specific effects of TGF-β in cancer.

## Highlights

• TGF-β has opposing effects in different breast-tumor-initiating cell (BTIC) types
• Genomic SMAD3 binding patterns are similar in BTICs with opposing responses to TGF-β
• BTIC type-specific epigenomes prime genes for regulation by TGF-β/SMAD3
• *LBH*, a type-specific TGF-β target, is essential for BTIC-promoting effects of TGF-β

## Graphical Abstract

**Figure:**
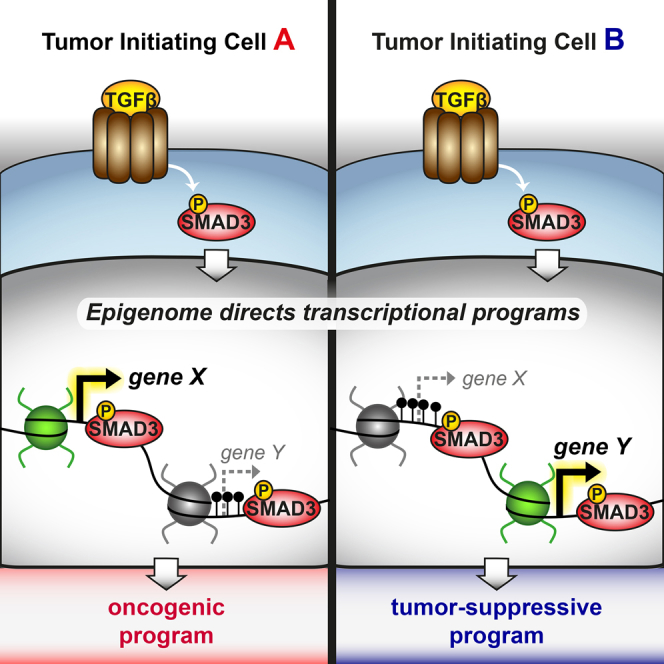

## Teaser

The TGF-β pathway uses transcriptional regulation through SMAD transcription factors to modulate cell-context-specific phenotypes. Tufegdzic Vidakovic et al. show that in breast-tumor-initiating cells (BTICs), type-specific DNA and histone modifications help determine whether the response to TGF-β is pro-oncogenic or tumor suppressive. These landscapes act both in synergy and independently of cell-type-specific SMAD3 binding to TGF-β target genes to modulate context-specific transcriptional regulation by TGF-β/SMAD3.

## Introduction

The effects of transforming growth factor beta (TGF-β) in tissue homeostasis depend heavily on cellular context (Massagué, 2012). TGF-β has been shown to both induce proliferation and suppress cell growth, stimulate stem cell self-renewal and promote differentiation, and inhibit early and promote late malignant transformation (Gomis et al., 2006, Guasch et al., 2007, Massagué, 2008, Massagué, 2012).

In breast cancer, TGF-β can either promote or inhibit tumor-initiating cells (breast TICs, or BTICs), which are responsible for cancer initiation, propagation, and metastasis (Bierie and Moses, 2009, Bruna et al., 2012, Mani et al., 2008, Scheel et al., 2011). We have previously shown these opposing effects of TGF-β depend on breast cancer subtype (Bruna et al., 2012). BTICs are activated only in Claudin^low^ breast cancer, while in all other subtypes, TGF-β inhibits BTICs. Since no mutations in TGF-β pathway genes have been associated with specific breast cancer subtypes (Cancer Genome Atlas Network, 2012), the underlying mechanism of this dichotomy is unlikely to be genetic.

TGF-β signaling is initiated by binding of TGF-β to its cognate receptor, TGFBR II, resulting in phosphorylation of the transcription factors SMAD2 and SMAD3 (Massagué et al., 2005). Upon phosphorylation, SMAD2 and SMAD3 associate with SMAD4 and translocate to the nucleus, where they partner up with additional transcription factors (TFs) to regulate target gene expression (Massagué et al., 2005). Remarkably, TGF-β universally relies on SMADs despite regulating cell-type-specific transcriptional programs (Massagué, 2012). The current model is that cell-type-specific partner TFs guide SMADs to distinct genes, thus resulting in context-specific gene regulation and specific biological effects of TGF-β (Massagué, 2012, Mullen et al., 2011, Xu et al., 2015).

Here, we mapped genome-wide SMAD3 binding patterns in BTICs that model the opposing effects of TGF-β (Bruna et al., 2012). This showed that differential SMAD3 binding does not fully account for context-specific TGF-β target gene regulation, and further experiments revealed that distinct epigenetic states are responsible. We identify transcription factor *LBH* as a prototypical TGF-β target gene regulated by differential DNA methylation and show it is essential for the BTIC-promoting activity of TGF-β. Taken together, these data reveal an important role for epigenetic determinants in regulation of the context-specific actions of TGF-β in cancer.

## Results

### SMAD3 Binding to Gene-Proximal Regions Mediates TGF-β-Dependent Gene Expression in BTICs

**Figure 1:**
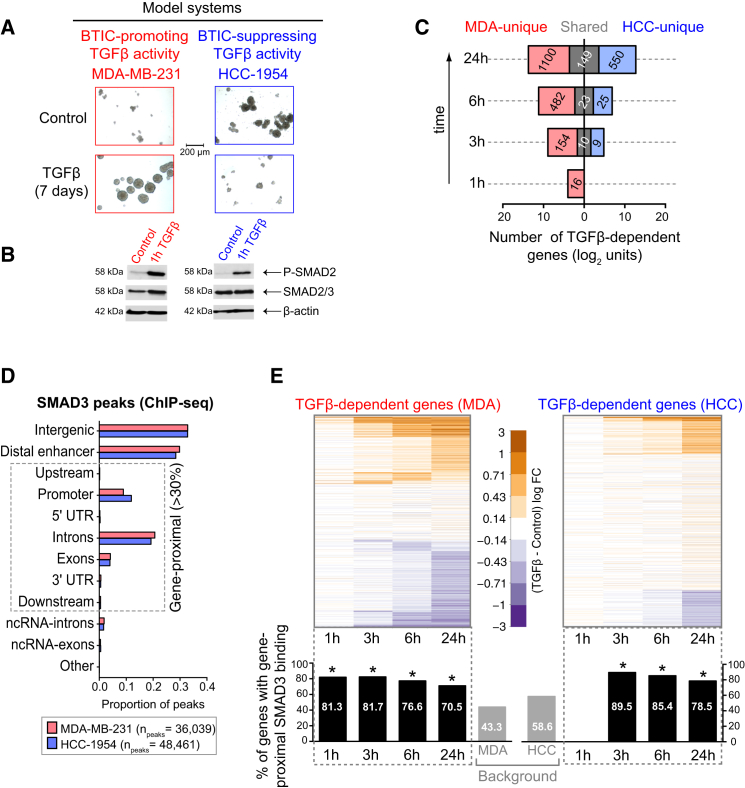
SMAD3 Mediates Both the BTIC-Promoting and BTIC-Suppressing Programs of TGF-β (A) MDA-MB-231 and HCC-1954 first generation mammosphere cultures with and without addition of TGF-β. TGF-β was added to the media at the moment of cell seeding and mammospheres were allowed to form for 7 days. Note that this is not a quantitative assay. (B) Western blot showing SMAD2 phosphorylation levels upon TGF-β pathway induction. 7-day-old mammospheres were treated with exogenous TGF-β ligand for 1 hr. Total SMAD2/3 and β-actin levels were used as loading controls. (C) Comparison of TGF-β-dependent genes in MDA-MB-231 and HCC-1954 BTICs. MDA-MB-231 and HCC-1954 cells were grown as mammospheres for 7 days and treated with TGF-β for 1, 3, 6, and 24 hr, and gene expression profiling was performed using Illumina HumanHT-12 BeadChips. The plot shows the number of TGF-β-dependent genes in each BTIC at each time point (false discovery rate [FDR] < 0.1; data presented on the log_2_ scale to show both the early and late response). TGF-β-dependent genes unique to MDA-MB-231 or HCC-1954 BTICs are labeled in red and blue, respectively, whereas genes regulated by TGF-β in both BTIC types are labeled in gray. The number of genes within each category is indicated within bars. Also see Table S1. (D) Annotation of SMAD3 binding sites in the genomes of MDA-MB-231 BTICs (red) and HCC-1954 BTICs (blue). ChIP-seq was performed on 7-day-old mammospheres treated with TGF-β for 3 hr. See Supplemental Experimental Procedures for details. (E) Heatmaps showing gene expression dynamics upon TGF-β stimulation in each BTIC independently. Bar plots below each gene expression time point show the proportion of TGF-β-dependent genes at that particular time point that were detected as bound by SMAD3 in the ChIP-seq experiment (bar below the 1-hr time point in HCC-1954 is absent as zero genes were detected as significantly differentially expressed). Asterisks indicate statistical significance, which was determined with the chi-square test, using TGF-β-independent gene sets as background (refer to Supplemental Experimental Procedures for details).

Two cell lines that we previously showed represent the opposing effects of TGF-β (Bruna et al., 2012) were used as BTIC model systems in all experiments: MDA-MB-231 for BTIC promoting, and HCC-1954 for BTIC suppressing (Figure 1A). Cells were grown in suspension as mammosphere cultures to enrich for BTICs (Bruna et al., 2012, Dontu et al., 2003a, Dontu et al., 2003b). Confirming our previous data (Bruna et al., 2012), the canonical TGF-β signaling cascade is intact and similarly activated by its ligand in both models, as shown by SMAD2 phosphorylation (Figure 1B).

The transcriptional responses associated with the opposing effects of TGF-β on BTICs were characterized by gene expression profiling. BTIC-enriched mammosphere cultures (hereafter referred to as “BTICs”) were treated with TGF-β for varying amounts of time (1, 3, 6, and 24 hr) to capture both early and late transcriptional responses. Comparing the lists of TGF-β-dependent genes revealed that only a small fraction is commonly regulated in both BTIC types (“shared” genes) (Figure 1C; Table S1). The vast majority of genes displayed cell-context-specific regulation, indicating that distinct and non-overlapping TGF-β-dependent transcriptional regulation occurs in BTICs with opposing (pro-oncogenic and tumor-suppressive) responses.

We previously showed that the BTIC-promoting and BTIC-suppressing effects of TGF-β depend on SMADs (Bruna et al., 2012). Hence, we hypothesized that SMADs mediate the TGF-β-dependent transcriptional regulation in both contexts. SMAD3 binding patterns in BTICs were mapped genome-wide after 3 hr of TGF-β exposure using chromatin immunoprecipitation and sequencing (ChIP-seq). We chose the 3-hr time point as it was the earliest at which significant TGF-β-dependent gene expression changes were detected in both models. Genomic annotation of SMAD3 binding sites showed a significant fraction of peaks (>30%) is directly associated with genes, with the remainder being at distal regulatory regions (Figure 1D).

We defined gene-proximal SMAD3 binding when peaks occurred within a genomic unit encompassing the 1,500 bp upstream of the transcription start site (TSS) to the end of the gene body. Comparing TGF-β-dependent and TGF-β-independent (background) gene sets revealed that both early- and late-responder genes are strongly enriched for gene-proximal SMAD3 binding in both BTIC types (Figure 1E). These data suggest that gene-proximal SMAD3 binding mediates TGF-β-dependent gene regulation in both contexts.

### Differential SMAD3 Binding Is Not the Sole Determinant of Context-Specific Gene Regulation by TGF-β

**Figure 2:**
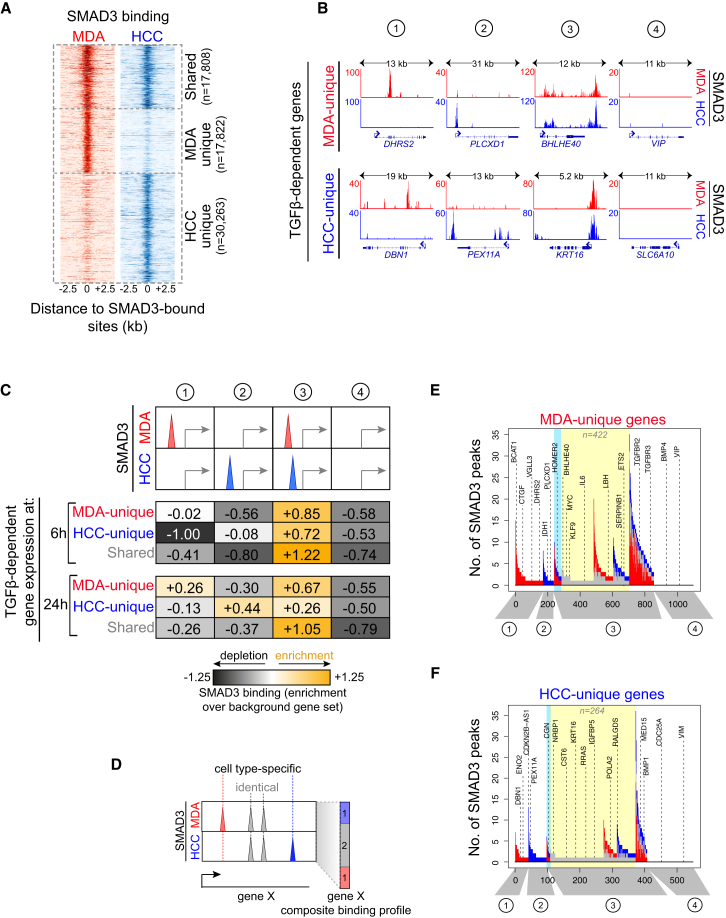
Differential SMAD3 Binding Is Not the Sole Determinant of Context-Specific Gene Regulation by TGF-β (A) Occupancy plots showing SMAD3 binding sites in MDA-MB-231 (red) and HCC-1954 (blue) BTICs relative to each other, within the 5-kb window around the peak summits. Also see Figure S1. (B) Gene tracks showing binding of SMAD3 in MDA-MB-231 (red) and HCC-1954 (blue) BTICs, at genes regulated by TGF-β only in MDA-MB-231 (top) and HCC-1954 (bottom) BTICs. SMAD3 adopts four modes of occupancy at these genes: bound in a cell-context-specific manner (modes 1 and 2), bound commonly in both BTIC types (mode 3), or not bound in either (mode 4). (C) Genome-wide analysis showing the enrichment of each of the four SMAD3 binding modes (from B) at TGF-β-dependent genes. Gene expression data from 6-hr and 24-hr time points were used. Enrichment was calculated over SMAD3 binding distribution in the TGF-β-independent, background gene set (see Supplemental Experimental Procedures for details). Note that the common binding mode (3) does not exclude SMAD3 binding sites that occur on the same genes but on different sites in the two BTICs. Also see Figure S2. (D) Schematic of the gene-based SMAD3 binding analysis. For each gene in the genome, the number of context-specific SMAD3 binding sites (red and blue) and shared binding sites (gray) were calculated and represented as a composite profile. (E and F) Gene-based SMAD3 binding analysis on context-specific TGFβ-dependent genes (performed as outlined in D). TGF-β-dependent genes 24 hr post-TGF-β stimulation were used. Genes are aligned along the x axis and grouped into distinct categories based on their SMAD3 composite profiles. SMAD3 binding modes are indicated below the plot in gray. Gene examples are highlighted with dashed lines. The light blue box marks genes with mutually exclusive SMAD3 binding patterns, and the yellow box marks those with predominantly similar or identical SMAD3 binding patterns in both BTICs. Also see Figure S2. See Supplemental Experimental Procedures for details.

The prevailing model for TGF-β context-dependent transcriptional regulation assumes binding of SMAD3 to different genes in different cell types (Massagué, 2012, Mullen et al., 2011). Our data showed instead that a substantial proportion of SMAD3 binding sites are identical in both BTIC types (50% in MDA-MB-231 and 37% in HCC-1954) (Figure 2A). Motif analysis identified a number of distinct DNA motifs under SMAD3 binding sites (Figures S1A and S1C), including “canonical” SMAD consensus motifs (Figures S1D and S1E) (Dennler et al., 1998, Jonk et al., 1998, Koinuma et al., 2009, Shi et al., 1998, Zawel et al., 1998). The majority of identified motifs also corresponded to known SMAD binding partners (Figure S1B), which have been implicated in TGF-β responses by single gene studies (Gomis et al., 2006, Koinuma et al., 2009, Liberati et al., 1999, Massagué, 2012, Sundqvist et al., 2013, Xu et al., 2015, Zaidi et al., 2002). These results indicate that SMAD3 associates with diverse co-factors that guide it to both shared and cell-type-specific genomic locations in BTICs.

Inspection of ChIP-seq profiles around BTIC context-specific TGF-β-dependent genes revealed that SMAD3 binding is not necessarily associated with the regulation of the underlying gene, but rather can adopt four different binding modes (Figure 2B). For example, a gene regulated by TGF-β only in MDA-MB-231 BTICs (MDA-unique gene) can be: (1) uniquely bound by SMAD3 in MDA-MB-231 (binding mode 1), (2) uniquely bound by SMAD3 in HCC-1954 (binding mode 2), (3) commonly bound by SMAD3 in both cell types (binding mode 3), and (4) not bound by SMAD3 in either cell type (binding mode 4). The same applies to the TGF-β-dependent genes regulated uniquely in HCC-1954 BTICs (HCC-unique genes) (Figure 2B, bottom panels). These results differ from those previously reported using non-malignant cellular models, where TGF-β’s cell-context-specific genes are almost exclusively associated with cell-type-specific SMAD3 binding patterns (Mullen et al., 2011).

We systematically investigated how these four SMAD3 binding modes contribute to gene regulation downstream of TGF-β. We found that TGF-β-dependent early-responder genes (derived 6 hr post-TGF-β treatment) are highly enriched for the common SMAD3 binding mode (mode 3) in both MDA-MB-231 and HCC-1954 BTICs (Figure 2C, upper table). TGF-β-dependent late-responder genes (derived 24 hr post-TGF-β treatment) show enrichment of both common (mode 3) and cell-type-unique SMAD3 binding (modes 1 and 2; Figure 2C, lower table). Notably, these BTIC type-unique SMAD3 binding events are associated with TGF-β-dependent genes in the corresponding model (MDA-unique genes with MDA-unique SMAD3 binding, and HCC-unique genes with HCC-unique SMAD3 binding) 24 hr after pathway activation. Genes not bound by SMAD3 in either cell type (mode 4) are relatively depleted in both the early and late TGF-β-responder genes, as could be expected based on the results presented in Figure 1E.

Based on the observed enrichment of the common SMAD3 binding mode in all gene groups and particularly in the early TGF-β responders, we conclude that cell-type-specific gene-proximal SMAD3 binding is not the sole determinant of context-specific TGF-β transcriptional responses.

In embryonic stem cells and muscle and lymphocyte progenitors, SMAD3 occupies distinct, non-overlapping sites within the gene, even when binding to the same gene (Mullen et al., 2011). To test if this also occurs in BTICs, we systematically categorized SMAD3 binding events into three classes: (1) uniquely present in MDA-MB-231 (Figure 2D, red peak), (2) uniquely present in HCC-1954 (Figure 2D, blue peak), and (3) present in identical position in both cell types (Figure 2D, gray peaks). For each context-specific TGF-β-dependent gene, we derived a composite SMAD3 binding profile (Figure 2D, right). This analysis revealed that only a small fraction of commonly bound genes (mode 3) possess mutually exclusive SMAD3 binding patterns (Figures 2E and 2F, light blue boxes). In fact, most genes that are commonly bound by SMAD3 (mode 3) display either a mixed occupancy profile, where both identical and cell-type-specific binding sites are present, or an identical occupancy profile, where SMAD3 binds at identical coordinates within a given gene in both cell types (Figures 2E and 2F). These findings led us to hypothesize that for many genes (at least 422 MDA-unique genes and 264 HCC-unique genes) possessing remarkably similar SMAD3 binding patterns in BTICs (yellow boxes, Figures 2E and 2F), other regulatory determinants might govern the context-specific transcriptional outputs of TGF-β.

We obtained similar results for SMAD3 binding events located distally to genes (Figures S2A–S2F; Supplemental Experimental Procedures); however, for simplicity, these are not presented in the Results section.

### Context-Specific Epigenetic Landscape Modulates TGF-β/SMAD3-Dependent Transcriptional Regulation

In breast cancer, epigenetic modifications have characteristic, subtype-specific genomic patterns (Bediaga et al., 2010, Holm et al., 2010). We therefore reasoned that cell-type-specific epigenetic landscapes in BTICs could contribute to shaping the TGF-β transcriptional responses. We profiled the chromatin configuration in BTICs by mapping RNA polymerase II (Pol II) binding, histone H3 lysine 27 acetylation (H3K27ac), histone H3 lysine 4 trimethylation (H3K4me3), and histone H3 lysine 27 trimethylation (H3K27me3) using ChIP-seq. We also mapped CpG DNA methylation using methyl-binding domain pull-down and sequencing (MBD-seq). These epigenetic marks were profiled in untreated BTIC cultures to determine whether the “native” chromatin configuration existing prior to TGF-β stimulation was what modulated the context-specific transcriptional response.

Peak-based analysis showed the genomic distribution of the epigenetic marks occurred in the expected patterns: Pol II peaks localized predominantly to enhancer and promoter regions, H3K4me3 peaks to promoter regions, H3K27ac peaks to enhancer and promoter regions, H3K27me3 peaks to intergenic domains, and DNA methylation peaks to gene-proximal elements (Figure S3A). Comparative analysis revealed that MDA-MB-231 and HCC-1954 BTICs harbor distinct epigenetic landscapes (Figure S3B).

Overlaying the epigenetic marks with SMAD3 binding data showed that SMAD3 binds to open chromatin (marked by H3K27ac, Pol II, and H3K4me3) and not to closed chromatin (marked by H3K27me3 and DNA methylation) (Figure S3C). Additionally, BTIC type-specific SMAD3 binding coincided with the type-specific patterns of Pol II and H3K27ac (Figure S3D). This suggested that the pre-existing cell-type-specific chromatin context determines where SMAD3 binds upon TGF-β stimulation.

**Figure 3:**
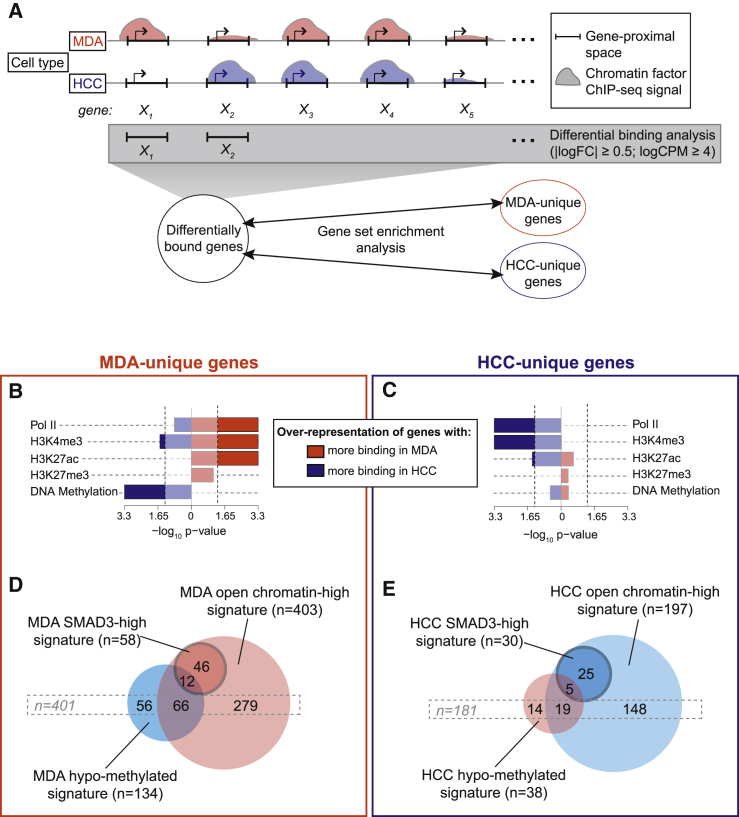
Epigenetic Wiring Confers Predisposition for Context-Specific TGF-β Responses (A) Schematic of the analysis approach. Differences between BTICs in the levels of each factor were defined based on differential binding analysis within the gene-proximal space (1,500 bp upstream of the TSS to gene end), apart from DNA methylation, for which only TSS-proximal regions were considered (−1,500 bp to +1,500 bp around the TSS). Gene set enrichment analysis was then conducted, testing the enrichment of differentially bound gene sets within the context-specific TGF-β-dependent gene sets (MDA-unique and HCC-unique genes). Refer to Supplemental Experimental Procedures for details. (B and C) Over-representation of genes with cell-type-specific levels of epigenetic modifications and Pol II, within the context-specific TGF-β-dependent genes (MDA-unique on the left and HCC-unique on the right). The significance of enrichment is represented as a p value on a bi-symmetrical x axis. The left and the right sides of the axis correspond to the enrichment of genes with more binding of the corresponding mark in HCC-1954 (blue) and MDA-MB-231 (red), respectively. p value cutoffs were set at 0.05 (−log_10_(1.33) = 0.05) (dashed lines). TGF-β-dependent genes derived at the 24-hr time point were used (see Supplemental Experimental Procedures for details). Also see Figure S3. (D and E) Comparison of SMAD3-high, open chromatin-high, and DNA hypo-methylation gene sets within the MDA-unique and HCC-unique TGF-β-dependent genes (24-hr gene expression time point). For each BTIC, SMAD3-high, open chromatin-high, and DNA hypo-methylation gene sets were defined as groups of genes with differentially higher SMAD3 levels, differentially higher open chromatin levels, and differentially lower DNA methylation levels when compared to the opposing BTIC type (differential binding analysis performed as in A). Also see Table S2.

We next asked whether the pre-existing BTIC type-specific gene-proximal chromatin patterns prime genes for TGF-β-mediated regulation. To address this question, we combined differential binding analysis with gene set enrichment analysis (Figure 3A). This revealed that context-specific TGF-β-dependent genes are enriched for those with cell-type-specific epigenetic patterns, characterized by higher levels of gene-proximal open chromatin marks (H3K4me3, H3K27ac, and Pol II) (Figures 3B and 3C) and lower levels of repressive chromatin marks in the corresponding BTIC type (Figure S3E; HCC-unique genes depleted from H3K27me3; MDA-unique genes depleted from DNA methylation). We also noted that TGF-β-dependent genes unique to MDA-MB-231 showed higher levels of DNA methylation in HCC-1954 (Figure 3B). Together, these results show that distinct epigenetic landscapes in BTICs modulate context-specific responses to TGF-β: high levels of H3K4me3, H3K27ac, and Pol II in gene-proximal space permit, while TSS DNA methylation and H3K27me3 impede, TGF-β/SMAD3-dependent regulation of gene expression.

**Figure 4:**
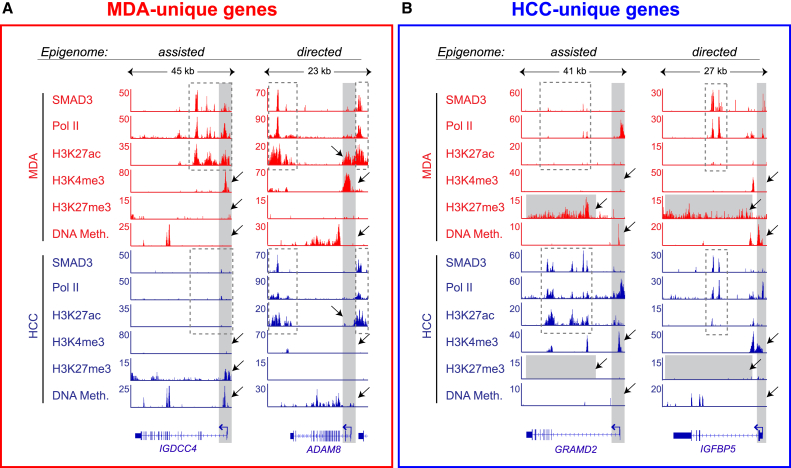
Epigenome either Assists or Directs Context-Specific Target Gene Regulation by TGF-β/SMAD3 (A and B) Examples illustrating the epigenome-assisted and epigenome-directed modes for target-gene regulation by TGF-β/SMAD3. ChIP-seq tracks for MDA-MB-231 are shown in red shades and for HCC-1954 in blue shades. Two MDA-unique (*IGDCC4* and *ADAM8*) and two HCC-unique (*GRAMD2* and *IGFBP5*) TGF-β-dependent genes are shown. In the epigenome-assisted mode, differential SMAD3 binding patterns (dashed boxes) are coupled with differential epigenetic configuration (gray boxes and arrows). In the epigenome-directed mode, SMAD3 binding patterns are the same in both BTICs (dashed boxes), while epigenetic differences (gray boxes and arrows) are associated with cell-type-specific gene regulation by TGF-β.

We next asked whether these epigenetic differences in the gene-proximal space act in synergy with, or independently of, differential SMAD3 binding to control context-specific TGF-β target gene regulation. For this purpose, genes with differential levels of SMAD3 were defined using the same analysis as for the chromatin factors (Figure 3A). This enabled us to stringently detect genes with the most pronounced differences in SMAD3 binding intensity between BTICs. For each TGF-β context-specific gene group (MDA unique and HCC unique), we derived three sets of signatures: SMAD3-high gene set (genes that display higher levels of SMAD3 in the corresponding BTIC type), open chromatin-high gene set (genes with higher levels of either H3K4me3, H3K27ac or Pol II in the corresponding BTIC type), and DNA hypo-methylation gene set (genes with lower levels of TSS DNA methylation in the corresponding BTIC type) (Table S2). Comparison of these gene sets in each BTIC type revealed that virtually all genes within the SMAD3-high set (58 in MDA and 30 in HCC) also belong to the open chromatin-high gene set (Figures 3D and 3E). This shows that in order to achieve type-specific gene regulation, differential binding of SMAD3 is assisted by gene-proximal open chromatin configuration, as shown for *IGDCC4* and *GRAMD2* (epigenome-assisted TGF-β-regulated genes; Figures 4A and 4B). Moreover, a substantial number of TGF-β-dependent genes in each BTIC type (401 MDA-unique genes; 181 HCC-unique genes) belonged to the open chromatin-high and/or DNA hypo-methylation sets, but not to the SMAD3-high set. Hence, the context-specific TGF-β-dependent regulation of the genes in this set is likely to be mediated by epigenetic differences (epigenome-directed TGF-β-regulated genes), as highlighted by *ADAM8* and *IGFBP5* (Figures 4A and 4B). This analysis also revealed that only a subset of SMAD3-high genes overlap with the DNA hypo-methylated set, suggesting that differential DNA methylation and differential SMAD3 binding appear to independently contribute to context-specific gene regulation by TGF-β.

Taken together, these results suggest that cell context-specific transcriptional responses to TGF-β are mediated by both SMAD3 and the epigenome. The epigenomic landscape primes genes for transcriptional regulation by TGF-β signaling, both in synergy with, and independently of, differential SMAD3 binding.

### Differential DNA Methylation of *LBH* Impacts the BTIC-Promoting Effects of TGF-β

To functionally validate the impact of the epigenome on the opposing effects of TGF-β on BTICs, we focused on context-specific TGF-β-dependent genes with differential DNA methylation. Interesting links have been proposed between normal developmental processes and breast cancer (Holm et al., 2010, Prat et al., 2010), and therefore, we selected two genes encoding developmental TFs for further analysis: Limb Bud and Heart Development (*LBH*), and Vestigial-like family member 3 (*VGLL3*).

**Figure 5:**
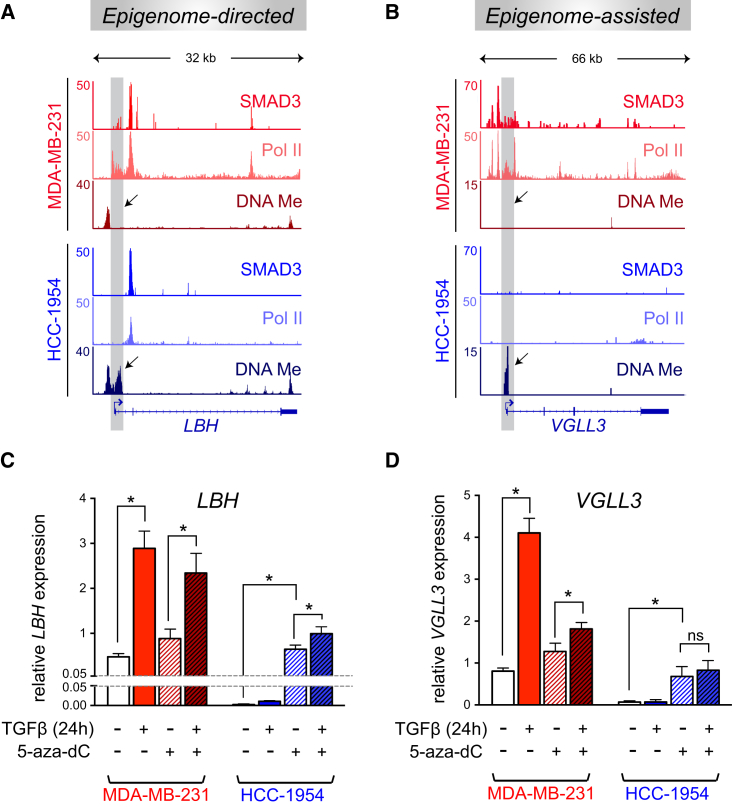
DNA Methylation Regulates TGF-β-Dependent Induction of *LBH* (A and B) Gene tracks showing SMAD3/Pol II binding and DNA methylation at *LBH* and *VGLL3* loci. ChIP-seq tracks for MDA-MB-231 are shown in red shades and for HCC-1954 in blue shades. Also see Figure S4. (C and D) qRT-PCRs showing the expression of *LBH* and *VGLL3* transcripts upon 5-aza-dC and TGF-β treatments. Cells were treated with 5-aza-dC for 5 days, seeded, and allowed to form mammospheres for 7 days, and then stimulated with TGF-β for 24 hr. Data were normalized to the housekeeping (*RBM22*) transcript levels and are presented as mean ± SD of three biological replicates. Asterisks indicate significant differences. ns, not significant (one-way ANOVA). Also see Figure S4.

*LBH* and *VGLL3* are induced by TGF-β in a SMAD2/3 dependent manner in BTICs from MDA-MB-231, but not in HCC-1954 (Figures S4A–S4D). *LBH* is bound by SMAD3, and Pol II at an identical intragenic regulatory region in both cell types (Figure 5A), but in HCC-1954, the TSS-proximal region is DNA methylated (coinciding with lack of Pol II binding) (Figure 5A). This suggests that context-specific regulation of *LBH*, despite remarkably similar SMAD3 binding, is dependent on the methylation status of its promoter (epigenome directed). In contrast, *VGLL3* is bound by SMAD3 and Pol II only in MDA-MB-231 BTICs, while its TSS harbors DNA methylation only in HCC-1954 (Figure 5B). Hence, TGF-β-dependent regulation of *VGLL3* is epigenome assisted.

To test if promoter methylation of *LBH* and *VGLL3* determines their context-specific TGF-β-dependent transcriptional regulation, BTICs were treated with 5-aza-2′-deoxycytidine (5-aza-dC) prior to TGF-β stimulation, which resulted in reduction of overall methylation levels at these loci in HCC-1954 (Figures S4F and S4G). In HCC-1954, 5-aza-dC treatment reactivated both *LBH* and *VGLL3* expression, and TGF-β treatment further induced *LBH,* but not *VGLL3* (Figures 5C, 5D, and S4E). This shows that promoter DNA methylation is sufficient to block TGF-β/SMAD3-mediated induction of *LBH*. Erasure of DNA methylation from *VGLL3* failed to restore its TGF-β-dependent induction in HCC-1954, as predicted due to the absence of SMAD3 binding at this locus. Taken together, these results confirm that the epigenetic configuration not only determines baseline gene expression levels, but it also controls TGF-β/SMAD3-dependent transcriptional regulation.

To assess the functional implications of epigenome-directed and epigenome-assisted mechanisms, we investigated whether *LBH* and *VGLL3* are required for the effects of TGF-β on BTICs. We knocked down their expression using short interfering RNAs (siRNAs), resulting in 80% and 50% reduction of *LBH* and *VGLL3* transcript levels, respectively (Figures S5A and S5B). Mammosphere-initiating cell (MS-IC) and colony-forming cell (CFC) assays were used to test self-renewal and proliferation of BTICs (Bruna et al., 2012, Dontu et al., 2003a, Dontu et al., 2003b).

**Figure 6:**
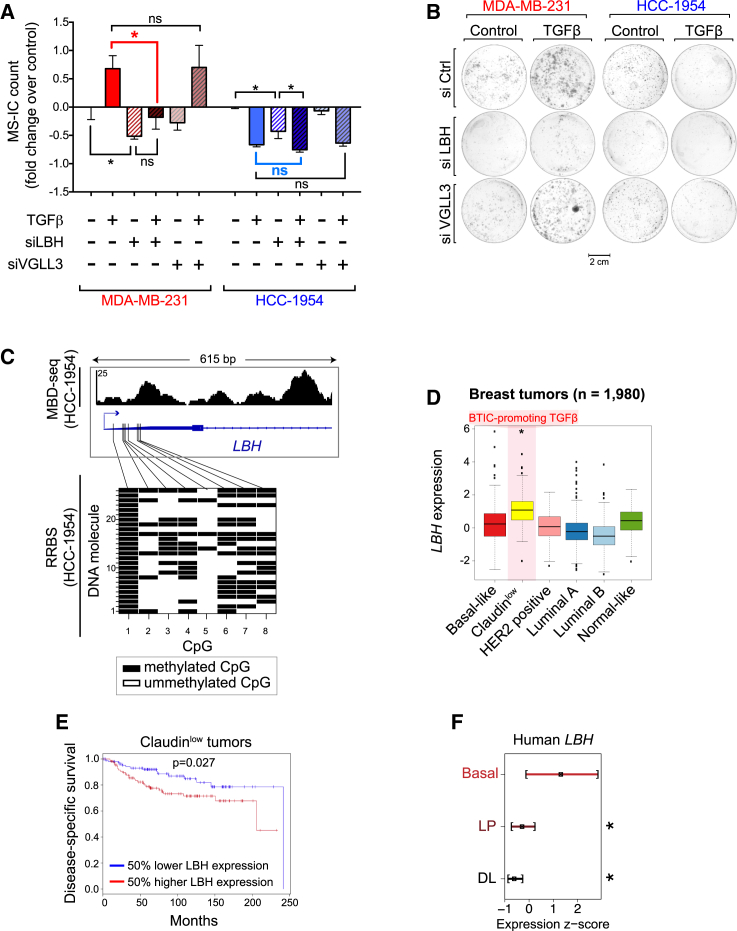
*LBH* Is Necessary for TGF-β’s BTIC-Promoting Activity (A) MS-IC assay showing the changes in MS-IC numbers upon TGF-β treatment and siRNA-mediated knockdown of *LBH* and *VGLL3*. Mammospheres were simultaneously treated with TGF-β and siRNA against *LBH* and *VGLL3* for 7 days, second-generation mammospheres were seeded, and an MS-IC assay was performed (see Supplemental Experimental Procedures). Data are presented as mean ± 95% confidence interval (CI) of nine replicates. Asterisks indicate significant differences. ns, not significant (one-way ANOVA). Also see Figure S5. (B) CFC assay showing the effects of TGF-β and siRNA-mediated knockdown of *LBH* and *VGLL3* on the proliferation of BTICs. (C) RRBS analysis of the *LBH* promoter. x axis shows eight adjacent CpG sites within the *LBH* promoter (highlighted on top); y axis shows binary methylation calls for each CpG site within 26 sequenced DNA molecules. Genomic track on top represents the DNA methylation profile of HCC-1954 BTICs derived by MBD-seq. (D) Box plots showing the expression of *LBH* in different breast cancer subtypes. Significance was determined by a linear model (ANOVA) and simultaneous tests comparing each group to the mean (see Supplemental Experimental Procedures). Gene expression data are obtained from the METABRIC cohort (Curtis et al., 2012). (E) Survival analysis showing the relationship between *LBH* expression and disease-free survival in the Claudin^low^ patient group. Patients were stratified based on top and bottom halves of *LBH* expression. Survival function was estimated using the Kaplan-Meier estimator, and differences between groups were tested with the log-rank test (see Supplemental Experimental Procedures). (F) Expression of *LBH* in different cell compartments of normal human mammary epithelium. Basal compartment, luminal progenitors (LP), and differentiated luminal cells (DL) are shown. Significance was determined by a linear model (ANOVA) comparing LP and DL expression to the basal group. Data from Shehata et al. (2012). Also see Figure S5.

*LBH* knockdown in untreated cells reduced BTIC self-renewal and proliferation in both cell lines (Figures 6A and 6B), suggesting that *LBH* is required for baseline BTIC maintenance regardless of the response to TGF-β. In HCC-1954 BTICs, *LBH* transcripts are expressed at very low levels despite promoter methylation, and their reduction by siRNA treatment (Figure S5A) results in measurable effects in the BTIC assays (Figures 6A and 6B). These *LBH* transcripts are likely to originate from low levels of transcription initiated at methylated DNA molecules with variegated CpG methylation patterns (“epipolymorphisms”; Landan et al., 2012), as determined by reduced representation bisulfite sequencing (RRBS) (Figure 6C). Thus, residual transcription initiated at epipolymorphic promoters can be functionally important.

In MDA-MB-231, *LBH* depletion impaired the BTIC-promoting effects of TGF-β by more than 2-fold (Figures 6A and 6B). In contrast, in HCC-1954, *LBH* depletion did not affect BTIC suppression by TGF-β (Figures 6A and 6B). In both cells lines, *VGLL3* depletion had no effect on BTICs after TGF-β treatment (Figures 6A and 6B). Altogether, these results suggest that epigenome-directed gene co-regulation with SMAD3, as occurs with *LBH*, acts as a molecular switch that mediates the opposing effects of TGF-β on BTICs.

We previously showed that TGF-β specifically promotes BTIC activity only in Claudin^low^ cell lines (Bruna et al., 2012). We also showed that in normal mammary epithelium, TGF-β promotes mammary stem cells (the presumed cell of origin of Claudin^low^ cancers), and it inhibits luminal progenitors (Bruna et al., 2012). Interestingly, others have shown that in normal breast epithelial tissue, *LBH* promotes stemness and inhibits differentiation (Lindley et al., 2015, Rieger et al., 2010). We therefore sought evidence for a relevant role of *LBH* in both normal breast epithelium and in breast cancer. Analysis of gene expression data from normal human and mouse mammary epithelium revealed that *LBH* is highly expressed in the basal (stem cell-containing) compartment and is downregulated as cells differentiate along the luminal lineage (Figures 6F, S5C, and S5D). Investigation of gene expression data from 1,980 primary breast cancers (Curtis et al., 2012) showed that *LBH* expression is highest in the Claudin^low^ subtype (Figure 6D). In patients with Claudin^low^ tumors, higher *LBH* expression correlates with worse survival (Figure 6E). These findings suggest that the BTIC context-specific TGF-β/*LBH* observations we made in model cell lines are relevant to both normal and malignant primary tissue biology.

## Discussion

The mechanisms underlying the opposing TGF-β effects in cancer cells, being both pro-oncogenic and tumor suppressive, remain a significant challenge for inhibition of the pathway as a feasible cancer therapeutic strategy in the clinic. The current understanding is that TGF-β stimulation results in different responses in distinct cell types through the association of SMAD2/3 with specific SMAD cofactors (Massagué, 2008, Massagué, 2012). Accordingly, in normal cells, along a developmental cascade, SMAD3 co-occupies distinct genomic locations in association with cell-type-specific master transcription factors: Oct4 in embryonic stem cells, Myod1 in myotubes, and PU.1 in pro-B cells (Mullen et al., 2011). These cofactors are required for SMAD3 binding, and most TGF-β-regulated genes are bound by these master TFs (Mullen et al., 2011). In other words, the currently accepted model suggests that master TFs are responsible for instructing the gene targets downstream of TGF-β signaling and thus determine its cell-type-specific effects (Mullen et al., 2011). In cancer cells, no similar genome-wide studies have been conducted, but single-gene studies appear to show analogous findings: TF switches (where SMADs exchange binding partners) can occur and result in redirecting of SMADs from the promoters of tumor suppressor genes to promoters of oncogenes, concomitant with altered transcription of those target genes (Gomis et al., 2006, Seoane et al., 2004, Xu et al., 2015).

Our results show for the first time that different SMAD3 binding patterns cannot fully account for the observed differences in the TGF-β-dependent transcriptional responses associated with promotion or suppression of BTICs. In fact, and surprisingly, the majority of BTIC context-specific TGF-β-dependent genes, particularly early-responder genes, are bound by SMAD3 in both contexts. While binding may occur in distinct locations along the gene, a large fraction of genes possessed coherent SMAD3 occupancy profiles, many with only identical SMAD3 binding sites. These results reveal that TGF-β-dependent cell-type-specific transcriptional regulation in cancer cells is not universally mediated by differential SMAD3 binding. This prompted us to analyze whether additional regulatory mechanisms operating on chromatin modulate the context-specific target gene selection by TGF-β/SMAD3.

**Figure 7:**
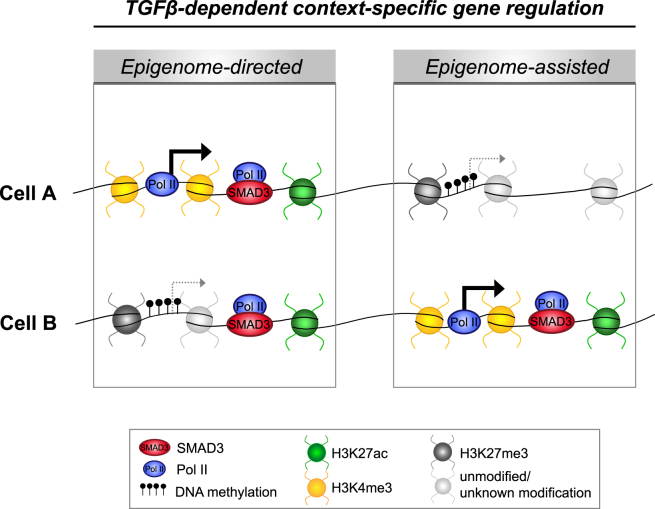
Context-Specific Effects of TGF-β/SMAD3 Are Modulated by the Epigenome A model depicting how cell-type-specific epigenetic configurations determine context-specific effects of TGF-β/SMAD3. Genes differentially bound by SMAD3 also possess differential levels of open and closed chromatin modifications that will participate in specifying TGF-β-dependent expression of those genes (epigenome-assisted mechanism). Genes commonly bound by SMAD3 rely on the underlying cell-type-specific epigenetic configuration for determining their context-specific regulation by TGF-β (epigenome-directed mechanism).

Very recently, it has been reported that epigenetic configuration of somatic cells predisposes them to reprogramming fates (Pour et al., 2015). Here we show that tumor initiating cells harbor distinct epigenetic landscapes that prime specific gene sets for regulation by TGFβ. These distinct epigenetic configurations can act both in synergy with cell-type-specific SMAD3 binding (epigenome-assisted), and independently of cell-type-specific SMAD3 binding (epigenome-directed), to control TGFβ/SMAD3-dependent context-specific regulation of target genes (Figure 7).

We propose that epigenome-directed priming in cancer cells might be a prevalent way of instructing context-specific TGFβ effects. Cancer cells that originate in the same tissue (mammary epithelium in the case of BTICs), unlike cells from distinct tissue lineages, are likely to possess similar master TF wiring. But cancers with the same tissue of origin can possess markedly different epigenomes, for example, DNA methylation of gene promoters (Holm et al., 2010). Here, we reveal an unexpected similarity of SMAD3 binding patterns in BTICs with opposing transcriptional responses to TGF-β and show that context-specific TGF-β-dependent genes are frequently regulated by an epigenome-directed, DNA-methylation-dependent mechanism, rather than by differential SMAD3 binding. These results at the whole-genome level expand a previous observation in glioma, where the methylation status of *PDGFB* predisposes tumor cells for either an oncogenic or a tumor-suppressive response to TGF-β signaling (Bruna et al., 2007).

We have identified *LBH*, a regulator of epithelial differentiation in the mammary gland (Lindley et al., 2015, Rieger et al., 2010), as a mediator required for the context-specific BTIC-promoting effects of TGF-β, depending on its cell-type-specific methylation state. The patterns of expression of *LBH* in normal mammary development and in human breast cancers are consistent with its role as a context-specific TGF-β target in primary tissues. We speculate that many epigenome-directed genes behave like *LBH* to mediate the context-specific effects of TGF-β in cancer.

The model we propose here (Figure 7), that regulation of transcriptional programs by extracellular growth factors is dependent on the context-specific epigenomic landscapes of cancer cells, might not be specific to TGF-β and could have broader implications for the paracrine effects of the microenvironment on the malignant compartment of cancers.

## Experimental Procedures

### Cell Manipulation and Mammosphere Cultures

MDA-MB-231 and HCC-1954 breast cancer cell lines were enriched for BTICs by mammosphere cultures, as described previously (Bruna et al., 2012, Dontu et al., 2003a, Dontu et al., 2003b). To activate TGF-β signaling, mammospheres were treated with 0.1 nM recombinant TGF-β1. *LBH*, *VGLL3*, *SMAD2* and *SMAD3* levels were manipulated using siRNA pools (GE Healthcare). To achieve global DNA demethylation, the cells were treated with 1 μM 5-aza-2′-deoxycytidine. For full details, see Supplemental Experimental Procedures.

### Chromatin Immunoprecipitation and Sequencing

ChIP-seq was performed using a custom-developed protocol. Briefly, mammospheres (treated with 0.1 nM TGF-β for 3 hr for SMAD3 ChIP-seq, and untreated in all other experiments) were crosslinked for 45 min with Di(N-succinimidyl) glutarate (DSG) and 30 min with formaldehyde. Chromatin was extracted and then sheared using Covaris. Immunoprecipitation was performed with 10 μg of the corresponding antibodies and protein G agarose beads (Santa Cruz Biotechnology). Libraries were prepared with TruSeq LT kit (Illumina) and sequenced on HiSeq 2000 (Illumina). For the full protocol, see Supplemental Experimental Procedures.

### DNA Methylation Profiling

For MBD-seq, methylated DNA was precipitated with recombinant methyl binding domain (MBD2b/MBD3L1) protein complex as part of MethylCollector Ultra kit (Active Motif), following the manufacturer’s recommendations. Libraries were generated using TruSeq LT kit (Illumina) and sequenced on HiSeq 2000 (Illumina). Refer to Supplemental Experimental Procedures for details.

RRBS was performed as described previously (Boyle et al., 2012).

Targeted bisulfite sequencing was performed using a custom-developed method (refer to Supplemental Experimental Procedures for details).

### ChIP-Seq and MBD-Seq Data Analysis

Sequencing reads were filtered based on quality and aligned to the Human Genome Build 37 (hg19) using BWA (Li and Durbin, 2009). For ChIP-seq, SMAD3 peaks were called using MACS (Zhang et al., 2008), and SICER (Zang et al., 2009) was used for all other factors profiled. For MBD-seq, bi-asymmetric-Laplace model (BALM) was used to call methylation peaks (Lan et al., 2011), and (MeD)IP-seq data analysis (MEDIPS) was used for quantitative analysis, whereby the data were normalized to the CG content (Lienhard et al., 2014). Downstream analysis of all datasets was performed in R statistical software (R Development Core Team, 2009), using edgeR (v3.8.5) for differential binding analysis (Robinson et al., 2010) and annovar (2014nov12) for annotation (Wang et al., 2010). Motif analysis was performed in MEME-ChIP (Machanick and Bailey, 2011). Tracks representing genomic data were derived from IGV (Robinson et al., 2011). Refer to Supplemental Experimental Procedures for details.

### Gene Expression Analysis

Gene expression upon TGF-β induction was profiled using Illumina HumanHT-12 BeadChips. Data were analyzed as previously described (Smyth, 2005). Refer to Supplemental Experimental Procedures for details.

### qRT-PCR

Reverse transcription was performed using Transcriptor First Strand cDNA Synthesis Kit (Roche), as recommended by the manufacturer. qPCR was performed using TaqMan Fast Universal PCR Master Mix and gene-specific TaqMan probes (Applied Biosystems). Refer to Supplemental Experimental Procedures for details.

### Western Blots

The cells were grown as mammospheres for 7 days then treated with TGF-β for 1 hr. Total cell lysates were collected, and 20 μg protein was run per condition on the 10% SDS-PAGE gels. Transfer to nitrocellulose membranes was conducted using semi-dry blotting system (Invitrogen). Membranes were blocked in 5% dried milk powder, 0.1% Tween-20 in PBS, and the following antibodies were used for protein detection: rabbit monoclonal against human phospho-SMAD2 (Ser 465/467) (Cell Signaling, 3108), rabbit polyclonal against human SMAD2/3 (Santa Cruz Biotechnology, sc-8332) and rabbit polyclonal against human β-actin (Abcam, ab8227).

### Mammosphere-Initiating Cell and Colony-Forming Cell Assays

MS-IC and CFC assays were performed as previously described (Dontu et al., 2003a, Dontu et al., 2003b). Refer to Supplemental Experimental Procedures for details.

## Author Contributions

C.C. and A.B. supervised the study. A.T.V., A.B., and C.C. conceived and designed experiments. A.T.V., S.J.V., A.S.B., M.A.G., S.U.-L., and W.G. performed experiments. O.M.R. and A.T.V. performed data analysis. A.T.V., A.B., C.C., and P.J.C. interpreted experiments. A.T.V., C.C., and A.B. wrote the manuscript, incorporating edits from all authors.

## Accession Numbers

The accession number for the ChIP-seq, MBD-seq, and gene expression data reported in this paper is EGA: EGAS00001001570.

## Supplemental Information

Document S1. Supplemental Experimental Procedures and Figures S1–S5

Table S1. Dynamics of TGF-β-Mediated Gene Expression in BTICs, Related to Figure 1 An Excel file providing the lists of TGFβ-dependent genes at four different time points (1h, 3h, 6h and 24h) upon pathway induction, in MDA-MB-231 and HCC-1954 BTICs.

Table S2. SMAD3-High, Open Chromatin-High, and DNA Hypo-methylation Gene Sets, Related to Figure 3 An Excel file providing the lists of genes in the SMAD3-high, open chromatin-high and DNA hypomethylation sets for each BTIC type.

Document S2. Article plus Supplemental Information
