# Supplementary material for: Context-Specific Effects of TGF-β/SMAD3 in Cancer Are Modulated by the Epigenome
Source: Cell Rep. 2015 Dec 10;13(11):2480–90. doi: 10.1016/j.celrep.2015.11.040 (PMC4695334; doi:10.1016/j.celrep.2015.11.040)
Supplement: Document S1. Supplemental Experimental Procedures and Figures S1–S5 [file mmc1.pdf]

Cell Reports

Supplemental Information

Context-Specific Effects of TGF-β/SMAD3 in Cancer Are Modulated by the Epigenome

Ana Tufegdzic Vidakovic, Oscar M. Rueda, Stephin J. Vervoort, Ankita Sati Batra, Mae Akilina Goldgraben, Santiago Uribe-Lewis, Wendy Greenwood, Paul J. Coffer, Alejandra Bruna, and Carlos Caldas

Figure S1

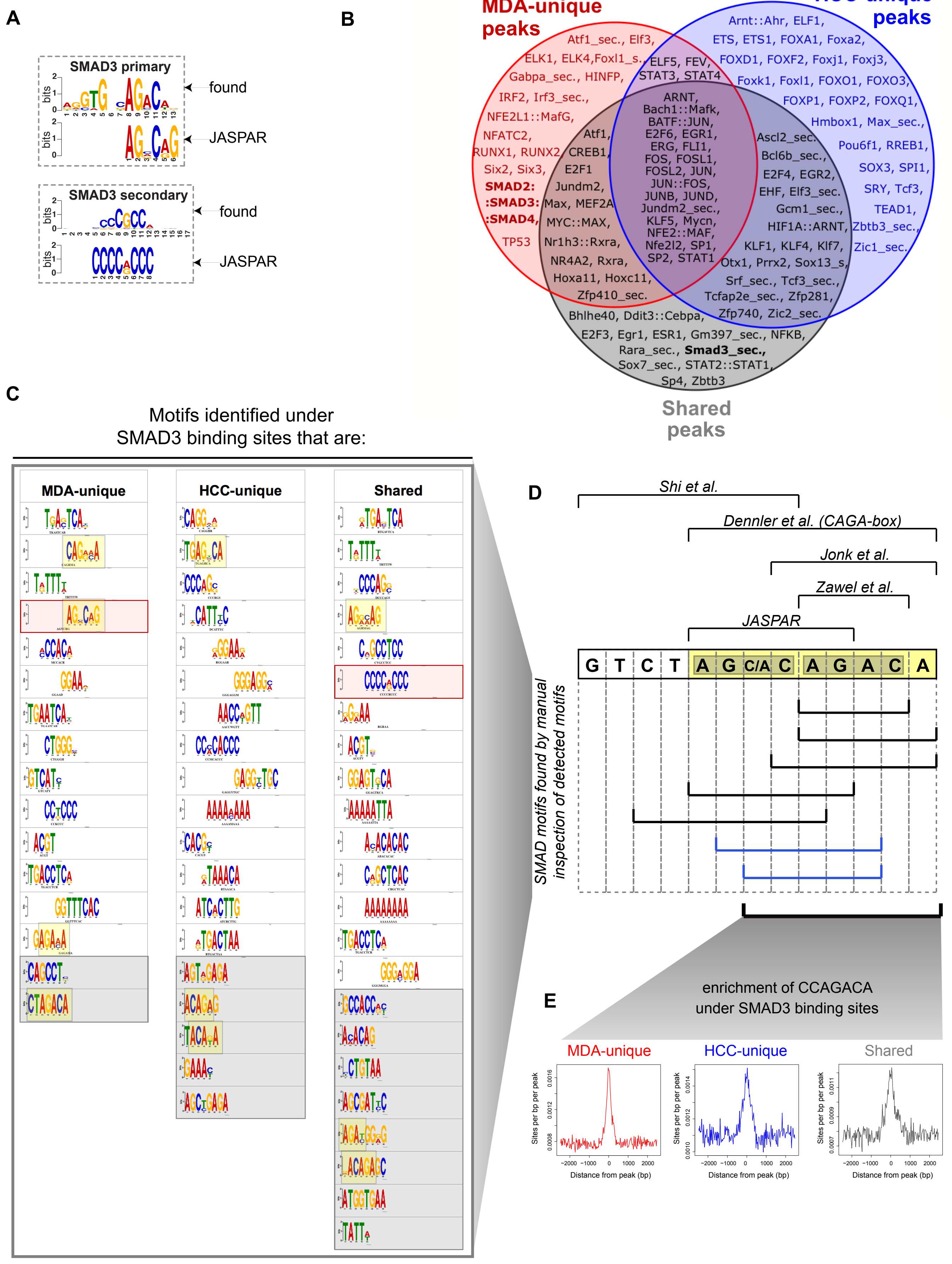
A
SMAD3 primary
found
JASPAR
SMAD3 secondary
found
JASPAR
B
MDA-unique peaks
HCC-unique peaks
Shared peaks
Atf1_sec., Elf3, ELK1, ELK4,Foxl1_s., Gabpa_sec., HINFP, IRF2, Irf3_sec., NFE2L1::MafG, NFATC2, RUNX1, RUNX2, Six2, Six3, SMAD2::SMAD3::SMAD4, TP53
ELF5, FEV, STAT3, STAT4
Arnt::Ahr, ELF1, ETS, ETS1, FOXA1, Foxa2, FOXD1, FOXF2, Foxj1, Foxj3, Foxk1, Foxl1, FOXO1, FOXO3, FOXP1, FOXP2, FOXQ1, Hmbox1, Max_sec., Pou6f1, RREB1, SOX3, SPI1, SRY, Tcf3, TEAD1, Zbtb3_sec., Zic1_sec.
ARNT, Bach1::Mafk, BATF::JUN, E2F6, EGR1, ERG, FLI1, FOS, FOSL1, FOSL2, JUN, JUN::FOS, JUNB, JUND, Jundm2_sec., KLF5, Mycn, NFE2::MAF, Nfe2l2, SP1, SP2, STAT1
Atf1, CREB1, E2F1, Jundm2, Max, MEF2A, MYC::MAX, Nr1h3::Rxra, NR4A2, Rxra, Hoxa11, Hoxc11, Zfp410_sec.
Ascl2_sec., Bcl6b_sec., E2F4, EGR2, EHF, Elf3_sec., Gcm1_sec., HIF1A::ARNT, KLF1, KLF4, Klf7, Otx1, Prrx2, Sox13_s, Srf_sec., Tcf3_sec., Tcfap2e_sec., Zfp281, Zfp740, Zic2_sec.
Bhlhe40, Ddit3::Cebpa, E2F3, Egr1, ESR1, Gm397_sec., NFKB, Rara_sec., Smad3_sec., Sox7_sec., STAT2::STAT1, Sp4, Zbtb3
C
Motifs identified under SMAD3 binding sites that are:
MDA-unique
HCC-unique
Shared
D
Shi et al.
Dennler et al. (CAGA-box)
Jonk et al.
Zawel et al.
JASPAR
G T C T A G C/A C A G A C A
SMAD motifs found by manual inspection of detected motifs
E
enrichment of CCAGACA under SMAD3 binding sites
MDA-unique
HCC-unique
Shared
Sites per bp per peak
Distance from peak (bp)
-2000 -1000 0 1000 2000

Figure S2

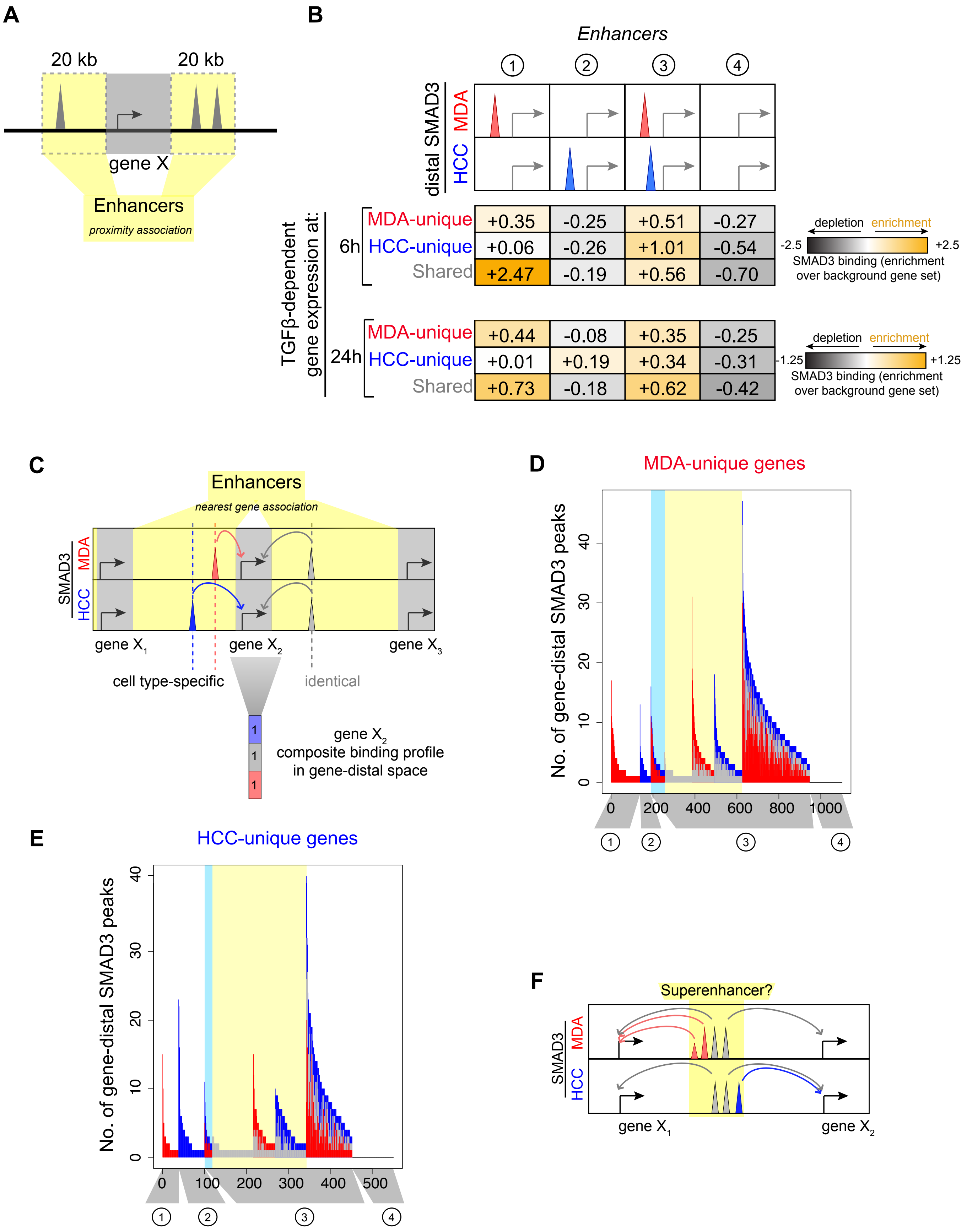
A
20 kb
20 kb
gene X
Enhancers
proximity association
B
Enhancers
1
2
3
4
distal SMAD3
MDA
HCC
TGFβ-dependent gene expression at:
6h
MDA-unique +0.35 -0.25 +0.51 -0.27
HCC-unique +0.06 -0.26 +1.01 -0.54
Shared +2.47 -0.19 +0.56 -0.70
24h
MDA-unique +0.44 -0.08 +0.35 -0.25
HCC-unique +0.01 +0.19 +0.34 -0.31
Shared +0.73 -0.18 +0.62 -0.42
depletion
enrichment
-2.5
+2.5
SMAD3 binding (enrichment over background gene set)
depletion
enrichment
-1.25
+1.25
SMAD3 binding (enrichment over background gene set)
C
Enhancers
nearest gene association
SMAD3
MDA
HCC
gene X1
gene X2
gene X3
cell type-specific
identical
1
1
1
gene X2 composite binding profile in gene-distal space
D
MDA-unique genes
No. of gene-distal SMAD3 peaks
0
10
20
30
40
0
200
400
600
800
1000
1
2
3
4
E
HCC-unique genes
No. of gene-distal SMAD3 peaks
0
10
20
30
40
0
100
200
300
400
500
1
2
3
4
F
Superenhancer?
SMAD3
MDA
HCC
gene X1
gene X2

Figure S3

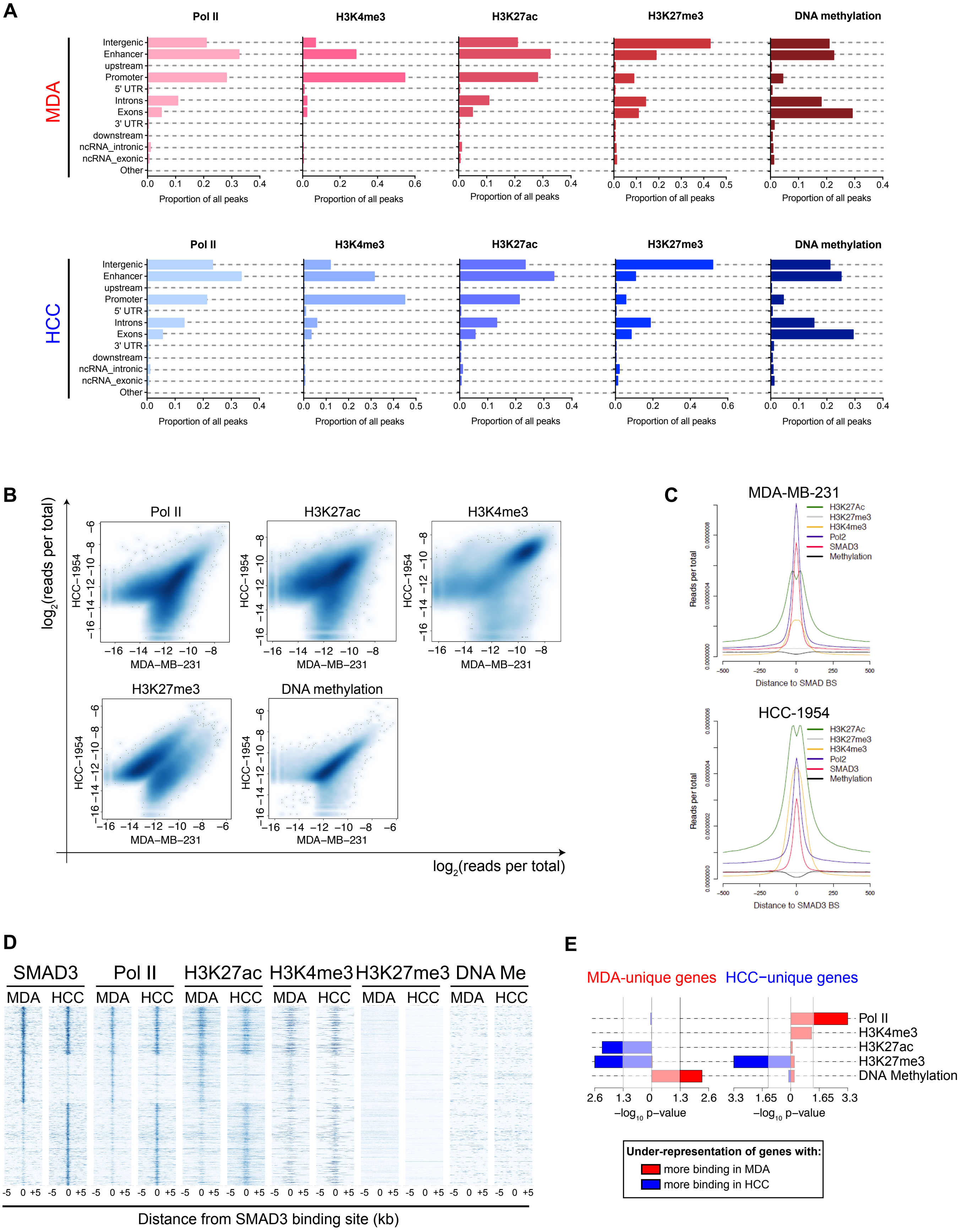

A
MDA
HCC
Pol II
H3K4me3
H3K27ac
H3K27me3
DNA methylation
Intergenic
Enhancer
upstream
Promoter
5' UTR
Introns
Exons
3' UTR
downstream
ncRNA_intronic
ncRNA_exonic
Other
Proportion of all peaks
B
log2(reads per total)
Pol II
H3K27ac
H3K4me3
H3K27me3
DNA methylation
HCC−1954
MDA−MB−231
C
MDA-MB-231
HCC-1954
H3K27Ac
H3K27me3
H3K4me3
Pol2
SMAD3
Methylation
Reads per total
Distance to SMAD BS
Distance to SMAD3 BS
D
SMAD3
Pol II
H3K27ac
H3K4me3
H3K27me3
DNA Me
MDA
HCC
-5 0 +5
Distance from SMAD3 binding site (kb)
E
MDA-unique genes
HCC−unique genes
Pol II
H3K4me3
H3K27ac
H3K27me3
DNA Methylation
2.6 1.3 0 1.3 2.6
3.3 1.65 0 1.65 3.3
−log10 p−value
Under-representation of genes with:
more binding in MDA
more binding in HCC

Figure S4

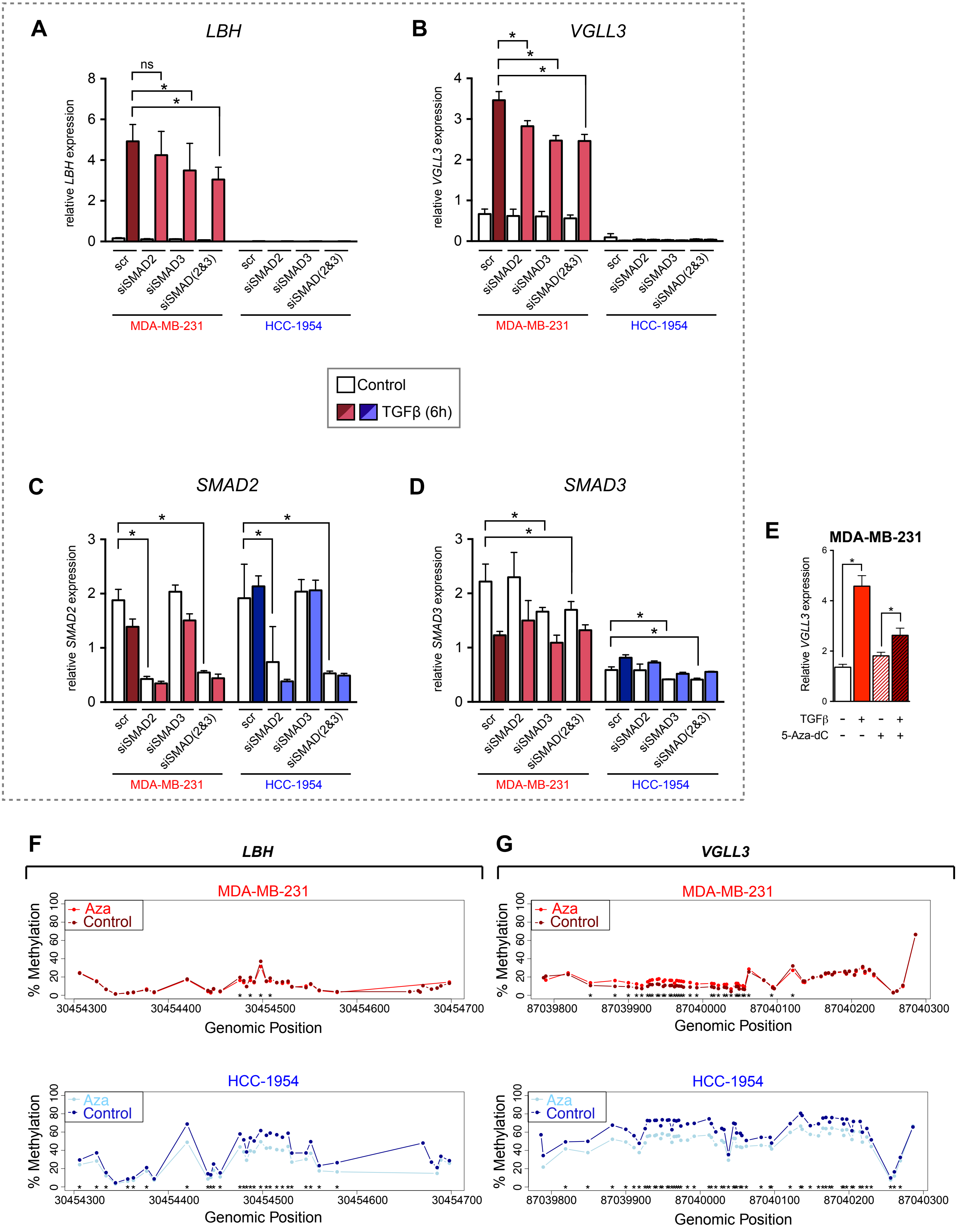
A
LBH
relative LBH expression
ns
scr
siSMAD2
siSMAD3
siSMAD(2&3)
MDA-MB-231
HCC-1954
B
VGLL3
relative VGLL3 expression
Control
TGFβ (6h)
C
SMAD2
relative SMAD2 expression
D
SMAD3
relative SMAD3 expression
E
MDA-MB-231
Relative VGLL3 expression
TGFβ
5-Aza-dC
F
LBH
% Methylation
Aza
Control
Genomic Position
G
VGLL3

Figure S5

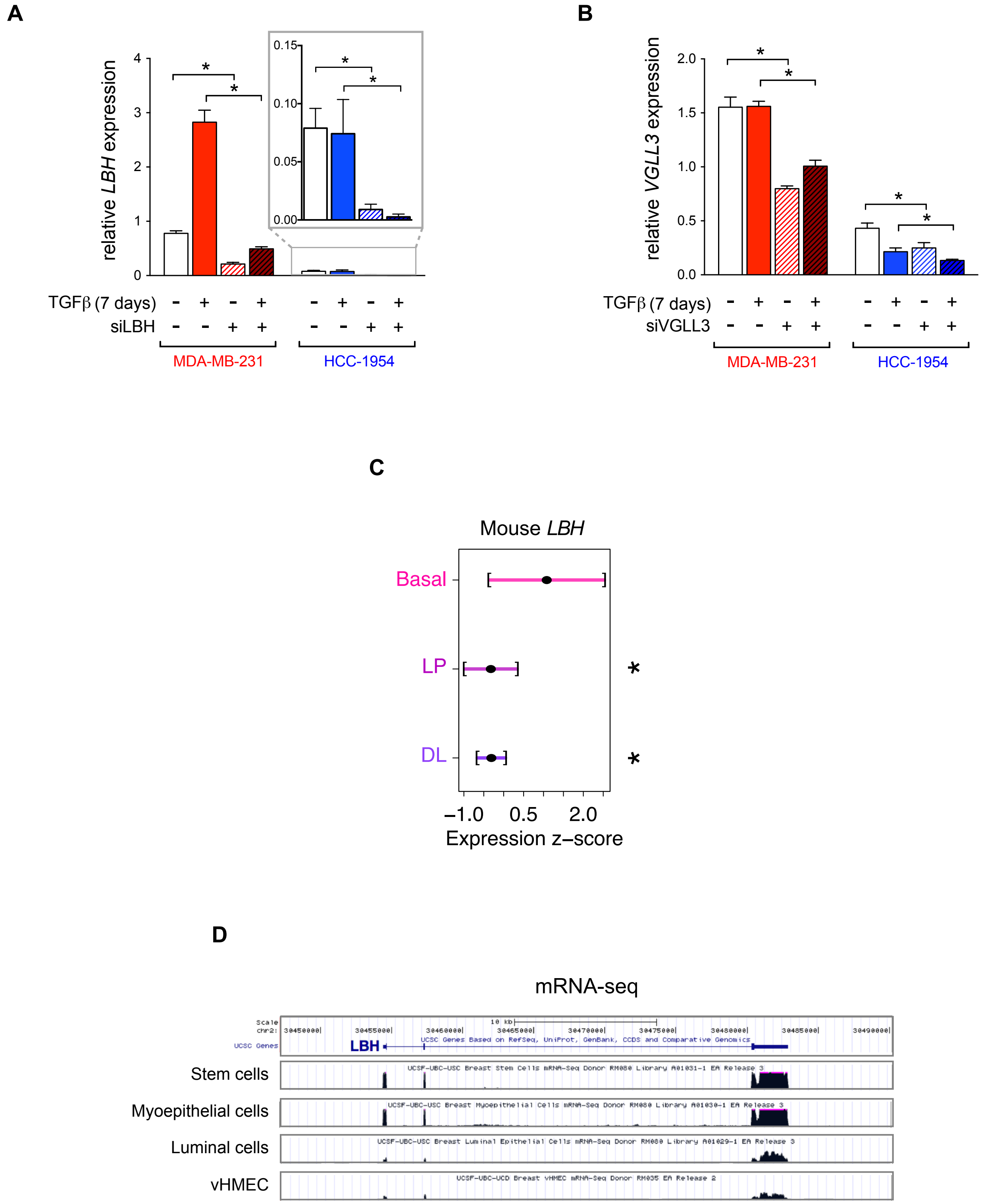
A
relative LBH expression
0
1
2
3
4
*
*
0.15
0.10
0.05
0.00
TGFβ (7 days)
siLBH
- + - +
- - + +
MDA-MB-231
HCC-1954
B
relative VGLL3 expression
0.0
0.5
1.0
1.5
2.0
TGFβ (7 days)
siVGLL3
MDA-MB-231
HCC-1954
C
Mouse LBH
Basal
LP
DL
*
*
−1.0
0.5
2.0
Expression z−score
D
mRNA-seq
LBH
UCSC Genes
Stem cells
Myoepithelial cells
Luminal cells
vHMEC

Supplemental Figure Legends

Figure S1. SMAD3 Binds to Multiple Diverse DNA Motifs in BTICs. Related to Figure 2.

(A) Identification of SMAD3 motifs in the SMAD3 ChIP-seq datasets. Motif analysis was performed in MEME.

(B) Putative SMAD3 partner TFs corresponding to DNA motifs detected in C (see below). Each circle represents putative co-factors (identified in MEME) whose motifs were found under MDA-unique (red), HCC-unique (blue) or Shared (grey) SMAD3 peaks. Note that even cell type-unique SMAD3 binding sites (peaks) can possess similar or identical DNA motifs, to which then identical TFs could bind, resulting in the overlap of the circles.

(C) Distinct DNA motifs detected under MDA-MB-231-unique SMAD3 binding sites, HCC-1954-unique SMAD3 binding sites and shared SMAD3 binding sites. Red boxes indicate primary and secondary SMAD3 motifs identified by MEME, yellow boxes mark manually identified SMAD-binding elements, whereas grey boxes indicate motifs unknown to associate with any factors to date. Motif enrichment analysis was performed in MEME. All motifs were significantly enriched in the corresponding datasets (e-value < 0.05).

(D) Manually identified SMAD motifs from (C). The nucleotide sequence shows experimentally determined SMAD binding sequence context and the corresponding studies are indicated above the sequence bar. CAGA-box is highlighted in yellow. Manually detected SMAD binding motifs are labelled below the sequence bar. All motifs that contain an uninterrupted AGAC sequence are marked with black bars.

(E) Enrichment analysis of the CCAGACA SMAD binding motif under SMAD3 binding sites unique to MDA, unique to HCC and those shared between BTICs. Analysis was performed in HOMER (See Supplemental Experimental Procedures for details).

Figure S2. SMAD3 Binding Patterns in the Gene Distal Space. Related to Figure 2.

(A) Sketch of the analysis approach used to define SMAD3-bound enhancers by proximity association. All SMAD3 peaks falling within 20 kb up- and downstream of the gene boundaries were annotated to that gene.

(B) Genome-wide analysis showing the enrichment of each of the four binding modes of SMAD3-bound distal enhancers on TGFβ-dependent genes. Gene expression data from 6h and 24h time-points were used. Enrichment was calculated over SMAD3 binding distribution in the TGFβ-independent, background gene set. Note that the common binding mode (3) does not exclude SMAD3 binding sites that are annotated to the same genes but occur on different sites in the two BTICs.

(C) Schematic of the nearest gene association approach. Each SMAD3 peak was associated with the nearest gene, excluding SMAD3 peaks in the gene-proximal space. For each TGFβ-dependent gene the number of context-specific SMAD3 binding sites (red and blue) and shared binding sites (grey) were then calculated and represented as a composite profile.

(D) and (E) Analysis of the SMAD3-bound enhancer profiles associated with context-specific TGFβ-dependent genes (performed as outlined in C). TGFβ-dependent genes 24h post-TGFβ stimulation were used. Genes are aligned along the x-axis, and grouped into distinct categories based on their SMAD3 composite profiles. SMAD3 binding modes are indicated below the plot in grey. Light blue box marks genes with mutually exclusive SMAD3 binding patterns, and yellow those with predominantly similar or identical SMAD3 binding patterns in both BTICs.

(F) Sketch of the potential limitations of the nearest gene association approach, where peaks that functionally represent the same regulatory element - a "superenhancer" (Whyte et al., 2013) might be annotated to different genes.

Figure S3. MDA-MB-231 and HCC-1954 BTICs Harbour Global Epigenetic Differences. Related to Figure 3.

(A) Genomic annotation of the profiled chromatin factors. Peak annotation was performed in annovar and promoter overlap was computed using the validated promoter list from Weber et al., 2007.

(B) Density plots showing the pair-wise comparison of the intensity of detected peaks in MDA-MB-231 and HCC-1954 BTICs. Data are represented as the $\log_2$ of the reads per total.

(C) Average binding profiles of each of the factors around SMAD3 binding sites, in a genomic window of 1 kb.

(D) Maps showing the occupancy of Pol II, H3K27ac, H3K4me3, H3K27me3 and DNA methylation around genomic SMAD3 binding sites, in the windows of 10 kb. SMAD3 peaks are grouped into three categories (top to bottom): peaks shared between both BTICs, peaks unique MDA-MB-231 BTICs and peaks unique to HCC-1954 BTICs.

(E) Gene set enrichment analysis showing the under-representation of genes with differential levels of Pol II and epigenetic modifications between BTICs within the MDA-unique (left) and HCC-unique (right) gene sets. The significance of under-representation is shown as a p-value on a bi-symmetrical x-axis, the left and the right sides of the axis corresponding to the depletion of genes with more binding of the corresponding mark in HCC-1954 (blue) and MDA-MB-231 (red), respectively. p-value cut-offs were set at 0.05 ($-\log_{10}(0.05) = 1.30$) (dashed lines). Analysis was performed as in Figure 3A. See Supplemental Experimental Procedures for details.

Figure S4. TGFβ-dependent Regulation of *LBH* and *VGLL3* Depends on SMAD2/3 and DNA Methylation. Related to Figure 5.

(A), (B), (C) and (D) RT-qPCRs measuring the levels of *LBH* (A), *VGLL3* (B), *SMAD2* (C) and *SMAD3* (D) transcripts upon siRNA-mediated knock-down of *SMAD2* alone, *SMAD3* alone, or *SMAD2* and *SMAD3* in combination. The cells were transfected upon seeding, allowed to form mammospheres for 7 days, and then treated with TGFβ for 6h (6h time point was chosen in order to assess the effects of SMAD2/3 depletion on *LBH* and *VGLL3* simultaneously, as *LBH* is induced by TGFβ at 3h of treatment and *VGLL3* at 6h of treatment). Gene expression was normalised to the housekeeping (*RBM22*) transcript levels. Mean of three biological replicates with SD is shown, asterisks indicate significant differences, ns = not significant (two-way ANOVA).

(E) RT-qPCR showing the expression of *VGLL3* transcript upon 5-aza-dC and TGFβ treatment in MDA-MB-231 BTICs. The samples used are identical to those used in the experiments on Figure 5D. The data were normalised to the housekeeping *PSMC4* transcript levels, and presented as mean ± standard deviation (SD), asterisks indicate significant differences (two-tailed t-test). The purpose of this experiment was to ensure that the diminished *VGLL3* induction in MDA-MB-231 upon 5-aza-dC treatment is not a result of a technical problem or 5-aza-dC affecting the expression of the *RBM22* housekeeper.

(F) and (G) Targeted bisufite sequencing measuring DNA methylation levels across *LBH* (F) and *VGLL3* (G) promoters, in MDA-MB-231 (top) and HCC-1954 (bottom) BTICs, upon treatment of cells with 5-aza-dC. Asterisks indicate significant differences ($p<0.1$) as determined by FDR-corrected logistic regression test.

Figure S5. *LBH* and *VGLL3* Transcript Levels Are Depleted by siRNA-mediated Knock-down and Change with the Developmental Status. Related to Figure 6.

(A) and (B) RT-qPCRs measuring *LBH* and *VGLL3* transcript levels, respectively, upon siRNA-mediated knockdown of *LBH* and *VGLL3*. The cells were treated with TGFβ and transfected with siRNAs at the moment of seeding, and then allowed to form mammospheres for 7 days. Gene expression was normalised to the housekeeping (*PSMC4*) transcript levels. Mean of three replicates with SD is shown, asterisks indicate significant differences (one-way ANOVA).

(C) *LBH* transcript expression in different cell compartments of the mouse mammary gland. Basal compartment, luminal progenitors (LP) and differentiated luminal cells (DL) are shown. Significance was determined by a linear model (ANOVA) comparing LP and DL expression to the basal group. Data from Shehata et al., 2012.

(D) Genome browser screenshots showing transcript expression (mRNA-seq) signals over the *LBH* locus in distinct cell types of the normal mammary gland and in the variant human mammary epithelial (vHMEC) cells. Publicly available data were obtained from the Roadmap Epigenomics project (Kundaje et al., 2015). All tracks are presented on the same scale for all the samples (not shown for simplicity).

Supplemental Tables

Table S1. Dynamics of TGFβ-mediated Gene Expression in BTICs. Related to Figure 1.

An Excel file providing the lists of TGFβ-dependent genes at four different time points (1h, 3h, 6h and 24h) upon pathway induction, in MDA-MB-231 and HCC-1954 BTICs.

Table S2. SMAD3-high, Open Chromatin-high and DNA Hypo-methylation Gene Sets. Related to Figure 3.

An Excel file providing the lists of genes in the SMAD3-high, open chromatin-high and DNA hypo-methylation sets for each BTIC type.

Supplemental Experimental Procedures

Cell Propagation in Adherent Cultures

Breast cancer cell lines were first grown as adherent cultures for the purpose of propagation. MDA-MB-231 was grown in DMEM supplemented with 10% fetal bovine serum (FBS) and 100 U/ml penicillin-streptomycin (Life Technologies), and HCC-1954 was grown in RPMI supplemented with 10% FBS and 100 U/ml penicillin-streptomycin. When reaching around 80% confluence, the cells were washed with PBS, then collected and singularised using 0.05% trypsin (Invitrogen). Trypsin was inactivated with the supplemented DMEM or RPMI media and the cells were collected and washed twice with PBS. After washing and for the purpose of seeding mammosphere cultures, the cells were re-suspended in an appropriate volume of DMEM-F12 media supplemented with 0.1 x B27, 20 ng/μl fibroblast growth factor (FGF), 20 ng/μl epidermal growth factor (EGF) and 100 U/ml penicillin-streptomycin.

Mammosphere Cultures

Mammosphere cultures were seeded at 1×10^5 cells/ml density. Mammospheres were grown in DMEM-F12 media supplemented with 0.1 x B27, 20 ng/μl FGF, 20 ng/μl EGF and 100 U/ml penicillin-streptomycin in ultra-low attachment plates (Corning).

TGFβ Pathway Manipulation

To stimulate the TGFβ pathway recombinant TGFβ1 protein (R&D Systems) was used at a final concentration of 0.1 nM (of the protein dimer). Duration of the stimulus is indicated in each experiment.

5-Aza-2'-deoxycytidine Treatment

To reduce global levels of DNA methylation, attached cells prior to mammosphere seeding were treated with 5-aza-2'-deoxycytidine (Sigma-Aldrich) at 1 μM final concentration during two

consecutive passages. The third spike-in of 5-aza-2'-deoxycytidine was added immediately after seeding mammospheres. The spheres were grown for 7 days and then treated with TGFβ for 24h. Mammospheres were collected, washed twice with PBS and then each sample was split in two, one half for DNA extraction and one half for RNA extraction. For RNA extraction, pellets were lysed in Qiazol and RNA was extracted using miRNeasy kit (Qiagen). DNA was extracted using phenol-chloroform extraction and ethanol precipitation.

siRNA-mediated Knock-down Experiments

To knock-down *LBH*, *VGLL3*, *SMAD2* and *SMAD3* in mammospheres, for each gene pools of 4 targeting siRNAs were used (GE Healthcare) at 25 nM final concentration. Non-targeting siRNA (GE Healthcare, D-001810-01-20) was used as a control. The cells were transfected immediately after seeding mammospheres at 1×10^5 cells/ml density and Dharmafect I (GE Healthcare) was used as the transfection reagent, according to the manufacturer's protocol. DMEM-F12 medium with 0.1 x B27, 20 ng/μl FGF, 20 ng/μl EGF but without any antibiotics was used for the whole duration of the experiment. In experiments with *LBH* and *VGLL3* knock-downs, TGFβ was added to the cells at 0.1 nM final concentration two hours after transfection, and mammospheres were then allowed to form for 7 days. In *SMAD2* and *SMAD3* knock-down experiments, the mammospheres were allowed to form for 7 days, and TGFβ pathway was then activated for 6h by addition of exogenous TGFβ at 0.1 nM final concentration. RNA was extracted using miRNeasy kit (Qiagen).

MS-IC and CFC Assays

To assess modulation of self-renewal and proliferation capacity induced by *LBH* and *VGLL3* knock-downs and TGFβ treatment, mammosphere initiating cell (MS-IC) assays and colony forming cell (CFC) assays were performed in parallel. siRNA transfections were performed as outlined above.

Seven days old mammospheres were span down at 1300 g and washed with PBS once. To obtain single cells, 1 ml of 0.05% trypsin was added to mammosphere pellets followed by incubation at 37°C for 2 minutes. Cells were then singularized by gentle pipetting. Trypsin was inactivated with 1 μl of 1000 x Trypsin Inhibitor (Roche) and diluted in 10 ml of PBS. Cells were centrifuged and the pellet was resuspended in an appropriate volume of DMEM-F12 media supplemented with 0.1x B27, 20 ng/μl FGF, 20 ng/μl EGF and 100 U/ml penicillin-streptomycin, to yield required dilutions of cells (1:2 for MDA-MB-231 and 1:4 for HCC-1954). Second generation spheres were seeded in ultra low attachment 96-well plates (Corning).

To image and count the number of mammospheres, a colorimetric assay was performed, where live cells were labeled on the 6th day from seeding with 1X tetrazolium dye 3-(4,5-dimethylthiazol-2-yl)-2,5-diphenyltetrazolium bromide (MTT), allowed to incorporate the dye over night, and imaged the following day on the Gel Count scanner (Oxford Optronix). Spheres were counted using automated Gel Count software. Data was analyzed with Prism 6.0 (GraphPad Software) and statistical significance was determined by ANOVA.

CFC assay was performed in parallel with second generation mammosphere assay. Singularized cells were seeded in 6 cm round collagen coated dishes (Fisher Scientific) at cell line specific densities (1:120 for MDA-MB-231 and 1:80 for HCC-1954), in 3 ml DMEM-F12 media supplemented with 5% FBS, 20 ng/μl FGF and 100 U/ml penicillin-streptomycin. The growth of colonies was monitored daily, and when reaching the appropriate density, all plates were washed twice with PBS, then fixed with methanol:acetone (1:1) for 30 s. The plates were then stained with 1:10 diluted Giemsa dye for 30 min, washed twice with PBS, allowed to air dry, and imaged on the Gel Count scanner (Oxford Optronix).

Targeted Bisulfite Sequencing

200 ng of DNA from 12 samples (3 biological replicates of each of the following conditions: MDA-MB-231 Control, MDA-MB-231 treated with 5-aza-dC, HCC-1954 Control and HCC-1954 treated with 5-aza-dC), were bisulfite converted and then column-purified using DNA methylation Gold kit (Zymo Research). This converted DNA template was used to amplify 10 regions of interest (spanning *LBH* and *VGLL3* promoters) using PfuTurbo Cx Hotstart DNA Polymerase (Agilent Technologies) (50 μl reaction: 5 μl 10X Pfu Turbo Cx Buffer, 1.25 μl dNTPs (10 mM each), 5 μl F+R primer mix (2.5

μM each), 1 μl PfuTurbo Hotstart Cx Polymerase, water up to 50 μl) in 40 PCR cycles. Primers were designed in Bisulfite Primer Seeker (Zymo Research), and for each primer pair the annealing temperatures were first optimised by testing temperature gradients (data not shown). The following 10 PCR primer pairs were used (when used across 12 samples giving a total of 120 PCR reactions):

	Region	Forward Primer	Reverse Primer	T(°C)
1.	LBH_1	TTATAGGGGYGTGTGTTAG TTTGTTTTAGG	AATTCACACRTAACCCCTA ACTCCCCC	59.8
2.	LBH_2	GAGYGTTGAAGTTATTTAT GATTTTGG	AATATATAAACATAAAATC CTAAAACAAACTAACACA C	54.2
3.	LBH_3	TTGGTGAYGTTATTTTAGG AGTGGG	AATATATAAACATAAAATC CTAAAACAAACTAACACA C	54.2
4.	LBH_4	AGGGGTTAYGTGTGAATTT TTTTTAATG	CCAATCCCRACCCCCACCA ATATAC	55.5
5.	LBH_5	GGTGGGGGGAGGGGGTGT TGAGAATATTTAGATAAA G	CACCCCCCCRAAACTCTAC AAACCCTAC	63.4
6.	LBH_6	GGAGAAGAYGTGGGAGTT AAGGATGGGG	CCCTCTAAAACRTTTATTC CCCATACTAAACCTCTCTC	63.1
7.	VGLL3_1	GTYGGTTGATAGTAGGTTG TGGGGTAGGTTG	CAAAAAACATCCRAAAAA ACTAAAAATAAAAATACC	55.5
8.	VGLL3_2	GGTAGGTTGTYGTTGTTAT GGGGTTGGGTAGATATTG	CAAAAAACATCCRAAAAA ACTAAAAATAAAAATACC	55.5
9.	VGLL3_3	GTTYGGTGATTTATTTGTT GGTTAGGTTGGGG	TAAACCCCCRCTAATTACC AATCCCTCCC	61.5
10.	VGLL3_4	GGGGTYGGGAGGGATTGG TAATTAG	TACAAACCRAAACTAAACT CCCCAC	56.9

PCR products were purified using 2X volume of solid phase reversible immobilisation (SPRI) beads (Illumina), two 80% ethanol washes and eluted in 25 μl of 10mM Tris-HCl, pH 8.5. Amplicons were then end-repaired and A-tailed in 30 μl reactions (1 μl of Klenow 5'->3' exo- (NEB), 3 μl of 10X NEB2 buffer, 1 μl of the dNTP solution (1mM dCTP, 1mM dGTP, 1mM dTTP and 10 mM ATP (in excess for A-tailing))), by incubation at 30°C for 20 min and 37°C for 20 min. Amplicons were purified from this reaction by addition of 2X volume of SPRI as above however at the last step the beads were retained in the 20 μl 10mM Tris-HCl, pH 8.5 elute and carried over through the subsequent reaction.

Amplicons were then ligated to barcoded methylated DNA adapters (TruSeq LT, Illumina) by adding 2 μl of 1:20 diluted TruSeq Illumina adapters, and then the master mix containing 1 μl T4 DNA Ligase (400,000 U/ml) (NEB), 3 μl T4 DNA Ligase buffer (NEB) and 4 μl nuclease-free water, giving the total 30 μl ligation reaction per sample. Barcoding was performed in such way that all amplicons from the same sample harbour one, same barcode. Ligation was carried out over night at 16°C and the following day it was inactivated by incubation at 65°C for 20 min. Ligation products were purified by

addition of double volume of PEG-NaCl solution (20% w/v PEG 8000, 2.5M NaCl) to the bead-containing ligation reaction, two 80% ethanol washes and elution in 10 µl of 10mM Tris-HCl, pH 8.5.

After purifying these 120 barcoded amplicons the quantity of each one was assessed using qPCR (KAPA Biosystems). These measurements were used to normalize sample amounts for pooling: amplicons representing the same region (e.g. LBH_1) from 12 different samples were pooled in equimolar ratios, giving rise to 10 pools, each representing a different genomic region. These 10 pools were then combined (without normalizing their amounts across each other, as some of them had very low yields), and purified with 2X volume of SPRI, two 80% ethanol washes and elution in 100 µl of 10mM Tris-HCl, pH 8.5, in order to bring down the volume of the solution.

This pooled library was then amplified with PfuTurbo Cx Hotstart DNA Polymerase (Agilent Technologies) in 14 PCR cycles (reaction conditions identical as above apart from the reaction volume which was now 200 µl). The amplified library was then purified with two consecutive SPRI purifications, one with 1.2X bead-to-sample volume ratio, and another with 1.5X bead-to-sample volume ratio, and both using two 80% ethanol washes and elution in 10mM Tris-HCl, pH 8.5. A small aliquot of the final library was taken and diluted for quality controls (1:10 dilution for Bioanalyser HS and 1:4,000 for qPCR (KAPA Biosystems)).

Sequencing was performed on MiSeq (Illumina) using 150 bp paired end sequencing (CRUK CI Genomics Core). Quality control and trimming was performed with FastQC and trim galore (Andrews, 2010) and reads were aligned to the Human Genome Build 37 (hg19) using Bismark (Krueger and Andrews, 2011). Methylation of individual CpG sites was called using Bismark too. Downstream data analysis was performed in R and detection of differentially methylated CpGs was done fitting a logistic regression model to each CpG and correcting the p-values using FDR with a threshold of 0.1.

Reverse Transcription Quantitative PCR (RT-qPCR)

To generate cDNA, 100-400 ng of RNA per sample was mixed with 1µl of 50 µM custom made Oligo-$(dT)_{16}$ (5'-$d(T)_{16}$VN-3', V= dA or dG or dC; N = dA or dG or dC or dT), and denatured at 65°C for 5 min. The samples were snap cooled on ice and reverse transcription was performed with Transcriptor First Strand cDNA Synthesis Kit (Roche), as recommended by the manufacturer. The samples were incubated at 25°C for 10 min, 55°C for 30 min and 85°C for 5 min.

Generated cDNAs were diluted with nuclease-free water in 1:10 ratio, and qPCR was performed using the TaqMan chemistry. Gene-specific TaqMan probes and TaqMan Fast Universal PCR Master Mix (Applied Biosystems) were used, as recommended by the manufacturer. The plates were run on Applied Biosystems 7900HT Fast Real-Time PCR system, with the fast cycling conditions (20s of denaturation at 95°C, followed by 40 cycles of 95°C for 1s and 60°C for 20s). Data were analyzed in SDS 2.4 software (Applied Biosystems), Excel (Microsoft) and Prism 6.0 (GraphPad Software). Statistical significance was determined using ANOVA.

Gene Expression Profiling using Illumna HumanHT-12 BeadChips

Gene expression analysis was performed on HumanHT-12 Expression BeadChips from Illumina, using 200 ng of RNA as a starting material. For each condition, biological triplicates were used.

Gene Expression Data Analysis

Gene expression data were analyzed with the beadarray package (Dunning et al., 2007). First, quality assessment was performed. Then, spatial artifacts were removed using BASH (Cairns et al., 2008), and probes were summarized and quantile normalized. Probe re-annotation was done using the IlluminaHumanv4.db package (Barbosa-Morais et al., 2010) and only probes that were a perfect match to their target were kept. A linear model comparing the expression of TGFβ-treated cells vs non-treated cells within each replicate was fit using the limma package (Smyth, 2005) and lists of differentially expressed genes for each cell line were obtained using a threshold of 0.1 FDR.

Chromatin Immunoprecipitation and Sequencing

Roughly 7×10^7 cells per condition at 1×10^5 cells/ml density were seeded as mammosphere cultures for SMAD3 ChIPs. For all other ChIPs (Pol II and histone modifications) 2×10^7 cells were used per condition. Due to the large scale of the experiment, cells were grown in 500 ml volume units in low attachment spinner flasks (Corning) that provide constant mixing thereby preventing aggregation of cells. Mammospheres were allowed to form for 7 days. For SMAD3 experiments only the cells were treated with TGFβ at 0.1 nM final concentration for 3h. For all other experiment untreated mammospheres were used.

Mammospheres were aliquoted in 50 ml falcon tubes, centrifuged and washed 3 x with PBS. Following the final wash, mammospheres were crosslinked in 30 ml of 1.66 mM Di(N-succinimidyl) glutarate (DSG) solution (dissolved in PBS) per condition, and incubated for 45 min at room temperature on the turning wheels. Mammospheres were washed 3 x with PBS and resuspended in 20 ml of PBS. 2 ml of freshly prepared formaldehyde solution (50 mM Hepes-KOH pH 7.5, 100 mM NaCl, 1 mM EDTA, 0.5 mM EGTA, 11% (v/v) formaldehyde) were then added to each sample and the samples were incubated for 30 min at room temperature with tumbling. The reaction was then quenched with 1/10 of the volume of 1.25 M glycine, incubated at room temperature for 5 min, and crosslinked mammospheres were washed 2 times with ice-cold PBS.

To remove cytosol and extract nuclei, mammosphere pellets were washed three times for 5 min with Nuclear extraction buffer (20 mM Tris-HCl pH 8.0, 10 mM NaCl, 2 mM EDTA, 0.5% (v/v) Igepal CA630, 1x protease inhibitor cocktail (PIC, Roche)). From this point, all centrifugation steps were carried out at 4°C. The pellets were then re-suspended in 2 ml of Sonication buffer (20 mM Tris-HCl pH 7.5, 150 mM NaCl, 2mM EDTA, 1% (v/v) Igepal CA630, 0.3% (v/v) sodium dodecyl sulfate (SDS), 1x PIC) and transferred to 5 ml Covaris tubes (LGC Genomics). Chromatin shearing was performed on Covaris S-220, for the total duration of 8-9 min.

To remove debris, samples were transferred to 15 ml Falcon tubes and centrifuged at maximum speed (4,000 g) at 4°C for 20 min. The supernatants were transferred to new tubes containing 1 volume (2 ml) of the ChIP dilution buffer (20 mM Tris-HCl pH 8.0, 150 mM NaCl, 2 mM EDTA, 1% (v/v) Triton X-100, 1x PIC). At this point 1% of each sample volume was set aside to serve as an input, and stored at -20°C until further processing.

To each sample, 45 μl of 10 % bovine serum albumin (BSA), 0.5 ml of the pre-blocked Protein G Agarose beads (Santa Cruiz, 60 μl of beads of stock per condition, washed twice and then blocked for several hours in 500 μl ChIP buffer containing 0.1 % (w/v) BSA) and 10 μg of the corresponding antibody were added. The following antibodies were used: anti-SMAD3 (ab28379), anti-RNA Pol II (ab5408), anti-H3K4me3 (Millipore, 05-1339), anti-H3K27me3 (Millipore, 07-449) and anti-H3K27ac (ab4729). Immunoprecipitation was carried over night at 4°C with tumbling.

The beads were then span down at 40 g for 5 min at 4°C, the supernatant containing the unbound chromatin fraction was removed, the beads were re-suspended in 1 ml of ChIP buffer (20 mM Tris-HCl pH 8.0, 150 mM NaCl, 2 mM EDTA, 1% (v/v) Triton X-100, 0.15% (w/v) SDS, 1x PIC) and transferred to DNA LoBind tubes (Eppendorf). This was followed by one wash with Wash buffer 2 (20 mM Tris-HCl pH 8.0, 500 mM NaCl, 2 mM EDTA, 1% (v/v) Triton X-100, 0.1 % (w/v) SDS, 1x PIC), one wash with Wash buffer 3 (20 mM Tris-HCl pH 8.0, 250 mM LiCl, 2 mM EDTA, 0.5 % (v/v) Igepal CA630, 0.5 % (w/v) sodium deoxycholate, 1x PIC) and two washes with TE buffer (10 mM Tris-HCl pH 7.4, 1 mM EDTA), all at 4°C.

After the final wash the beads were re-suspended in 250 μl of freshly prepared, pre-warmed (37°C) Elution buffer (100 mM $NaHCO_3$, 1% (w/v) SDS) and incubated with shaking at room temperature for

15 min. The beads were span down at 1,000 rpm and the supernatant fraction (elute) was saved in a new DNA LoBind tube. Another 250 μl of pre-warmed (37°C) Elution buffer were then added to the beads and the second elution was performed with shaking at room temperature for 15 min. The beads were then span down, and the supernatant (second elute) was combined with the first one. 500 μl of the combined elute was then also span down to eliminate any remaining agarose beads, and the supernatant (480 μl) was transferred to a new DNA LoBind tube.

To reverse the crosslink between the DNA and proteins, 20 μl of 5 M NaCl was added to each 480 μl elute (for immunoprecipitated samples) and to input samples which were first set to 480 μl by adding water. These reactions were incubated at 65°C over night. The following day, to digest proteins, 20 μl of 1M Tris-HCl pH 8.0, 10 μl of 0.5 M EDTA and 5 μl of 10 mg/ml Proteinase K were added to each 500 μl sample, and the mixtures were incubated at 55°C for 1h. DNA was purified from this reaction by phenol-chloroform extraction and overnight ethanol precipitation (with addition of glycogen or glycoblue as carriers) at -20°C.

Libraries for Illumina sequencing were prepared with TruSeq LT ChIP kit, as recommended by the manufacturer. Size selection of the DNA libraries was performed on 2% TAE agarose gels, selecting for fragments in the size range 200-600 bp. Sequencing was performed on HiSeq 2000 (Illumina), with 40 bp single end reads. For each of the marks, multiplexed pools of 12 samples were sequenced on multiple lanes, to meet the following coverage criteria: SMAD3 – 30 x 10^6 reads per sample, Pol II - 60 x 10^6 reads per sample, H3K27ac – 30 x 10^6 reads per sample, H3K4me3 – 30 x 10^6 reads per sample, H3K27me3 – 45 x 10^6 reads per sample.

ChIP-seq Data Analysis

Briefly, bases were called and the general quality of the sequencing run was assessed by the FastQC pipeline (Andrews, 2010). The reads were then filtered based on sequencing quality and aligned to the Human Genome Build 37 (hg19) using BWA (Li and Durbin, 2009).

The peaks were called using MACS (Zhang et al., 2008) for SMAD3, and SICER (Zang et al., 2009) for all other factors profiled. Only peaks common in both biological replicates were kept.

Read counting in specific genomic features was performed with the R package Rsubread (Liao et al., 2013).

Motif analyses were performed using MEME (Machanick and Bailey, 2011) and HOMER (Heinz et al., 2010). HOMER version 4.7 was run to find the distribution of the motif CCAGACA around MDA-MB-231 unique SMAD peaks, HCC-1954 unique SMAD peaks and SMAD peaks common to both cell lines. The command annotate peaks.pl was run with a bin size of 25 and a maximum distance of +/-2500 bases around the peak centre.

DNA Methylation Profiling by MBD-sequencing

DNA methylation profiles were obtained from untreated, 7 days old MDA-MB-231 and HCC-1954 mammospheres. Methylated DNA was enriched using recombinant methyl binding domain (MBD2b/MBD3L1) protein complex as part of MethylCollector Ultra kit (Active Motif), following manufacturer's recommendations. Briefly, 1 μg of sonicated DNA was used in each MBD pulldown reaction, with high-salt buffer (AM7 buffer) in order to increase the stringency of binding conditions. Nickel-coated magnetic beads were used to precipitate His-tagged MBD complex bound to methylated DNA fragments. This pulldown reaction was incubated for 2 hours at 4°C on a turning wheel. This was followed by precipitation of beads on a magnetic stand and 4 rounds of washing with AM7 buffer. After the final wash, all residual AM7 buffer was removed. DNA was eluted from the beads and MBD complex in elution buffer containing proteinase K, for 45 min at 50°C in a thermomixer, with intermittent vortexing. DNA was further purified by phenol-chloroform extraction and ethanol precipitation. Libraries for Illumina sequencing were prepared with TruSeq LT kit (Illumina). Size selection of the DNA libraries was performed on 2% TAE agarose gels, selecting for fragments in the size range 200-600 bp. Sequencing was performed on HiSeq 2000 (Illumina), with 40 bp single end reads. Multiplexed pools of 12 samples were sequenced on a single lane to yield roughly 16 x 10^6 reads per sample.

MBD-seq Data Analysis

Briefly, bases were called and the general quality of the sequencing run was assessed by the FastQC pipeline (Andrews, 2010). The reads were then filtered based on sequencing quality and aligned to the Human Genome Build 37 (hg19) using Burrows-Wheeler Aligner (BWA) (Li and Durbin, 2009). Post-alignment, Bi-asymmetric-Laplace model (BALM) was used to call methylation peaks (Lan et al., 2011), and (MeD)IP-seq data analysis (MEDIPS) (Lienhard et al., 2014) was used for quantitative analysis, whereby the data was normalised to the CG content. Reads per kilobase per million reads (rpkm) were also calculated. Genomic regions of interest were defined based on published annotations of genomic elements (Wang et al., 2010), promoter annotation was obtained from the validated list in (Weber et al., 2007) and lifting over to hg19 was performed as previously described (Hinrichs et al., 2006).

Integration of ChIP-seq, MBD-seq and Gene Expression Data

The integration of binding sites and differentially expressed genes was performed using two different approaches. The first approach (used for Figures 1 and 2) was to simply find if a SMAD3 peak overlapped with the gene position. The gene positions were obtained from The UCSC Genome Browser Database (assembly GRCh37) and 1,500 bases upstream of the transcription start sites were added. Second approach (used for Figures 3 and S3E) was to test for differential binding between the two cell lines, in the aforementioned gene-unit spanning 1500 bases upstream of the transcription start sites to gene ends, except for DNA Methylation, where a window of -/+ 1500 bases around the TSS was taken. This was conducted using edgeR (Robinson et al., 2010) and setting the following thresholds: FDR < 0.1, absolute log Fold Change >= 0.5 and log Count per Million reads >=4.

For the analysis of distal SMAD3-bound elements, two approaches were used. In the first one (Figures S2A and S2B), peaks were annotated to a gene if they fell within a window spanning 20 kb from gene boundaries. In the second approach (Figures S2C, S2D, S2E and S2F), each SMAD3 peak was annotated to the nearest gene. The rest of the analysis was performed as for the gene-proximal SMAD3 peaks.

Calculating Enrichment of SMAD3 Binding Modes on TGFβ-dependent Genes

Each gene in the genome was annotated into one of the four defined SMAD3 binding modes, as well as into one of the four gene groups (three TGFβ-dependent gene sets: MDA-unique, HCC-unique and shared, and one TGFβ-independent gene set, containing all the other genes in the genome). Annotations were then compared to derive frequencies of each binding mode within each of the gene groups. Enrichment scores were calculated by normalising mode frequencies from TGFβ-dependent gene sets over frequencies in the TGFβ-independent gene set.

Integration of Differential Binding and Gene Set Enrichment Analyses

Enrichment of differentially bound genes in the lists of differentially expressed genes was done considering all the genes annotated in the Illumina array as universe, and the list of uniquely differentially expressed genes between TGFβ and Control in each cell line as the signature to test. Enrichment was computed using the GOSeq package (Young et al., 2010) to take into account biases due to different gene lengths.

Analysis of Gene Expression Data from Primary Tissues

Classification of Claudin^{low} samples was done following the classifier in Prat et al., 2010. A linear model was fitted to test if expression levels were different amongst groups. If the number of comparisons was larger than two, simultaneous testing was used following the R package multcomp (Hothorn et al., 2008).

Kaplan-Meier estimates and log-rank tests were computed using the R survival package (Therneau, 2014).

Supplemental References

Andrews, S. (2010). FastQC: A quality control tool for high throughput sequence data.

Barbosa-Morais, N.L., Dunning, M.J., Samarajiwa, S.A., Darot, J.F., Ritchie, M.E., Lynch, A.G., and Tavare, S. (2010). A re-annotation pipeline for Illumina BeadArrays: improving the interpretation of gene expression data. Nucleic Acids Res *38*, e17.

Cairns, J.M., Dunning, M.J., Ritchie, M.E., Russell, R., and Lynch, A.G. (2008). BASH: a tool for managing BeadArray spatial artefacts. Bioinformatics *24*, 2921-2922.

Dunning, M.J., Smith, M.L., Ritchie, M.E., and Tavare, S. (2007). beadarray: R classes and methods for Illumina bead-based data. Bioinformatics *23*, 2183-2184.

Heinz, S., Benner, C., Spann, N., Bertolino, E., Lin, Y.C., Laslo, P., Cheng, J.X., Murre, C., Singh, H., and Glass, C.K. (2010). Simple combinations of lineage-determining transcription factors prime cis-regulatory elements required for macrophage and B cell identities. Mol Cell *38*, 576-589.

Hinrichs, A.S., Karolchik, D., Baertsch, R., Barber, G.P., Bejerano, G., Clawson, H., Diekhans, M., Furey, T.S., Harte, R.A., Hsu, F.*, et al.* (2006). The UCSC Genome Browser Database: update 2006. Nucleic Acids Res *34*, D590-598.

Hothorn, T., Bretz, F., and Westfall, P. (2008). Simultaneous inference in general parametric models. Biom J *50*, 346-363.

Krueger, F., and Andrews, S.R. (2011). Bismark: a flexible aligner and methylation caller for Bisulfite-Seq applications. Bioinformatics *27*, 1571-1572.

Kundaje, A., Meuleman, W., Ernst, J., Bilenky, M., Yen, A., Heravi-Moussavi, A., Kheradpour, P., Zhang, Z., Wang, J., Ziller, M.J.*, et al.* (2015). Integrative analysis of 111 reference human epigenomes. Nature *518*, 317-330.

Lan, X., Adams, C., Landers, M., Dudas, M., Krissinger, D., Marnellos, G., Bonneville, R., Xu, M., Wang, J., Huang, T.H.*, et al.* (2011). High resolution detection and analysis of CpG dinucleotides methylation using MBD-Seq technology. PLoS One *6*, e22226.

Li, H., and Durbin, R. (2009). Fast and accurate short read alignment with Burrows-Wheeler transform. Bioinformatics *25*, 1754-1760.

Liao, Y., Smyth, G.K., and Shi, W. (2013). The Subread aligner: fast, accurate and scalable read mapping by seed-and-vote. Nucleic Acids Res *41*, e108.

Lienhard, M., Grimm, C., Morkel, M., Herwig, R., and Chavez, L. (2014). MEDIPS: genome-wide differential coverage analysis of sequencing data derived from DNA enrichment experiments. Bioinformatics *30*, 284-286.

Machanick, P., and Bailey, T.L. (2011). MEME-ChIP: motif analysis of large DNA datasets. Bioinformatics *27*, 1696-1697.

Prat, A., Parker, J.S., Karginova, O., Fan, C., Livasy, C., Herschkowitz, J.I., He, X., and Perou, C.M. (2010). Phenotypic and molecular characterization of the claudin-low intrinsic subtype of breast cancer. Breast Cancer Res *12*, R68.

Robinson, M.D., McCarthy, D.J., and Smyth, G.K. (2010). edgeR: a Bioconductor package for differential expression analysis of digital gene expression data. Bioinformatics *26*, 139-140.

Smyth, G.K. (2005). Limma: linear models for microarray data. In Bioinformatics and computational biology solutions using R and Bioconductor, V.C. R. Gentleman, S. Dudoit, R. Irizarry, W. Huber, ed. (New York, Springer Science+Business Media), pp. 397-420.

Therneau, T. (2014). A Package for Survival Analysis in S.

Wang, K., Li, M., and Hakonarson, H. (2010). ANNOVAR: functional annotation of genetic variants from high-throughput sequencing data. Nucleic Acids Res *38*, e164.

Weber, M., Hellmann, I., Stadler, M.B., Ramos, L., Paabo, S., Rebhan, M., and Schubeler, D. (2007). Distribution, silencing potential and evolutionary impact of promoter DNA methylation in the human genome. Nat Genet *39*, 457-466.

Young, M.D., Wakefield, M.J., Smyth, G.K., and Oshlack, A. (2010). Gene ontology analysis for RNA-seq: accounting for selection bias. Genome Biol *11*, R14.

Zang, C., Schones, D.E., Zeng, C., Cui, K., Zhao, K., and Peng, W. (2009). A clustering approach for identification of enriched domains from histone modification ChIP-Seq data. Bioinformatics *25*, 1952-1958.

Zhang, Y., Liu, T., Meyer, C.A., Eeckhoute, J., Johnson, D.S., Bernstein, B.E., Nusbaum, C., Myers, R.M., Brown, M., Li, W., *et al.* (2008). Model-based analysis of ChIP-Seq (MACS). Genome Biol *9*, R137.
