# Supplementary material for: Context-Specific Effects of TGF-β/SMAD3 in Cancer Are Modulated by the Epigenome
Source: Cell Rep. 2015 Dec 10;13(11):2480–90. doi: 10.1016/j.celrep.2015.11.040 (PMC4695334; doi:10.1016/j.celrep.2015.11.040)
Supplement: Document S2. Article plus Supplemental Information [file mmc4.pdf]

Cell Reports

Article

Context-Specific Effects of TGF-β/SMAD3 in Cancer Are Modulated by the Epigenome

Graphical Abstract

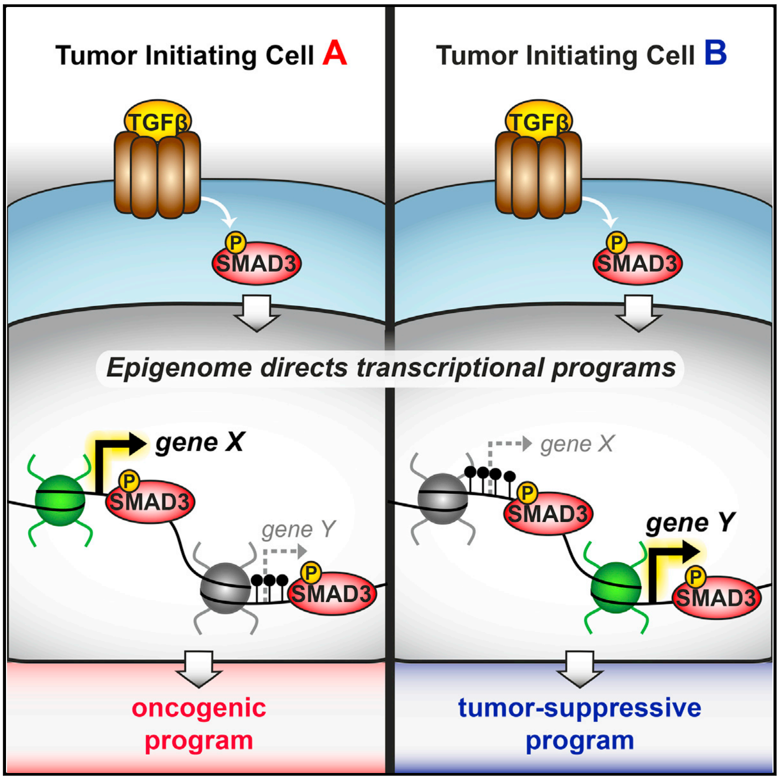

Highlights

- TGF-β has opposing effects in different breast-tumor-initiating cell (BTIC) types
- Genomic SMAD3 binding patterns are similar in BTICs with opposing responses to TGF-β
- BTIC type-specific epigenomes prime genes for regulation by TGF-β/SMAD3
- *LBH*, a type-specific TGF-β target, is essential for BTIC-promoting effects of TGF-β

Authors

Ana Tufegdzic Vidakovic, Oscar M. Rueda, Stephin J. Vervoort, ..., Paul J. Coffer, Alejandra Bruna, Carlos Caldas

Correspondence

alejandra.bruna@cruk.cam.ac.uk (A.B.), carlos.caldas@cruk.cam.ac.uk (C.C.)

In Brief

The TGF-β pathway uses transcriptional regulation through SMAD transcription factors to modulate cell-context-specific phenotypes. Tufegdzic Vidakovic et al. show that in breast-tumor-initiating cells (BTICs), type-specific DNA and histone modifications help determine whether the response to TGF-β is pro-oncogenic or tumor suppressive. These landscapes act both in synergy and independently of cell-type-specific SMAD3 binding to TGF-β target genes to modulate context-specific transcriptional regulation by TGF-β/SMAD3.

CrossMark

Tufegdzic Vidakovic et al., 2015, Cell Reports *13*, 2480–2490
December 22, 2015 ©2015 The Authors
http://dx.doi.org/10.1016/j.celrep.2015.11.040

CellPress

OPEN ACCESS
CellPress

Cell Reports
Article

Context-Specific Effects of TGF-β/SMAD3 in Cancer Are Modulated by the Epigenome

Ana Tufegdzic Vidakovic,[1,3] Oscar M. Rueda,[1,3] Stephin J. Vervoort,[2] Ankita Sati Batra,[1] Mae Akilina Goldgraben,[1] Santiago Uribe-Lewis,[1] Wendy Greenwood,[1] Paul J. Coffer,[2] Alejandra Bruna,[1,*] and Carlos Caldas[1,*]
[1]Cancer Research UK Cambridge Institute, Department of Oncology, University of Cambridge, Cambridge CB2 0RE, UK
[2]Department of Cell Biology, Center for Molecular Medicine, University Medical Center Utrecht, 3584 CX Utrecht, the Netherlands
[3]Co-first author
*Correspondence: alejandra.bruna@cruk.cam.ac.uk (A.B.), carlos.caldas@cruk.cam.ac.uk (C.C.)
http://dx.doi.org/10.1016/j.celrep.2015.11.040
This is an open access article under the CC BY license (http://creativecommons.org/licenses/by/4.0/).

SUMMARY

The transforming growth factor beta (TGF-β) signaling pathway exerts opposing effects on cancer cells, acting as either a tumor promoter or a tumor suppressor. Here, we show that these opposing effects are a result of the synergy between SMAD3, a downstream effector of TGF-β signaling, and the distinct epigenomes of breast-tumor-initiating cells (BTICs). These effects of TGF-β are associated with distinct gene expression programs, but genomic SMAD3 binding patterns are highly similar in the BTIC-promoting and BTIC-suppressing contexts. Our data show cell-type-specific patterns of DNA and histone modifications provide a modulatory layer by determining accessibility of genes to regulation by TGF-β/SMAD3. *LBH*, one such context-specific target gene, is regulated according to its DNA methylation status and is crucial for TGF-β-dependent promotion of BTICs. Overall, these results reveal that the epigenome plays a central and previously overlooked role in shaping the context-specific effects of TGF-β in cancer.

INTRODUCTION

The effects of transforming growth factor beta (TGF-β) in tissue homeostasis depend heavily on cellular context (Massagué, 2012). TGF-β has been shown to both induce proliferation and suppress cell growth, stimulate stem cell self-renewal and promote differentiation, and inhibit early and promote late malignant transformation (Gomis et al., 2006; Guasch et al., 2007; Massagué, 2008, 2012).

In breast cancer, TGF-β can either promote or inhibit tumor-initiating cells (breast TICs, or BTICs), which are responsible for cancer initiation, propagation, and metastasis (Bierie and Moses, 2009; Bruna et al., 2012; Mani et al., 2008; Scheel et al., 2011). We have previously shown these opposing effects of TGF-β depend on breast cancer subtype (Bruna et al., 2012). BTICs are activated only in Claudin^{low} breast cancer, while in all other subtypes, TGF-β inhibits BTICs. Since no mutations in TGF-β pathway genes have been associated with specific breast cancer subtypes (Cancer Genome Atlas Network, 2012), the underlying mechanism of this dichotomy is unlikely to be genetic.

TGF-β signaling is initiated by binding of TGF-β to its cognate receptor, TGFBR II, resulting in phosphorylation of the transcription factors SMAD2 and SMAD3 (Massagué et al., 2005). Upon phosphorylation, SMAD2 and SMAD3 associate with SMAD4 and translocate to the nucleus, where they partner up with additional transcription factors (TFs) to regulate target gene expression (Massagué et al., 2005). Remarkably, TGF-β universally relies on SMADs despite regulating cell-type-specific transcriptional programs (Massagué, 2012). The current model is that cell-type-specific partner TFs guide SMADs to distinct genes, thus resulting in context-specific gene regulation and specific biological effects of TGF-β (Massagué, 2012; Mullen et al., 2011; Xu et al., 2015).

Here, we mapped genome-wide SMAD3 binding patterns in BTICs that model the opposing effects of TGF-β (Bruna et al., 2012). This showed that differential SMAD3 binding does not fully account for context-specific TGF-β target gene regulation, and further experiments revealed that distinct epigenetic states are responsible. We identify transcription factor *LBH* as a prototypical TGF-β target gene regulated by differential DNA methylation and show it is essential for the BTIC-promoting activity of TGF-β. Taken together, these data reveal an important role for epigenetic determinants in regulation of the context-specific actions of TGF-β in cancer.

RESULTS

SMAD3 Binding to Gene-Proximal Regions Mediates TGF-β-Dependent Gene Expression in BTICs

Two cell lines that we previously showed represent the opposing effects of TGF-β (Bruna et al., 2012) were used as BTIC model systems in all experiments: MDA-MB-231 for BTIC promoting, and HCC-1954 for BTIC suppressing (Figure 1A). Cells were grown in suspension as mammosphere cultures to enrich for BTICs (Bruna et al., 2012; Dontu et al., 2003a, 2003b). Confirming our previous data (Bruna et al., 2012), the canonical TGF-β signaling cascade is intact and similarly activated by its ligand in both models, as shown by SMAD2 phosphorylation (Figure 1B).

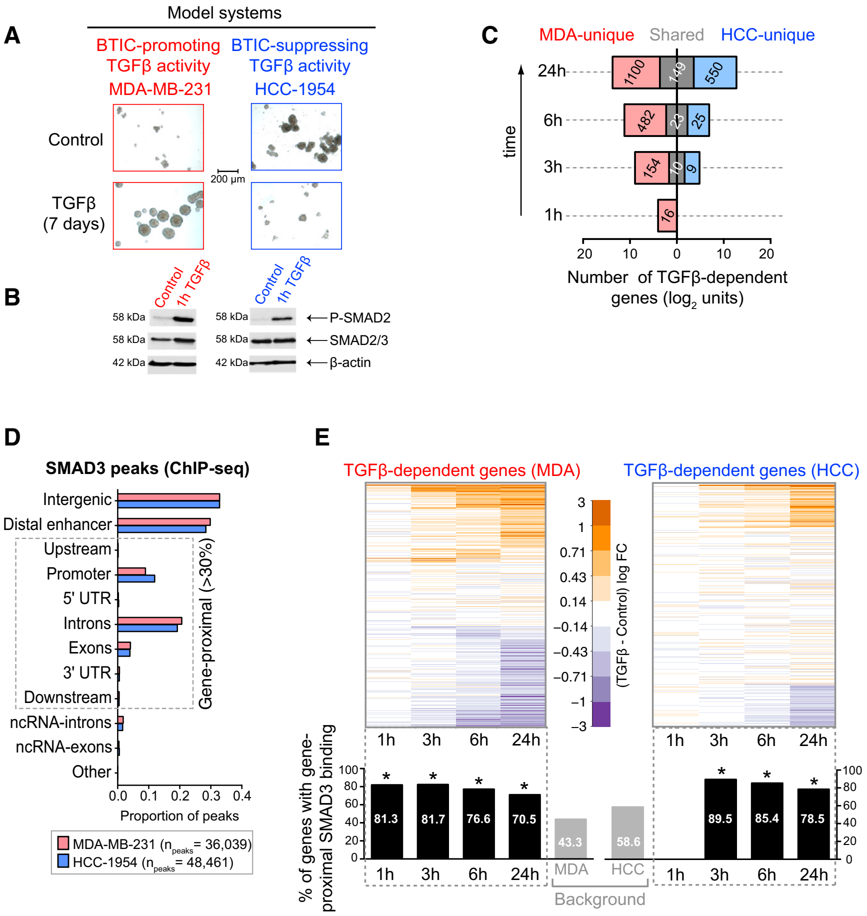

Figure 1. SMAD3 Mediates Both the BTIC-Promoting and BTIC-Suppressing Programs of TGF-β

(A) MDA-MB-231 and HCC-1954 first generation mammosphere cultures with and without addition of TGF-β. TGF-β was added to the media at the moment of cell seeding and mammospheres were allowed to form for 7 days. Note that this is not a quantitative assay.

(B) Western blot showing SMAD2 phosphorylation levels upon TGF-β pathway induction. 7-day-old mammospheres were treated with exogenous TGF-β ligand for 1 hr. Total SMAD2/3 and β-actin levels were used as loading controls.

(C) Comparison of TGF-β-dependent genes in MDA-MB-231 and HCC-1954 BTICs. MDA-MB-231 and HCC-1954 cells were grown as mammospheres for 7 days and treated with TGF-β for 1, 3, 6, and 24 hr, and gene expression profiling was performed using Illumina HumanHT-12 BeadChips. The plot shows the number of TGF-β-dependent genes in each BTIC at each time point (false discovery rate [FDR] < 0.1; data presented on the $\log_2$ scale to show both the early and late response). TGF-β-dependent genes unique to MDA-MB-231 or HCC-1954 BTICs are labeled in red and blue, respectively, whereas genes regulated by TGF-β in both BTIC types are labeled in gray. The number of genes within each category is indicated within bars. Also see Table S1.

(D) Annotation of SMAD3 binding sites in the genomes of MDA-MB-231 BTICs (red) and HCC-1954 BTICs (blue). ChIP-seq was performed on 7-day-old mammospheres treated with TGF-β for 3 hr. See Supplemental Experimental Procedures for details.

(E) Heatmaps showing gene expression dynamics upon TGF-β stimulation in each BTIC independently. Bar plots below each gene expression time point show the proportion of TGF-β-dependent genes at that particular time point that were detected as bound by SMAD3 in the ChIP-seq experiment (bar below the 1-hr time point in HCC-1954 is absent as zero genes were detected as significantly differentially expressed). Asterisks indicate statistical significance, which was determined with the chi-square test, using TGF-β-independent gene sets as background (refer to Supplemental Experimental Procedures for details).

The transcriptional responses associated with the opposing effects of TGF-β on BTICs were characterized by gene expression profiling. BTIC-enriched mammosphere cultures (hereafter referred to as "BTICs") were treated with TGF-β for varying amounts of time (1, 3, 6, and 24 hr) to capture both early and late transcriptional responses. Comparing the lists of TGF-β-dependent genes revealed that only a small fraction is commonly regulated in both BTIC types ("shared" genes) (Figure 1C; Table S1). The vast majority of genes displayed cell-context-specific regulation, indicating that distinct and non-overlapping TGF-β-dependent transcriptional regulation occurs in BTICs with opposing (pro-oncogenic and tumor-suppressive) responses.

We previously showed that the BTIC-promoting and BTIC-suppressing effects of TGF-β depend on SMADs (Bruna et al., 2012). Hence, we hypothesized that SMADs mediate the TGF-β-dependent transcriptional regulation in both contexts. SMAD3 binding patterns in BTICs were mapped genome-wide after 3 hr of TGF-β exposure using chromatin immunoprecipitation and sequencing (ChIP-seq). We chose the 3-hr time point as it was the earliest at which significant TGF-β-dependent gene expression changes were detected in both models. Genomic annotation of SMAD3 binding sites showed a significant fraction of peaks (>30%) is directly associated with genes, with the remainder being at distal regulatory regions (Figure 1D).

We defined gene-proximal SMAD3 binding when peaks occurred within a genomic unit encompassing the 1,500 bp upstream of the transcription start site (TSS) to the end of the gene body. Comparing TGF-β-dependent and TGF-β-independent (background) gene sets revealed that both early- and late-responder genes are strongly enriched for gene-proximal SMAD3 binding in both BTIC types (Figure 1E). These data suggest that gene-proximal SMAD3 binding mediates TGF-β-dependent gene regulation in both contexts.

Differential SMAD3 Binding Is Not the Sole Determinant of Context-Specific Gene Regulation by TGF-β

The prevailing model for TGF-β context-dependent transcriptional regulation assumes binding of SMAD3 to different genes in different cell types (Massagué, 2012; Mullen et al., 2011).

Our data showed instead that a substantial proportion of SMAD3 binding sites are identical in both BTIC types (50% in MDA-MB-231 and 37% in HCC-1954) (Figure 2A). Motif analysis identified a number of distinct DNA motifs under SMAD3 binding sites (Figures S1A and S1C), including "canonical" SMAD consensus motifs (Figures S1D and S1E) (Dennler et al., 1998; Jonk et al., 1998; Koinuma et al., 2009; Shi et al., 1998; Zawel et al., 1998). The majority of identified motifs also corresponded to known SMAD binding partners (Figure S1B), which have been implicated in TGF-β responses by single gene studies (Gomis et al., 2006; Koinuma et al., 2009; Liberati et al., 1999; Massagué, 2012; Sundqvist et al., 2013; Xu et al., 2015; Zaidi et al., 2002). These results indicate that SMAD3 associates with diverse co-factors that guide it to both shared and cell-type-specific genomic locations in BTICs.

Inspection of ChIP-seq profiles around BTIC context-specific TGF-β-dependent genes revealed that SMAD3 binding is not necessarily associated with the regulation of the underlying gene, but rather can adopt four different binding modes (Figure 2B). For example, a gene regulated by TGF-β only in MDA-MB-231 BTICs (MDA-unique gene) can be: (1) uniquely bound by SMAD3 in MDA-MB-231 (binding mode 1), (2) uniquely bound by SMAD3 in HCC-1954 (binding mode 2), (3) commonly bound by SMAD3 in both cell types (binding mode 3), and (4) not bound by SMAD3 in either cell type (binding mode 4). The same applies to the TGF-β-dependent genes regulated uniquely in HCC-1954 BTICs (HCC-unique genes) (Figure 2B, bottom panels). These results differ from those previously reported using non-malignant cellular models, where TGF-β's cell-context-specific genes are almost exclusively associated with cell-type-specific SMAD3 binding patterns (Mullen et al., 2011).

We systematically investigated how these four SMAD3 binding modes contribute to gene regulation downstream of TGF-β. We found that TGF-β-dependent early-responder genes (derived 6 hr post-TGF-β treatment) are highly enriched for the common SMAD3 binding mode (mode 3) in both MDA-MB-231 and HCC-1954 BTICs (Figure 2C, upper table). TGF-β-dependent late-responder genes (derived 24 hr post-TGF-β treatment) show enrichment of both common (mode 3) and cell-type-unique SMAD3 binding (modes 1 and 2; Figure 2C, lower table). Notably, these BTIC type-unique SMAD3 binding events are associated with TGF-β-dependent genes in the corresponding model (MDA-unique genes with MDA-unique SMAD3 binding, and HCC-unique genes with HCC-unique SMAD3 binding) 24 hr after pathway activation. Genes not bound by SMAD3 in either cell type (mode 4) are relatively depleted in both the early and late TGF-β-responder genes, as could be expected based on the results presented in Figure 1E.

Based on the observed enrichment of the common SMAD3 binding mode in all gene groups and particularly in the early TGF-β responders, we conclude that cell-type-specific gene-proximal SMAD3 binding is not the sole determinant of context-specific TGF-β transcriptional responses.

In embryonic stem cells and muscle and lymphocyte progenitors, SMAD3 occupies distinct, non-overlapping sites within the gene, even when binding to the same gene (Mullen et al., 2011). To test if this also occurs in BTICs, we systematically categorized SMAD3 binding events into three classes: (1) uniquely present in MDA-MB-231 (Figure 2D, red peak), (2) uniquely present in HCC-1954 (Figure 2D, blue peak), and (3) present in identical position in both cell types (Figure 2D, gray peaks). For each context-specific TGF-β-dependent gene, we derived a composite SMAD3 binding profile (Figure 2D, right). This analysis revealed that only a small fraction of commonly bound genes (mode 3) possess mutually exclusive SMAD3 binding patterns (Figures 2E and 2F, light blue boxes). In fact, most genes that are commonly bound by SMAD3 (mode 3) display either a mixed occupancy profile, where both identical and cell-type-specific binding sites are present, or an identical occupancy profile, where SMAD3 binds at identical coordinates within a given gene in both cell types (Figures 2E and 2F). These findings led us to hypothesize that for many genes (at least 422 MDA-unique genes and 264 HCC-unique genes) possessing remarkably similar SMAD3 binding patterns in BTICs (yellow boxes, Figures 2E and 2F), other regulatory determinants might govern the context-specific transcriptional outputs of TGF-β.

We obtained similar results for SMAD3 binding events located distally to genes (Figures S2A–S2F; Supplemental Experimental Procedures); however, for simplicity, these are not presented in the Results section.

Context-Specific Epigenetic Landscape Modulates TGF-β/SMAD3-Dependent Transcriptional Regulation

In breast cancer, epigenetic modifications have characteristic, subtype-specific genomic patterns (Bediaga et al., 2010; Holm et al., 2010). We therefore reasoned that cell-type-specific epigenetic landscapes in BTICs could contribute to shaping the TGF-β transcriptional responses. We profiled the chromatin configuration in BTICs by mapping RNA polymerase II (Pol II) binding, histone H3 lysine 27 acetylation (H3K27ac), histone H3 lysine 4 trimethylation (H3K4me3), and histone H3 lysine 27 trimethylation (H3K27me3) using ChIP-seq. We also mapped CpG DNA methylation using methyl-binding domain pull-down and sequencing (MBD-seq). These epigenetic marks were profiled in untreated BTIC cultures to determine whether the "native" chromatin configuration existing prior to TGF-β stimulation was what modulated the context-specific transcriptional response.

Peak-based analysis showed the genomic distribution of the epigenetic marks occurred in the expected patterns: Pol II peaks localized predominantly to enhancer and promoter regions, H3K4me3 peaks to promoter regions, H3K27ac peaks to enhancer and promoter regions, H3K27me3 peaks to intergenic domains, and DNA methylation peaks to gene-proximal elements (Figure S3A). Comparative analysis revealed that MDA-MB-231 and HCC-1954 BTICs harbor distinct epigenetic landscapes (Figure S3B).

Overlaying the epigenetic marks with SMAD3 binding data showed that SMAD3 binds to open chromatin (marked by H3K27ac, Pol II, and H3K4me3) and not to closed chromatin (marked by H3K27me3 and DNA methylation) (Figure S3C). Additionally, BTIC type-specific SMAD3 binding coincided with the type-specific patterns of Pol II and H3K27ac (Figure S3D). This suggested that the pre-existing cell-type-specific chromatin context determines where SMAD3 binds upon TGF-β stimulation.

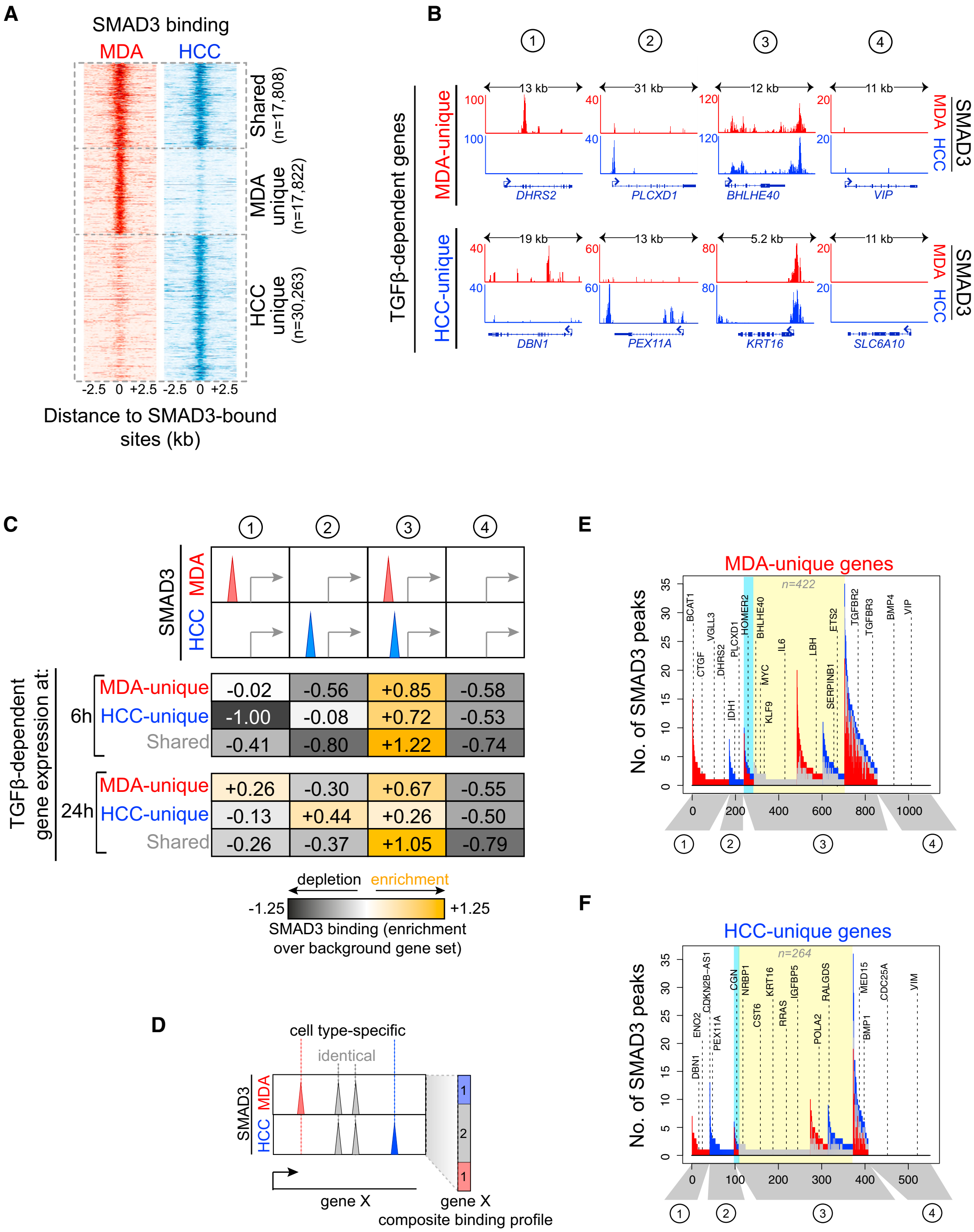

Figure 2. Differential SMAD3 Binding Is Not the Sole Determinant of Context-Specific Gene Regulation by TGF-β

(A) Occupancy plots showing SMAD3 binding sites in MDA-MB-231 (red) and HCC-1954 (blue) BTICs relative to each other, within the 5-kb window around the peak summits. Also see Figure S1.

(B) Gene tracks showing binding of SMAD3 in MDA-MB-231 (red) and HCC-1954 (blue) BTICs, at genes regulated by TGF-β only in MDA-MB-231 (top) and HCC-1954 (bottom) BTICs. SMAD3 adopts four modes of occupancy at these genes: bound in a cell-context-specific manner (modes 1 and 2), bound commonly in both BTIC types (mode 3), or not bound in either (mode 4).

(C) Genome-wide analysis showing the enrichment of each of the four SMAD3 binding modes (from B) at TGF-β-dependent genes. Gene expression data from 6-hr and 24-hr time points were used. Enrichment was calculated over SMAD3 binding distribution in the TGF-β-independent, background gene set (see

(legend continued on next page)

We next asked whether the pre-existing BTIC type-specific gene-proximal chromatin patterns prime genes for TGF-β-mediated regulation. To address this question, we combined differential binding analysis with gene set enrichment analysis (Figure 3A). This revealed that context-specific TGF-β-dependent genes are enriched for those with cell-type-specific epigenetic patterns, characterized by higher levels of gene-proximal open chromatin marks (H3K4me3, H3K27ac, and Pol II) (Figures 3B and 3C) and lower levels of repressive chromatin marks in the corresponding BTIC type (Figure S3E; HCC-unique genes depleted from H3K27me3; MDA-unique genes depleted from DNA methylation). We also noted that TGF-β-dependent genes unique to MDA-MB-231 showed higher levels of DNA methylation in HCC-1954 (Figure 3B). Together, these results show that distinct epigenetic landscapes in BTICs modulate context-specific responses to TGF-β: high levels of H3K4me3, H3K27ac, and Pol II in gene-proximal space permit, while TSS DNA methylation and H3K27me3 impede, TGF-β/SMAD3-dependent regulation of gene expression.

We next asked whether these epigenetic differences in the gene-proximal space act in synergy with, or independently of, differential SMAD3 binding to control context-specific TGF-β target gene regulation. For this purpose, genes with differential levels of SMAD3 were defined using the same analysis as for the chromatin factors (Figure 3A). This enabled us to stringently detect genes with the most pronounced differences in SMAD3 binding intensity between BTICs. For each TGF-β context-specific gene group (MDA unique and HCC unique), we derived three sets of signatures: SMAD3-high gene set (genes that display higher levels of SMAD3 in the corresponding BTIC type), open chromatin-high gene set (genes with higher levels of either H3K4me3, H3K27ac or Pol II in the corresponding BTIC type), and DNA hypo-methylation gene set (genes with lower levels of TSS DNA methylation in the corresponding BTIC type) (Table S2). Comparison of these gene sets in each BTIC type revealed that virtually all genes within the SMAD3-high set (58 in MDA and 30 in HCC) also belong to the open chromatin-high gene set (Figures 3D and 3E). This shows that in order to achieve type-specific gene regulation, differential binding of SMAD3 is assisted by gene-proximal open chromatin configuration, as shown for *IGDCC4* and *GRAMD2* (epigenome-assisted TGF-β-regulated genes; Figures 4A and 4B). Moreover, a substantial number of TGF-β-dependent genes in each BTIC type (401 MDA-unique genes; 181 HCC-unique genes) belonged to the open chromatin-high and/or DNA hypo-methylation sets, but not to the SMAD3-high set. Hence, the context-specific TGF-β-dependent regulation of the genes in this set is likely to be mediated by epigenetic differences (epigenome-directed TGF-β-regulated genes), as highlighted by *ADAM8* and *IGFBP5* (Figures 4A and 4B). This analysis also revealed that only a subset of SMAD3-high genes overlap with the DNA hypo-methylated set, suggesting that differential DNA methylation and differential SMAD3 binding appear to independently contribute to context-specific gene regulation by TGF-β.

Taken together, these results suggest that cell context-specific transcriptional responses to TGF-β are mediated by both SMAD3 and the epigenome. The epigenomic landscape primes genes for transcriptional regulation by TGF-β signaling, both in synergy with, and independently of, differential SMAD3 binding.

Differential DNA Methylation of *LBH* Impacts the BTIC-Promoting Effects of TGF-β

To functionally validate the impact of the epigenome on the opposing effects of TGF-β on BTICs, we focused on context-specific TGF-β-dependent genes with differential DNA methylation. Interesting links have been proposed between normal developmental processes and breast cancer (Holm et al., 2010; Prat et al., 2010), and therefore, we selected two genes encoding developmental TFs for further analysis: Limb Bud and Heart Development (*LBH*), and Vestigial-like family member 3 (*VGLL3*).

LBH and *VGLL3* are induced by TGF-β in a SMAD2/3 dependent manner in BTICs from MDA-MB-231, but not in HCC-1954 (Figures S4A–S4D). *LBH* is bound by SMAD3, and Pol II at an identical intragenic regulatory region in both cell types (Figure 5A), but in HCC-1954, the TSS-proximal region is DNA methylated (coinciding with lack of Pol II binding) (Figure 5A). This suggests that context-specific regulation of *LBH*, despite remarkably similar SMAD3 binding, is dependent on the methylation status of its promoter (epigenome directed). In contrast, *VGLL3* is bound by SMAD3 and Pol II only in MDA-MB-231 BTICs, while its TSS harbors DNA methylation only in HCC-1954 (Figure 5B). Hence, TGF-β-dependent regulation of *VGLL3* is epigenome assisted.

To test if promoter methylation of *LBH* and *VGLL3* determines their context-specific TGF-β-dependent transcriptional regulation, BTICs were treated with 5-aza-2′-deoxycytidine (5-aza-dC) prior to TGF-β stimulation, which resulted in reduction of overall methylation levels at these loci in HCC-1954 (Figures S4F and S4G). In HCC-1954, 5-aza-dC treatment reactivated both *LBH* and *VGLL3* expression, and TGF-β treatment further induced *LBH,* but not *VGLL3* (Figures 5C, 5D, and S4E). This shows that promoter DNA methylation is sufficient to block TGF-β/SMAD3-mediated induction of *LBH*. Erasure of DNA methylation from *VGLL3* failed to restore its TGF-β-dependent induction in HCC-1954, as predicted due to the absence of

Supplemental Experimental Procedures for details). Note that the common binding mode (3) does not exclude SMAD3 binding sites that occur on the same genes but on different sites in the two BTICs. Also see Figure S2.
(D) Schematic of the gene-based SMAD3 binding analysis. For each gene in the genome, the number of context-specific SMAD3 binding sites (red and blue) and shared binding sites (gray) were calculated and represented as a composite profile.
(E and F) Gene-based SMAD3 binding analysis on context-specific TGFβ-dependent genes (performed as outlined in D). TGF-β-dependent genes 24 hr post-TGF-β stimulation were used. Genes are aligned along the x axis and grouped into distinct categories based on their SMAD3 composite profiles. SMAD3 binding modes are indicated below the plot in gray. Gene examples are highlighted with dashed lines. The light blue box marks genes with mutually exclusive SMAD3 binding patterns, and the yellow box marks those with predominantly similar or identical SMAD3 binding patterns in both BTICs. Also see Figure S2. See Supplemental Experimental Procedures for details.

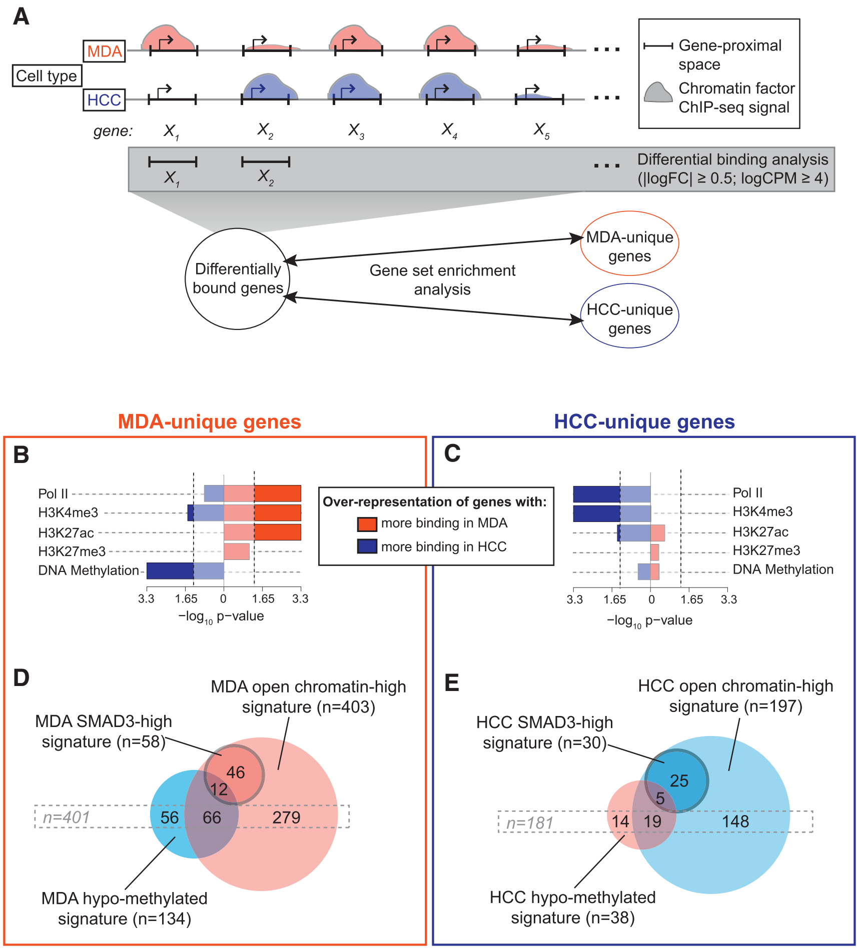

Figure 3. Epigenetic Wiring Confers Predisposition for Context-Specific TGF-β Responses

(A) Schematic of the analysis approach. Differences between BTICs in the levels of each factor were defined based on differential binding analysis within the gene-proximal space (1,500 bp upstream of the TSS to gene end), apart from DNA methylation, for which only TSS-proximal regions were considered (−1,500 bp to +1,500 bp around the TSS). Gene set enrichment analysis was then conducted, testing the enrichment of differentially bound gene sets within the context-specific TGF-β-dependent gene sets (MDA-unique and HCC-unique genes). Refer to Supplemental Experimental Procedures for details.

(B and C) Over-representation of genes with cell-type-specific levels of epigenetic modifications and Pol II, within the context-specific TGF-β-dependent genes (MDA-unique on the left and HCC-unique on the right). The significance of enrichment is represented as a p value on a bi-symmetrical x axis. The left and the right sides of the axis correspond to the enrichment of genes with more binding of the corresponding mark in HCC-1954 (blue) and MDA-MB-231 (red), respectively. p value cutoffs were set at 0.05 ($-\log_{10}(1.33) = 0.05$) (dashed lines). TGF-β-dependent genes derived at the 24-hr time point were used (see Supplemental Experimental Procedures for details). Also see Figure S3.

(D and E) Comparison of SMAD3-high, open chromatin-high, and DNA hypo-methylation gene sets within the MDA-unique and HCC-unique TGF-β-dependent genes (24-hr gene expression time point). For each BTIC, SMAD3-high, open chromatin-high, and DNA hypo-methylation gene sets were defined as groups of genes with differentially higher SMAD3 levels, differentially higher open chromatin levels, and differentially lower DNA methylation levels when compared to the opposing BTIC type (differential binding analysis performed as in A). Also see Table S2.

SMAD3 binding at this locus. Taken together, these results confirm that the epigenetic configuration not only determines baseline gene expression levels, but it also controls TGF-β/SMAD3-dependent transcriptional regulation.

To assess the functional implications of epigenome-directed and epigenome-assisted mechanisms, we investigated whether *LBH* and *VGLL3* are required for the effects of TGF-β on BTICs. We knocked down their expression using short interfering RNAs (siRNAs), resulting in 80% and 50% reduction of *LBH* and *VGLL3* transcript levels, respectively (Figures S5A and S5B). Mammosphere-initiating cell (MS-IC) and colony-forming cell (CFC) assays were used to test self-renewal and proliferation of BTICs (Bruna et al., 2012; Dontu et al., 2003a, 2003b).

LBH knockdown in untreated cells reduced BTIC self-renewal and proliferation in both cell lines (Figures 6A and 6B), suggesting that *LBH* is required for baseline BTIC maintenance regardless of the response to TGF-β. In HCC-1954 BTICs, *LBH* transcripts are expressed at very low levels despite promoter methylation, and their reduction by siRNA treatment (Figure S5A) results in measurable effects in the BTIC assays (Figures 6A and 6B). These *LBH* transcripts are likely to originate from low levels of transcription initiated at methylated DNA molecules with variegated CpG methylation patterns (“epipolymorphisms”; Landan et al., 2012), as determined by reduced representation bisulfite sequencing (RRBS) (Figure 6C). Thus, residual transcription initiated at epipolymorphic promoters can be functionally important.

In MDA-MB-231, *LBH* depletion impaired the BTIC-promoting effects of TGF-β by more than 2-fold (Figures 6A and 6B). In contrast, in HCC-1954, *LBH* depletion did not affect BTIC suppression by TGF-β (Figures 6A and 6B). In both cells lines, *VGLL3* depletion had no effect on BTICs after TGF-β treatment (Figures 6A and 6B). Altogether, these results suggest that epigenome-directed gene co-regulation with SMAD3, as occurs with *LBH*, acts as a molecular switch that mediates the opposing effects of TGF-β on BTICs.

We previously showed that TGF-β specifically promotes BTIC activity only in Claudin^{low} cell lines (Bruna et al., 2012). We also showed that in normal mammary epithelium, TGF-β promotes mammary stem cells (the presumed cell of origin of Claudin^{low} cancers), and it inhibits luminal progenitors (Bruna et al., 2012). Interestingly, others have shown that in normal breast epithelial

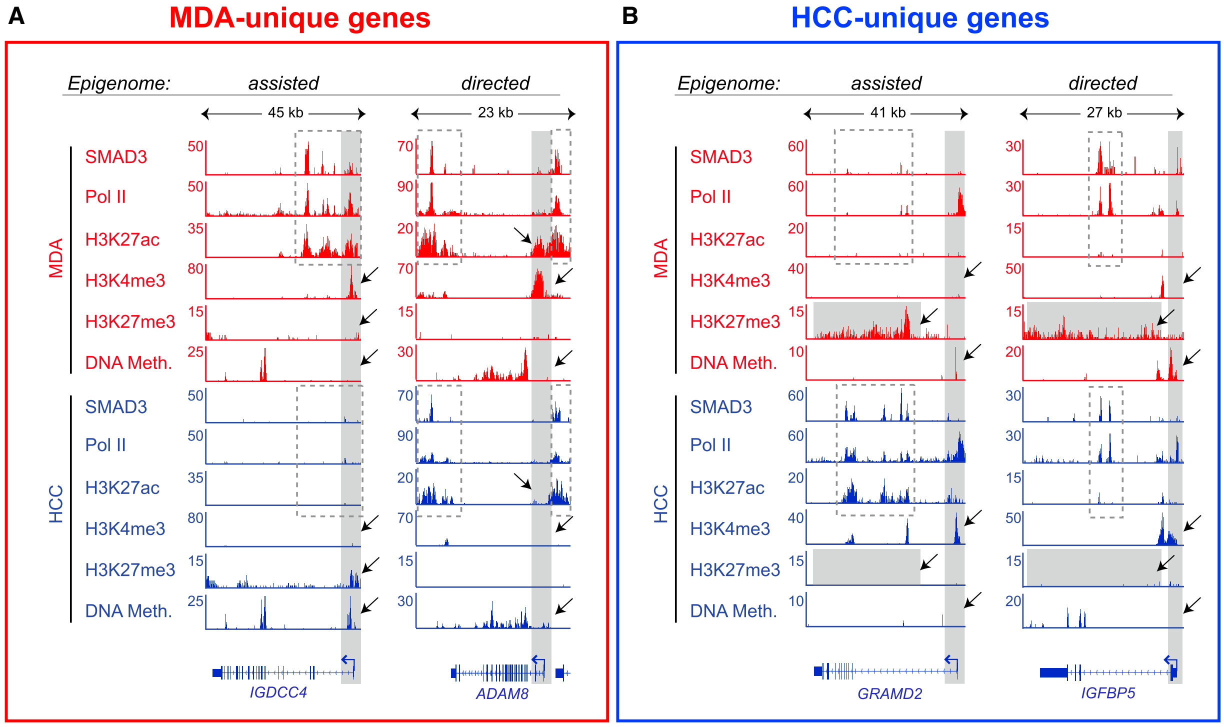

Figure 4. Epigenome either Assists or Directs Context-Specific Target Gene Regulation by TGF-β/SMAD3

(A and B) Examples illustrating the epigenome-assisted and epigenome-directed modes for target-gene regulation by TGF-β/SMAD3. ChIP-seq tracks for MDA-MB-231 are shown in red shades and for HCC-1954 in blue shades. Two MDA-unique (*IGDCC4* and *ADAM8*) and two HCC-unique (*GRAMD2* and *IGFBP5*) TGF-β-dependent genes are shown. In the epigenome-assisted mode, differential SMAD3 binding patterns (dashed boxes) are coupled with differential epigenetic configuration (gray boxes and arrows). In the epigenome-directed mode, SMAD3 binding patterns are the same in both BTICs (dashed boxes), while epigenetic differences (gray boxes and arrows) are associated with cell-type-specific gene regulation by TGF-β.

tissue, *LBH* promotes stemness and inhibits differentiation (Lindley et al., 2015; Rieger et al., 2010). We therefore sought evidence for a relevant role of *LBH* in both normal breast epithelium and in breast cancer. Analysis of gene expression data from normal human and mouse mammary epithelium revealed that *LBH* is highly expressed in the basal (stem cell-containing) compartment and is downregulated as cells differentiate along the luminal lineage (Figures 6F, S5C, and S5D). Investigation of gene expression data from 1,980 primary breast cancers (Curtis et al., 2012) showed that *LBH* expression is highest in the Claudin^{low} subtype (Figure 6D). In patients with Claudin^{low} tumors, higher *LBH* expression correlates with worse survival (Figure 6E). These findings suggest that the BTIC context-specific TGF-β/*LBH* observations we made in model cell lines are relevant to both normal and malignant primary tissue biology.

DISCUSSION

The mechanisms underlying the opposing TGF-β effects in cancer cells, being both pro-oncogenic and tumor suppressive, remain a significant challenge for inhibition of the pathway as a feasible cancer therapeutic strategy in the clinic. The current understanding is that TGF-β stimulation results in different responses in distinct cell types through the association of SMAD2/3 with specific SMAD cofactors (Massagué, 2008, 2012). Accordingly, in normal cells, along a developmental cascade, SMAD3 co-occupies distinct genomic locations in association with cell-type-specific master transcription factors: Oct4 in embryonic stem cells, Myod1 in myotubes, and PU.1 in pro-B cells (Mullen et al., 2011). These cofactors are required for SMAD3 binding, and most TGF-β-regulated genes are bound by these master TFs (Mullen et al., 2011). In other words, the currently accepted model suggests that master TFs are responsible for instructing the gene targets downstream of TGF-β signaling and thus determine its cell-type-specific effects (Mullen et al., 2011). In cancer cells, no similar genome-wide studies have been conducted, but single-gene studies appear to show analogous findings: TF switches (where SMADs exchange binding partners) can occur and result in redirecting of SMADs from the promoters of tumor suppressor genes to promoters of oncogenes, concomitant with altered transcription of those target genes (Gomis et al., 2006; Seoane et al., 2004; Xu et al., 2015).

Our results show for the first time that different SMAD3 binding patterns cannot fully account for the observed differences in the TGF-β-dependent transcriptional responses associated with promotion or suppression of BTICs. In fact, and surprisingly, the majority of BTIC context-specific TGF-β-dependent genes, particularly early-responder genes, are bound by SMAD3 in both contexts. While binding may occur in distinct locations

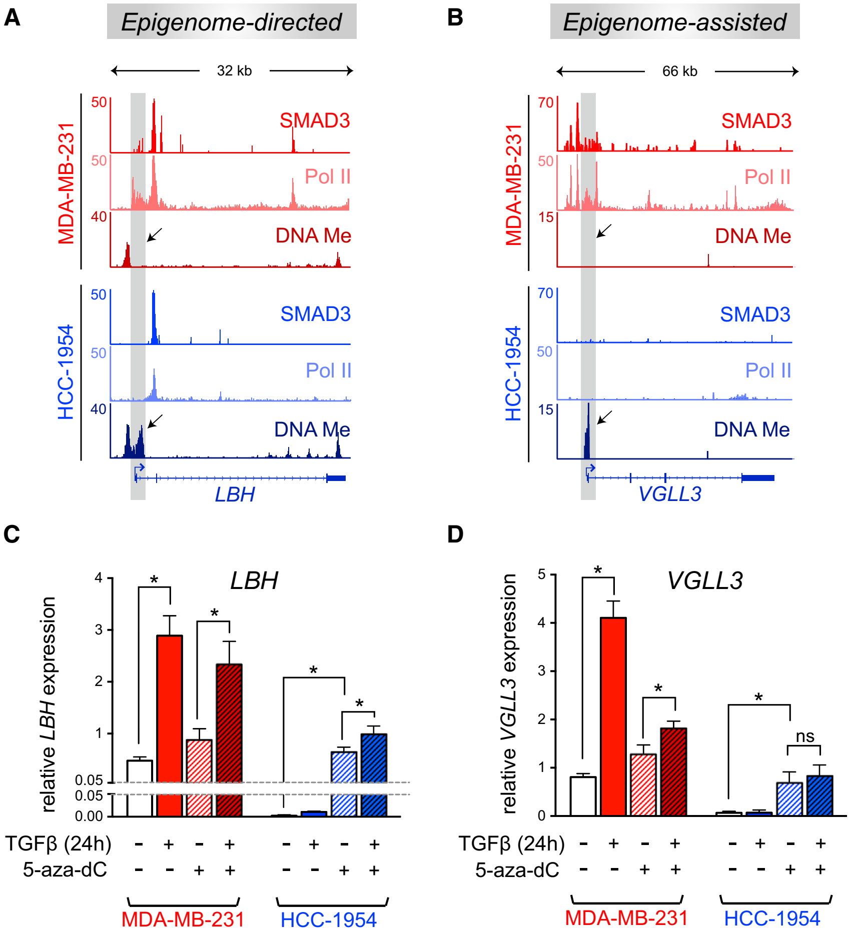

Figure 5. DNA Methylation Regulates TGF-β-Dependent Induction of *LBH*

(A and B) Gene tracks showing SMAD3/Pol II binding and DNA methylation at *LBH* and *VGLL3* loci. ChIP-seq tracks for MDA-MB-231 are shown in red shades and for HCC-1954 in blue shades. Also see Figure S4.

(C and D) qRT-PCRs showing the expression of *LBH* and *VGLL3* transcripts upon 5-aza-dC and TGF-β treatments. Cells were treated with 5-aza-dC for 5 days, seeded, and allowed to form mammospheres for 7 days, and then stimulated with TGF-β for 24 hr. Data were normalized to the housekeeping (*RBM22*) transcript levels and are presented as mean ± SD of three biological replicates. Asterisks indicate significant differences. ns, not significant (one-way ANOVA). Also see Figure S4.

along the gene, a large fraction of genes possessed coherent SMAD3 occupancy profiles, many with only identical SMAD3 binding sites. These results reveal that TGF-β-dependent cell-type-specific transcriptional regulation in cancer cells is not universally mediated by differential SMAD3 binding. This prompted us to analyze whether additional regulatory mechanisms operating on chromatin modulate the context-specific target gene selection by TGF-β/SMAD3.

Very recently, it has been reported that epigenetic configuration of somatic cells predisposes them to reprogramming fates (Pour et al., 2015). Here we show that tumor initiating cells harbor distinct epigenetic landscapes that prime specific gene sets for regulation by TGFβ. These distinct epigenetic configurations can act both in synergy with cell-type-specific SMAD3 binding (epigenome-assisted), and independently of cell-type-specific SMAD3 binding (epigenome-directed), to control TGFβ/SMAD3-dependent context-specific regulation of target genes (Figure 7).

We propose that epigenome-directed priming in cancer cells might be a prevalent way of instructing context-specific TGFβ effects. Cancer cells that originate in the same tissue (mammary epithelium in the case of BTICs), unlike cells from distinct tissue lineages, are likely to possess similar master TF wiring. But cancers with the same tissue of origin can possess markedly different epigenomes, for example, DNA methylation of gene promoters (Holm et al., 2010). Here, we reveal an unexpected similarity of SMAD3 binding patterns in BTICs with opposing transcriptional responses to TGF-β and show that context-specific TGF-β-dependent genes are frequently regulated by an epigenome-directed, DNA-methylation-dependent mechanism, rather than by differential SMAD3 binding. These results at the whole-genome level expand a previous observation in glioma, where the methylation status of *PDGFB* predisposes tumor cells for either an oncogenic or a tumor-suppressive response to TGF-β signaling (Bruna et al., 2007).

We have identified *LBH*, a regulator of epithelial differentiation in the mammary gland (Lindley et al., 2015; Rieger et al., 2010), as a mediator required for the context-specific BTIC-promoting effects of TGF-β, depending on its cell-type-specific methylation state. The patterns of expression of *LBH* in normal mammary development and in human breast cancers are consistent with its role as a context-specific TGF-β target in primary tissues. We speculate that many epigenome-directed genes behave like *LBH* to mediate the context-specific effects of TGF-β in cancer.

The model we propose here (Figure 7), that regulation of transcriptional programs by extracellular growth factors is dependent on the context-specific epigenomic landscapes of cancer cells, might not be specific to TGF-β and could have broader implications for the paracrine effects of the microenvironment on the malignant compartment of cancers.

EXPERIMENTAL PROCEDURES

Cell Manipulation and Mammosphere Cultures

MDA-MB-231 and HCC-1954 breast cancer cell lines were enriched for BTICs by mammosphere cultures, as described previously (Bruna et al., 2012; Dontu et al., 2003a, 2003b). To activate TGF-β signaling, mammospheres were treated with 0.1 nM recombinant TGF-β1. *LBH*, *VGLL3*, *SMAD2* and *SMAD3* levels were manipulated using siRNA pools (GE

OPEN
ACCESS
CellPress

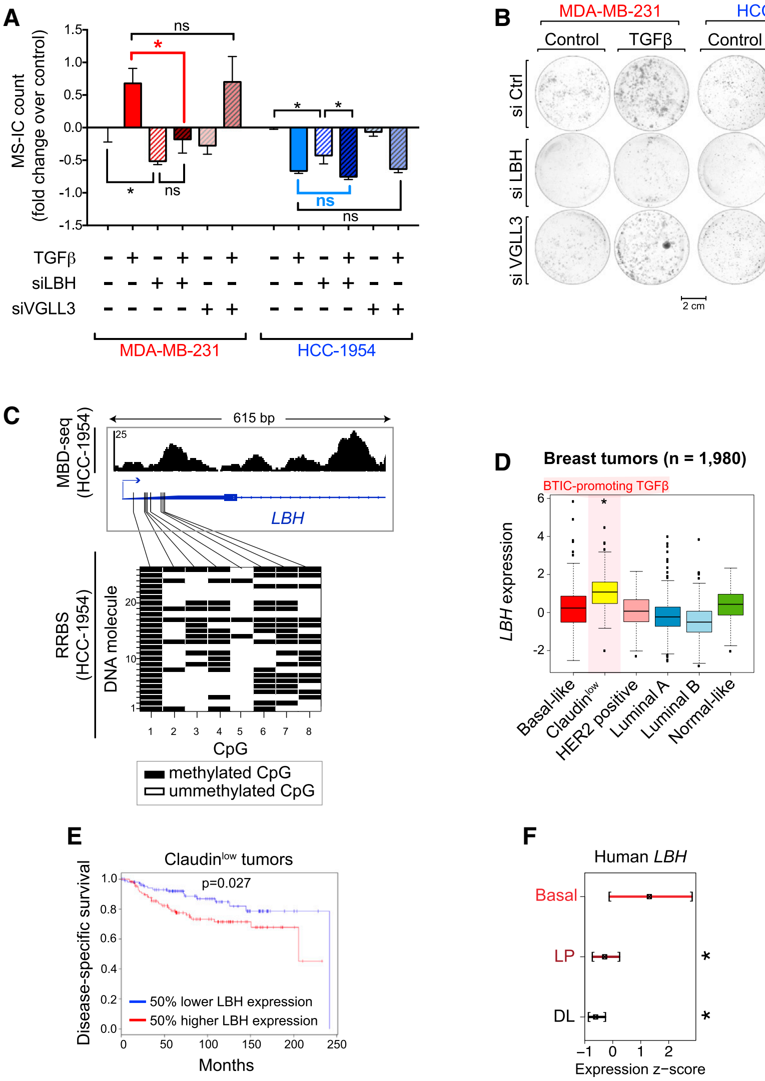

Figure 6. *LBH* Is Necessary for TGF-β's BTIC-Promoting Activity

(A) MS-IC assay showing the changes in MS-IC numbers upon TGF-β treatment and siRNA-mediated knockdown of *LBH* and *VGLL3*. Mammospheres were simultaneously treated with TGF-β and siRNA against *LBH* and *VGLL3* for 7 days, second-generation mammospheres were seeded, and an MS-IC assay was performed (see Supplemental Experimental Procedures). Data are presented as mean ± 95% confidence interval (CI) of nine replicates. Asterisks indicate significant differences. ns, not significant (one-way ANOVA). Also see Figure S5.

(B) CFC assay showing the effects of TGF-β and siRNA-mediated knockdown of *LBH* and *VGLL3* on the proliferation of BTICs.

(C) RRBS analysis of the *LBH* promoter. x axis shows eight adjacent CpG sites within the *LBH* promoter (highlighted on top); y axis shows binary methylation calls for each CpG site within 26 sequenced DNA molecules. Genomic track on top represents the DNA methylation profile of HCC-1954 BTICs derived by MBD-seq.

(D) Box plots showing the expression of *LBH* in different breast cancer subtypes. Significance was determined by a linear model (ANOVA) and simultaneous tests comparing each group to the mean (see Supplemental Experimental Procedures). Gene expression data are obtained from the METABRIC cohort (Curtis et al., 2012).

(E) Survival analysis showing the relationship between *LBH* expression and disease-free survival in the Claudin^{low} patient group. Patients were stratified based on top and bottom halves of *LBH* expression. Survival function was estimated using the Kaplan-Meier estimator, and differences between groups were tested with the log-rank test (see Supplemental Experimental Procedures).

(F) Expression of *LBH* in different cell compartments of normal human mammary epithelium. Basal compartment, luminal progenitors (LP), and differentiated luminal cells (DL) are shown. Significance was determined by a linear model (ANOVA) comparing LP and DL expression to the basal group. Data from Shehata et al. (2012). Also see Figure S5.

Healthcare). To achieve global DNA demethylation, the cells were treated with 1 μM 5-aza-2′-deoxycytidine. For full details, see Supplemental Experimental Procedures.

Chromatin Immunoprecipitation and Sequencing

ChIP-seq was performed using a custom-developed protocol. Briefly, mammospheres (treated with 0.1 nM TGF-β for 3 hr for SMAD3 ChIP-seq, and untreated in all other experiments) were crosslinked for 45 min with Di(N-succinimidyl) glutarate (DSG) and 30 min with formaldehyde. Chromatin was extracted and then sheared using Covaris. Immunoprecipitation was performed with 10 μg of the corresponding antibodies and protein G agarose beads (Santa Cruz Biotechnology). Libraries were prepared with TruSeq LT kit (Illumina) and sequenced on HiSeq 2000 (Illumina). For the full protocol, see Supplemental Experimental Procedures.

DNA Methylation Profiling

For MBD-seq, methylated DNA was precipitated with recombinant methyl binding domain (MBD2b/MBD3L1) protein complex as part of MethylCollector Ultra kit (Active Motif), following the manufacturer's recommendations. Libraries were generated using TruSeq LT kit (Illumina) and sequenced on HiSeq 2000 (Illumina). Refer to Supplemental Experimental Procedures for details.

RRBS was performed as described previously (Boyle et al., 2012).

Targeted bisulfite sequencing was performed using a custom-developed method (refer to Supplemental Experimental Procedures for details).

ChIP-Seq and MBD-Seq Data Analysis

Sequencing reads were filtered based on quality and aligned to the Human Genome Build 37 (hg19) using BWA (Li and Durbin, 2009). For ChIP-seq, SMAD3 peaks were called using MACS (Zhang et al., 2008), and SICER (Zang et al., 2009) was used for all other factors profiled. For MBD-seq, bi-asymmetric-Laplace model (BALM) was used to call methylation peaks (Lan et al., 2011), and (MeD)IP-seq data analysis (MEDIPS) was used for quantitative analysis, whereby the data were normalized to the CG content (Lienhard et al., 2014). Downstream analysis of all datasets was performed in R statistical software (R Development Core Team, 2009), using edgeR (v3.8.5) for differential binding analysis (Robinson et al., 2010) and annovar (2014nov12) for annotation (Wang et al., 2010). Motif analysis was performed in MEME-ChIP (Machanick and Bailey, 2011). Tracks representing genomic data were derived from IGV

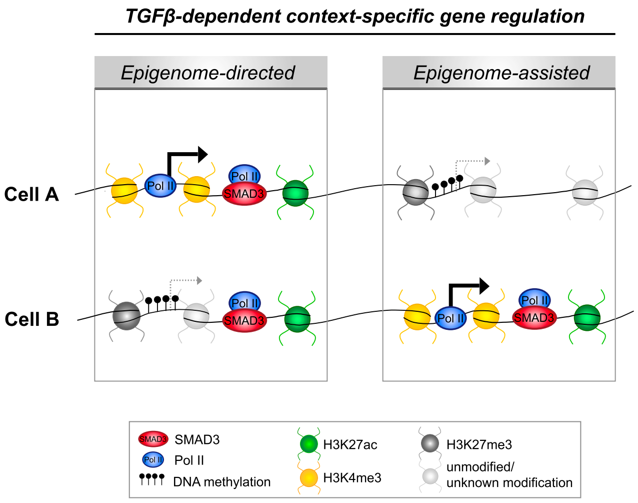

Figure 7. Context-Specific Effects of TGF-β/SMAD3 Are Modulated by the Epigenome

A model depicting how cell-type-specific epigenetic configurations determine context-specific effects of TGF-β/SMAD3. Genes differentially bound by SMAD3 also possess differential levels of open and closed chromatin modifications that will participate in specifying TGF-β-dependent expression of those genes (epigenome-assisted mechanism). Genes commonly bound by SMAD3 rely on the underlying cell-type-specific epigenetic configuration for determining their context-specific regulation by TGF-β (epigenome-directed mechanism).

(Robinson et al., 2011). Refer to Supplemental Experimental Procedures for details.

Gene Expression Analysis

Gene expression upon TGF-β induction was profiled using Illumina HumanHT-12 BeadChips. Data were analyzed as previously described (Smyth, 2005). Refer to Supplemental Experimental Procedures for details.

qRT-PCR

Reverse transcription was performed using Transcriptor First Strand cDNA Synthesis Kit (Roche), as recommended by the manufacturer. qPCR was performed using TaqMan Fast Universal PCR Master Mix and gene-specific TaqMan probes (Applied Biosystems). Refer to Supplemental Experimental Procedures for details.

Western Blots

The cells were grown as mammospheres for 7 days then treated with TGF-β for 1 hr. Total cell lysates were collected, and 20 μg protein was run per condition on the 10% SDS-PAGE gels. Transfer to nitrocellulose membranes was conducted using semi-dry blotting system (Invitrogen). Membranes were blocked in 5% dried milk powder, 0.1% Tween-20 in PBS, and the following antibodies were used for protein detection: rabbit monoclonal against human phospho-SMAD2 (Ser 465/467) (Cell Signaling, 3108), rabbit polyclonal against human SMAD2/3 (Santa Cruz Biotechnology, sc-8332) and rabbit polyclonal against human β-actin (Abcam, ab8227).

Mammosphere-Initiating Cell and Colony-Forming Cell Assays

MS-IC and CFC assays were performed as previously described (Dontu et al., 2003a, 2003b). Refer to Supplemental Experimental Procedures for details.

ACCESSION NUMBERS

The accession number for the ChIP-seq, MBD-seq, and gene expression data reported in this paper is EGA: EGAS00001001570.

SUPPLEMENTAL INFORMATION

Supplemental Information includes Supplemental Experimental Procedures, five figures, and two tables and can be found with this article online at http://dx.doi.org/10.1016/j.celrep.2015.11.040.

AUTHOR CONTRIBUTIONS

C.C. and A.B. supervised the study. A.T.V., A.B., and C.C. conceived and designed experiments. A.T.V., S.J.V., A.S.B., M.A.G., S.U.-L., and W.G. performed experiments. O.M.R. and A.T.V. performed data analysis. A.T.V., A.B., C.C., and P.J.C. interpreted experiments. A.T.V., C.C., and A.B. wrote the manuscript, incorporating edits from all authors.

ACKNOWLEDGMENTS

We thank Duncan T. Odom for helpful discussions. We are grateful to CRUK Cambridge Institute Bioinformatics and Genomics Cores, particularly Suraj Menon and Rory Stark, for processing of the raw sequencing data, read alignment, and peak calling. We thank Lukas Chavez for help with the MEDIPS package, Wei Shin for help with the Rsubread package, and Jose Luis Sandoval for help with microscopy. We are particularly grateful to CRUK for supporting A.T.V., O.M.R., A.S.B., M.A.G., W.G., A.B., and C.C. (RG69667). S.J.V. was supported by a grant from the Dutch Cancer Foundation (KWF).

Received: May 28, 2015
Revised: September 12, 2015
Accepted: November 11, 2015
Published: December 10, 2015

REFERENCES

Bediaga, N.G., Acha-Sagredo, A., Guerra, I., Viguri, A., Albaina, C., Ruiz Diaz, I., Rezola, R., Alberdi, M.J., Dopazo, J., Montaner, D., et al. (2010). DNA methylation epigenotypes in breast cancer molecular subtypes. Breast Cancer Res. *12*, R77.

Bierie, B., and Moses, H.L. (2009). Gain or loss of TGFbeta signaling in mammary carcinoma cells can promote metastasis. Cell Cycle *8*, 3319–3327.

Boyle, P., Clement, K., Gu, H., Smith, Z.D., Ziller, M., Fostel, J.L., Holmes, L., Meldrim, J., Kelley, F., Gnirke, A., and Meissner, A. (2012). Gel-free multiplexed reduced representation bisulfite sequencing for large-scale DNA methylation profiling. Genome Biol. *13*, R92.

Bruna, A., Darken, R.S., Rojo, F., Ocaña, A., Peñuelas, S., Arias, A., Paris, R., Tortosa, A., Mora, J., Baselga, J., and Seoane, J. (2007). High TGFbeta-Smad activity confers poor prognosis in glioma patients and promotes cell proliferation depending on the methylation of the PDGF-B gene. Cancer Cell *11*, 147–160.

Bruna, A., Greenwood, W., Le Quesne, J., Teschendorff, A., Miranda-Saavedra, D., Rueda, O.M., Sandoval, J.L., Vidakovic, A.T., Saadi, A., Pharoah, P., et al. (2012). TGFβ induces the formation of tumour-initiating cells in claudinlow breast cancer. Nat. Commun. *3*, 1055.

Cancer Genome Atlas Network (2012). Comprehensive molecular portraits of human breast tumours. Nature *490*, 61–70.

Curtis, C., Shah, S.P., Chin, S.F., Turashvili, G., Rueda, O.M., Dunning, M.J., Speed, D., Lynch, A.G., Samarajiwa, S., Yuan, Y., et al.; METABRIC Group (2012). The genomic and transcriptomic architecture of 2,000 breast tumours reveals novel subgroups. Nature *486*, 346–352.

Dennler, S., Itoh, S., Vivien, D., ten Dijke, P., Huet, S., and Gauthier, J.M. (1998). Direct binding of Smad3 and Smad4 to critical TGF beta-inducible elements in the promoter of human plasminogen activator inhibitor-type 1 gene. EMBO J. *17*, 3091–3100.

Dontu, G., Abdallah, W.M., Foley, J.M., Jackson, K.W., Clarke, M.F., Kawamura, M.J., and Wicha, M.S. (2003a). In vitro propagation and transcriptional profiling of human mammary stem/progenitor cells. Genes Dev. *17*, 1253–1270.

Dontu, G., Al-Hajj, M., Abdallah, W.M., Clarke, M.F., and Wicha, M.S. (2003b). Stem cells in normal breast development and breast cancer. Cell Prolif. *36* (*Suppl 1*), 59–72.

Gomis, R.R., Alarcón, C., Nadal, C., Van Poznak, C., and Massagué, J. (2006). C/EBPbeta at the core of the TGFbeta cytostatic response and its evasion in metastatic breast cancer cells. Cancer Cell *10*, 203–214.

Guasch, G., Schober, M., Pasolli, H.A., Conn, E.B., Polak, L., and Fuchs, E. (2007). Loss of TGFbeta signaling destabilizes homeostasis and promotes squamous cell carcinomas in stratified epithelia. Cancer Cell *12*, 313–327.

Holm, K., Hegardt, C., Staaf, J., Vallon-Christersson, J., Jönsson, G., Olsson, H., Borg, A., and Ringnér, M. (2010). Molecular subtypes of breast cancer are associated with characteristic DNA methylation patterns. Breast Cancer Res. *12*, R36.

Jonk, L.J., Itoh, S., Heldin, C.H., ten Dijke, P., and Kruijer, W. (1998). Identification and functional characterization of a Smad binding element (SBE) in the JunB promoter that acts as a transforming growth factor-beta, activin, and bone morphogenetic protein-inducible enhancer. J. Biol. Chem. *273*, 21145–21152.

Koinuma, D., Tsutsumi, S., Kamimura, N., Taniguchi, H., Miyazawa, K., Sunamura, M., Imamura, T., Miyazono, K., and Aburatani, H. (2009). Chromatin immunoprecipitation on microarray analysis of Smad2/3 binding sites reveals roles of ETS1 and TFAP2A in transforming growth factor beta signaling. Mol. Cell. Biol. *29*, 172–186.

Lan, X., Adams, C., Landers, M., Dudas, M., Krissinger, D., Marnellos, G., Bonneville, R., Xu, M., Wang, J., Huang, T.H., et al. (2011). High resolution detection and analysis of CpG dinucleotides methylation using MBD-Seq technology. PLoS ONE *6*, e22226.

Landan, G., Cohen, N.M., Mukamel, Z., Bar, A., Molchadsky, A., Brosh, R., Horn-Saban, S., Zalcenstein, D.A., Goldfinger, N., Zundelevich, A., et al. (2012). Epigenetic polymorphism and the stochastic formation of differentially methylated regions in normal and cancerous tissues. Nat. Genet. *44*, 1207–1214.

Li, H., and Durbin, R. (2009). Fast and accurate short read alignment with Burrows-Wheeler transform. Bioinformatics *25*, 1754–1760.

Liberati, N.T., Datto, M.B., Frederick, J.P., Shen, X., Wong, C., Rougier-Chapman, E.M., and Wang, X.F. (1999). Smads bind directly to the Jun family of AP-1 transcription factors. Proc. Natl. Acad. Sci. USA *96*, 4844–4849.

Lienhard, M., Grimm, C., Morkel, M., Herwig, R., and Chavez, L. (2014). MEDIPS: genome-wide differential coverage analysis of sequencing data derived from DNA enrichment experiments. Bioinformatics *30*, 284–286.

Lindley, L.E., Curtis, K.M., Sanchez-Mejias, A., Rieger, M.E., Robbins, D.J., and Briegel, K.J. (2015). The WNT-controlled transcriptional regulator LBH is required for mammary stem cell expansion and maintenance of the basal lineage. Development *142*, 893–904.

Machanick, P., and Bailey, T.L. (2011). MEME-ChIP: motif analysis of large DNA datasets. Bioinformatics *27*, 1696–1697.

Mani, S.A., Guo, W., Liao, M.J., Eaton, E.N., Ayyanan, A., Zhou, A.Y., Brooks, M., Reinhard, F., Zhang, C.C., Shipitsin, M., et al. (2008). The epithelial-mesenchymal transition generates cells with properties of stem cells. Cell *133*, 704–715.

Massagué, J. (2008). TGFbeta in Cancer. Cell *134*, 215–230.

Massagué, J. (2012). TGFβ signalling in context. Nat. Rev. Mol. Cell Biol. *13*, 616–630.

Massagué, J., Seoane, J., and Wotton, D. (2005). Smad transcription factors. Genes Dev. *19*, 2783–2810.

Mullen, A.C., Orlando, D.A., Newman, J.J., Lovén, J., Kumar, R.M., Bilodeau, S., Reddy, J., Guenther, M.G., DeKoter, R.P., and Young, R.A. (2011). Master transcription factors determine cell-type-specific responses to TGF-β signaling. Cell *147*, 565–576.

Pour, M., Pilzer, I., Rosner, R., Smith, Z.D., Meissner, A., and Nachman, I. (2015). Epigenetic predisposition to reprogramming fates in somatic cells. EMBO Rep. *16*, 370–378.

Prat, A., Parker, J.S., Karginova, O., Fan, C., Livasy, C., Herschkowitz, J.I., He, X., and Perou, C.M. (2010). Phenotypic and molecular characterization of the claudin-low intrinsic subtype of breast cancer. Breast Cancer Res. *12*, R68.

R Development Core Team (2009). R: A Language and Environment for Statistical Computing (R Foundation for Statistical Computing).

Rieger, M.E., Sims, A.H., Coats, E.R., Clarke, R.B., and Briegel, K.J. (2010). The embryonic transcription cofactor LBH is a direct target of the Wnt signaling pathway in epithelial development and in aggressive basal subtype breast cancers. Mol. Cell. Biol. *30*, 4267–4279.

Robinson, M.D., McCarthy, D.J., and Smyth, G.K. (2010). edgeR: a Bioconductor package for differential expression analysis of digital gene expression data. Bioinformatics *26*, 139–140.

Robinson, J.T., Thorvaldsdóttir, H., Winckler, W., Guttman, M., Lander, E.S., Getz, G., and Mesirov, J.P. (2011). Integrative genomics viewer. Nat. Biotechnol. *29*, 24–26.

Scheel, C., Eaton, E.N., Li, S.H., Chaffer, C.L., Reinhardt, F., Kah, K.J., Bell, G., Guo, W., Rubin, J., Richardson, A.L., and Weinberg, R.A. (2011). Paracrine and autocrine signals induce and maintain mesenchymal and stem cell states in the breast. Cell *145*, 926–940.

Seoane, J., Le, H.V., Shen, L., Anderson, S.A., and Massagué, J. (2004). Integration of Smad and forkhead pathways in the control of neuroepithelial and glioblastoma cell proliferation. Cell *117*, 211–223.

Shehata, M., Teschendorff, A., Sharp, G., Novcic, N., Russell, I.A., Avril, S., Prater, M., Eirew, P., Caldas, C., Watson, C.J., and Stingl, J. (2012). Phenotypic and functional characterisation of the luminal cell hierarchy of the mammary gland. Breast Cancer Res. *14*, R134.

Shi, Y., Wang, Y.F., Jayaraman, L., Yang, H., Massagué, J., and Pavletich, N.P. (1998). Crystal structure of a Smad MH1 domain bound to DNA: insights on DNA binding in TGF-beta signaling. Cell *94*, 585–594.

Smyth, G.K. (2005). Limma: linear models for microarray data. In Bioinformatics and Computational Biology Solutions Using R and Bioconductor, R. Gentleman, V. Carey, S. Dudoit, R. Irizarry, and W. Huber, eds. (Springer Science+Business Media), pp. 397–420.

Sundqvist, A., Zieba, A., Vasilaki, E., Herrera Hidalgo, C., Söderberg, O., Koinuma, D., Miyazono, K., Heldin, C.H., Landegren, U., Ten Dijke, P., and van Dam, H. (2013). Specific interactions between Smad proteins and AP-1 components determine TGFβ-induced breast cancer cell invasion. Oncogene *32*, 3606–3615.

Wang, K., Li, M., and Hakonarson, H. (2010). ANNOVAR: functional annotation of genetic variants from high-throughput sequencing data. Nucleic Acids Res. *38*, e164.

Xu, J., Acharya, S., Sahin, O., Zhang, Q., Saito, Y., Yao, J., Wang, H., Li, P., Zhang, L., Lowery, F.J., et al. (2015). 14-3-3ζ turns TGF-β's function from tumor suppressor to metastasis promoter in breast cancer by contextual changes of Smad partners from p53 to Gli2. Cancer Cell *27*, 177–192.

Zaidi, S.K., Sullivan, A.J., van Wijnen, A.J., Stein, J.L., Stein, G.S., and Lian, J.B. (2002). Integration of Runx and Smad regulatory signals at transcriptionally active subnuclear sites. Proc. Natl. Acad. Sci. USA *99*, 8048–8053.

Zang, C., Schones, D.E., Zeng, C., Cui, K., Zhao, K., and Peng, W. (2009). A clustering approach for identification of enriched domains from histone modification ChIP-Seq data. Bioinformatics *25*, 1952–1958.

Zawel, L., Dai, J.L., Buckhaults, P., Zhou, S., Kinzler, K.W., Vogelstein, B., and Kern, S.E. (1998). Human Smad3 and Smad4 are sequence-specific transcription activators. Mol. Cell *1*, 611–617.

Zhang, Y., Liu, T., Meyer, C.A., Eeckhoute, J., Johnson, D.S., Bernstein, B.E., Nusbaum, C., Myers, R.M., Brown, M., Li, W., and Liu, X.S. (2008). Model-based analysis of ChIP-Seq (MACS). Genome Biol. *9*, R137.

Cell Reports

Supplemental Information

Context-Specific Effects of TGF-β/SMAD3 in Cancer Are Modulated by the Epigenome

Ana Tufegdzic Vidakovic, Oscar M. Rueda, Stephin J. Vervoort, Ankita Sati Batra, Mae Akilina Goldgraben, Santiago Uribe-Lewis, Wendy Greenwood, Paul J. Coffer, Alejandra Bruna, and Carlos Caldas

Figure S1

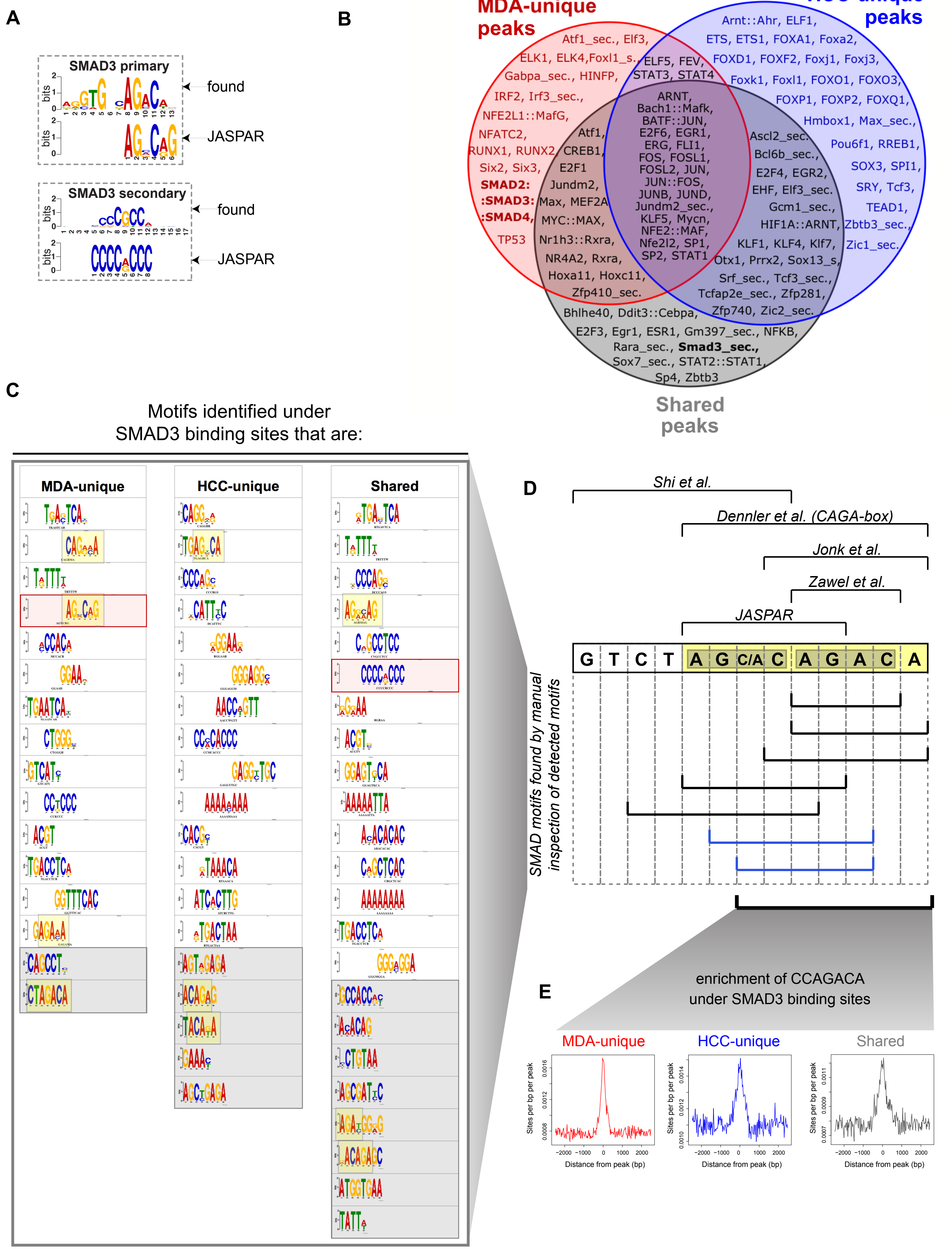

A
SMAD3 primary
found
JASPAR
SMAD3 secondary
found
JASPAR
B
MDA-unique peaks
HCC-unique peaks
Shared peaks
Atf1_sec., Elf3, ELK1, ELK4,Foxl1_s., Gabpa_sec., HINFP, IRF2, Irf3_sec., NFE2L1::MafG, NFATC2, RUNX1, RUNX2, Six2, Six3, SMAD2::SMAD3::SMAD4, TP53
ELF5, FEV, STAT3, STAT4
Arnt::Ahr, ELF1, ETS, ETS1, FOXA1, Foxa2, FOXD1, FOXF2, Foxj1, Foxj3, Foxk1, Foxl1, FOXO1, FOXO3, FOXP1, FOXP2, FOXQ1, Hmbox1, Max_sec., Pou6f1, RREB1, SOX3, SPI1, SRY, Tcf3, TEAD1, Zbtb3_sec., Zic1_sec.
ARNT, Bach1::Mafk, BATF::JUN, E2F6, EGR1, ERG, FLI1, FOS, FOSL1, FOSL2, JUN, JUN::FOS, JUNB, JUND, Jundm2_sec., KLF5, Mycn, NFE2::MAF, Nfe2l2, SP1, SP2, STAT1
Atf1, CREB1, E2F1, Jundm2, Max, MEF2A, MYC::MAX, Nr1h3::Rxra, NR4A2, Rxra, Hoxa11, Hoxc11, Zfp410_sec.
Ascl2_sec., Bcl6b_sec., E2F4, EGR2, EHF, Elf3_sec., Gcm1_sec., HIF1A::ARNT, KLF1, KLF4, Klf7, Otx1, Prrx2, Sox13_s, Srf_sec., Tcf3_sec., Tcfap2e_sec., Zfp281, Zfp740, Zic2_sec.
Bhlhe40, Ddit3::Cebpa, E2F3, Egr1, ESR1, Gm397_sec., NFKB, Rara_sec., Smad3_sec., Sox7_sec., STAT2::STAT1, Sp4, Zbtb3
C
Motifs identified under SMAD3 binding sites that are:
MDA-unique
HCC-unique
Shared
D
Shi et al.
Dennler et al. (CAGA-box)
Jonk et al.
Zawel et al.
JASPAR
G T C T A G C/A C A G A C A
SMAD motifs found by manual inspection of detected motifs
E
enrichment of CCAGACA under SMAD3 binding sites
MDA-unique
HCC-unique
Shared
Sites per bp per peak
Distance from peak (bp)

Figure S2

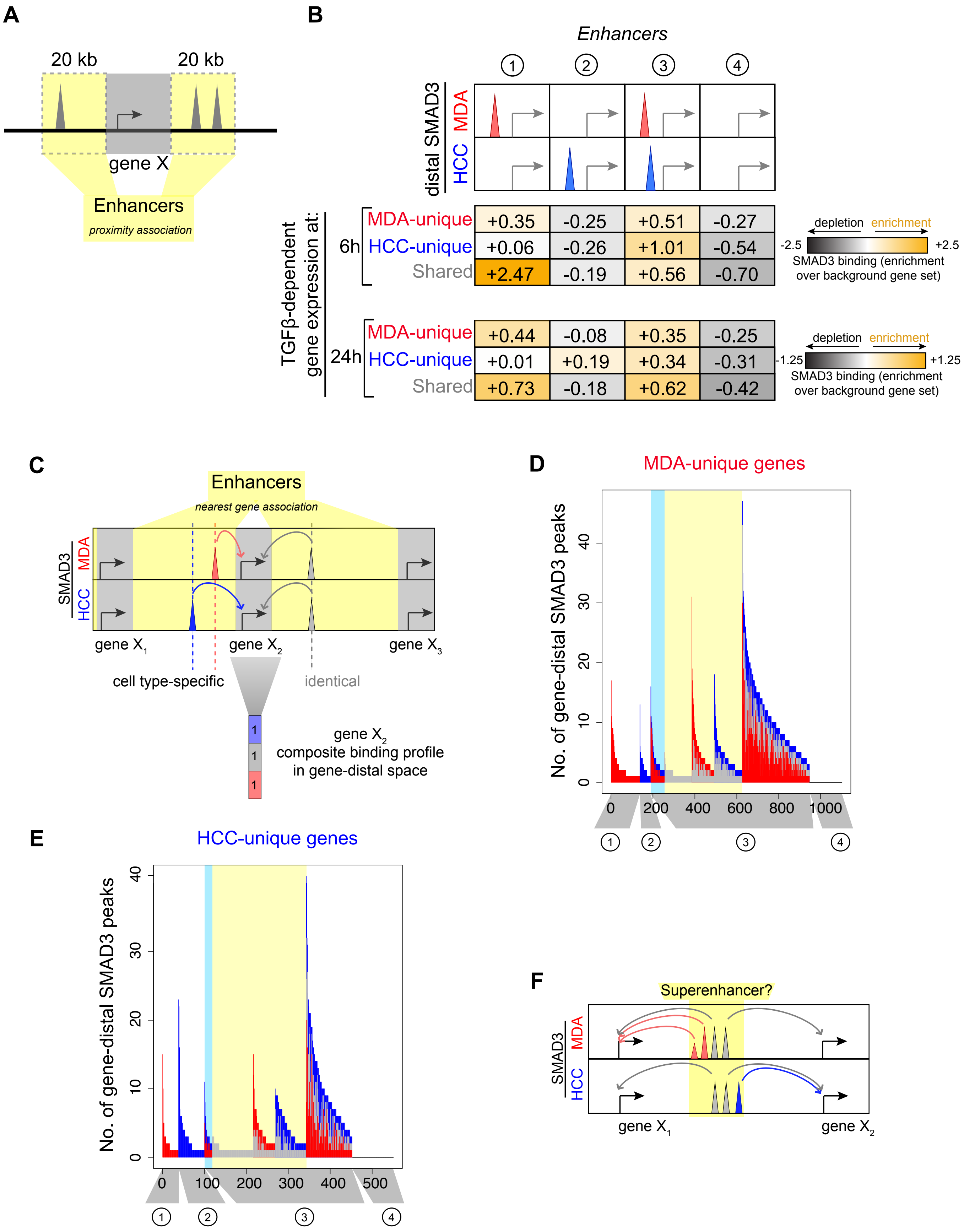
A
20 kb
20 kb
gene X
Enhancers
proximity association
B
Enhancers
1
2
3
4
distal SMAD3
MDA
HCC
TGFβ-dependent gene expression at:
6h
MDA-unique
HCC-unique
Shared
+0.35
-0.25
+0.51
-0.27
+0.06
-0.26
+1.01
-0.54
+2.47
-0.19
+0.56
-0.70
24h
+0.44
-0.08
+0.35
-0.25
+0.01
+0.19
+0.34
-0.31
+0.73
-0.18
+0.62
-0.42
depletion
enrichment
-2.5
+2.5
-1.25
+1.25
SMAD3 binding (enrichment over background gene set)
C
Enhancers
nearest gene association
SMAD3
gene X1
gene X2
gene X3
cell type-specific
identical
gene X2 composite binding profile in gene-distal space
D
MDA-unique genes
No. of gene-distal SMAD3 peaks
E
HCC-unique genes
F
Superenhancer?

Figure S3

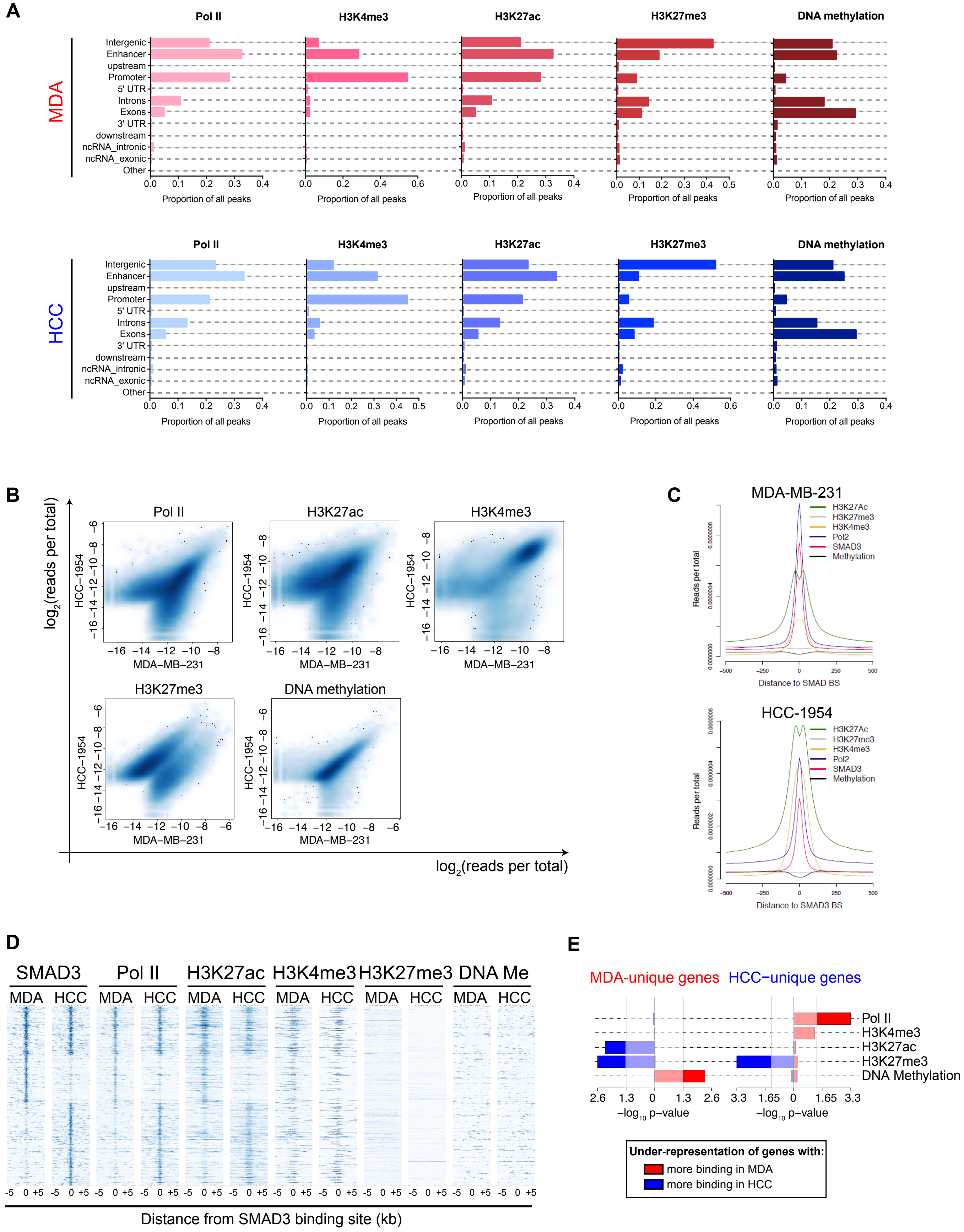

A
Pol II
H3K4me3
H3K27ac
H3K27me3
DNA methylation
MDA
HCC
Intergenic
Enhancer
upstream
Promoter
5' UTR
Introns
Exons
3' UTR
downstream
ncRNA_intronic
ncRNA_exonic
Other
Proportion of all peaks
B
log2(reads per total)
HCC−1954
MDA−MB−231
C
MDA-MB-231
HCC-1954
H3K27Ac
H3K27me3
H3K4me3
Pol2
SMAD3
Methylation
Reads per total
Distance to SMAD BS
Distance to SMAD3 BS
D
SMAD3
Pol II
H3K27ac
H3K4me3
H3K27me3
DNA Me
MDA
HCC
Distance from SMAD3 binding site (kb)
E
MDA-unique genes
HCC−unique genes
Pol II
H3K4me3
H3K27ac
H3K27me3
DNA Methylation
−log10 p−value
Under-representation of genes with:
more binding in MDA
more binding in HCC

Figure S4

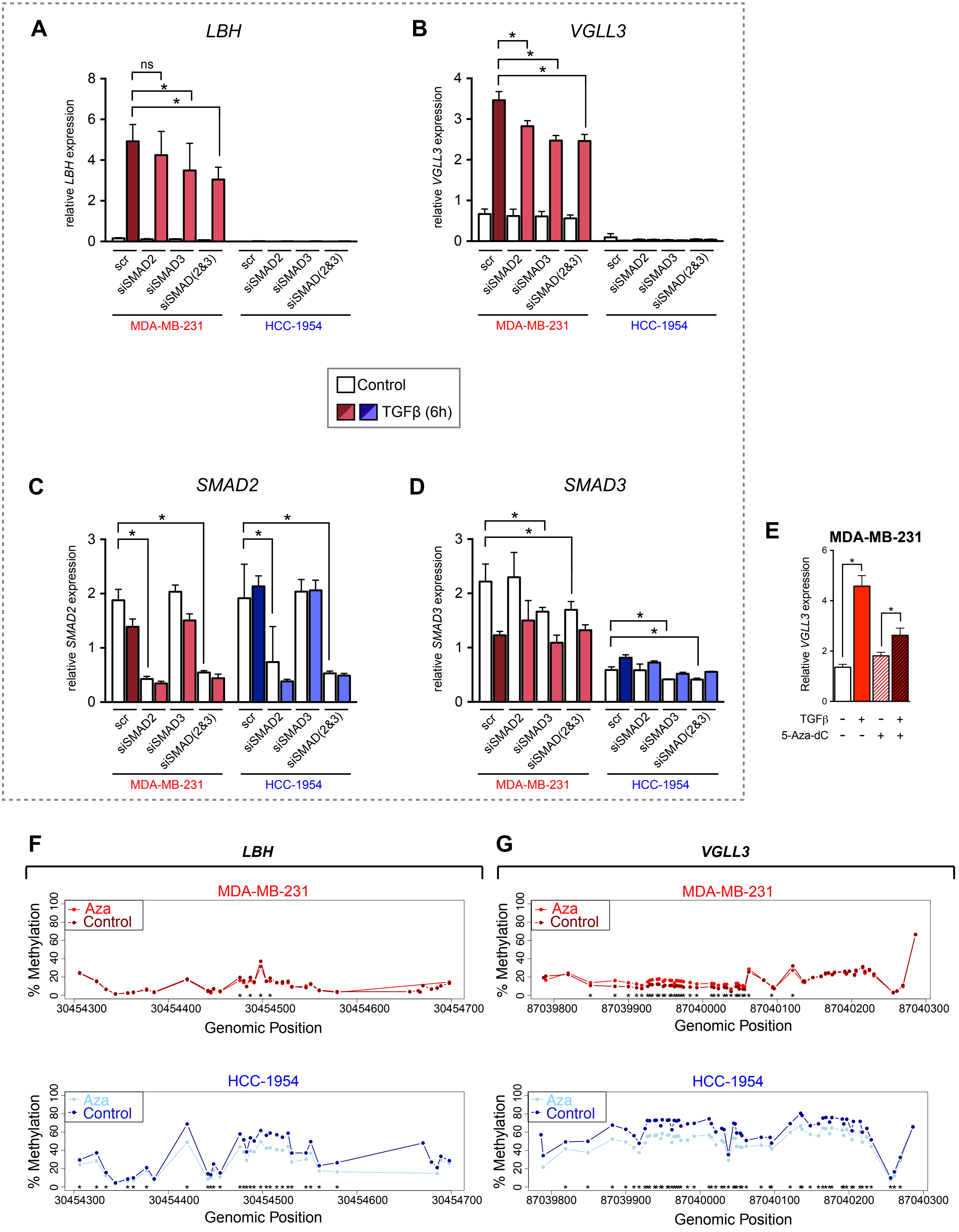
A
LBH
relative LBH expression
ns
scr
siSMAD2
siSMAD3
siSMAD(2&3)
MDA-MB-231
HCC-1954
B
VGLL3
relative VGLL3 expression
Control
TGFβ (6h)
C
SMAD2
relative SMAD2 expression
D
SMAD3
relative SMAD3 expression
E
MDA-MB-231
Relative VGLL3 expression
TGFβ - + - +
5-Aza-dC - - + +
F
LBH
G
VGLL3
% Methylation
Aza
Control
Genomic Position
30454300
30454400
30454500
30454600
30454700
87039800
87039900
87040000
87040100
87040200
87040300

Figure S5

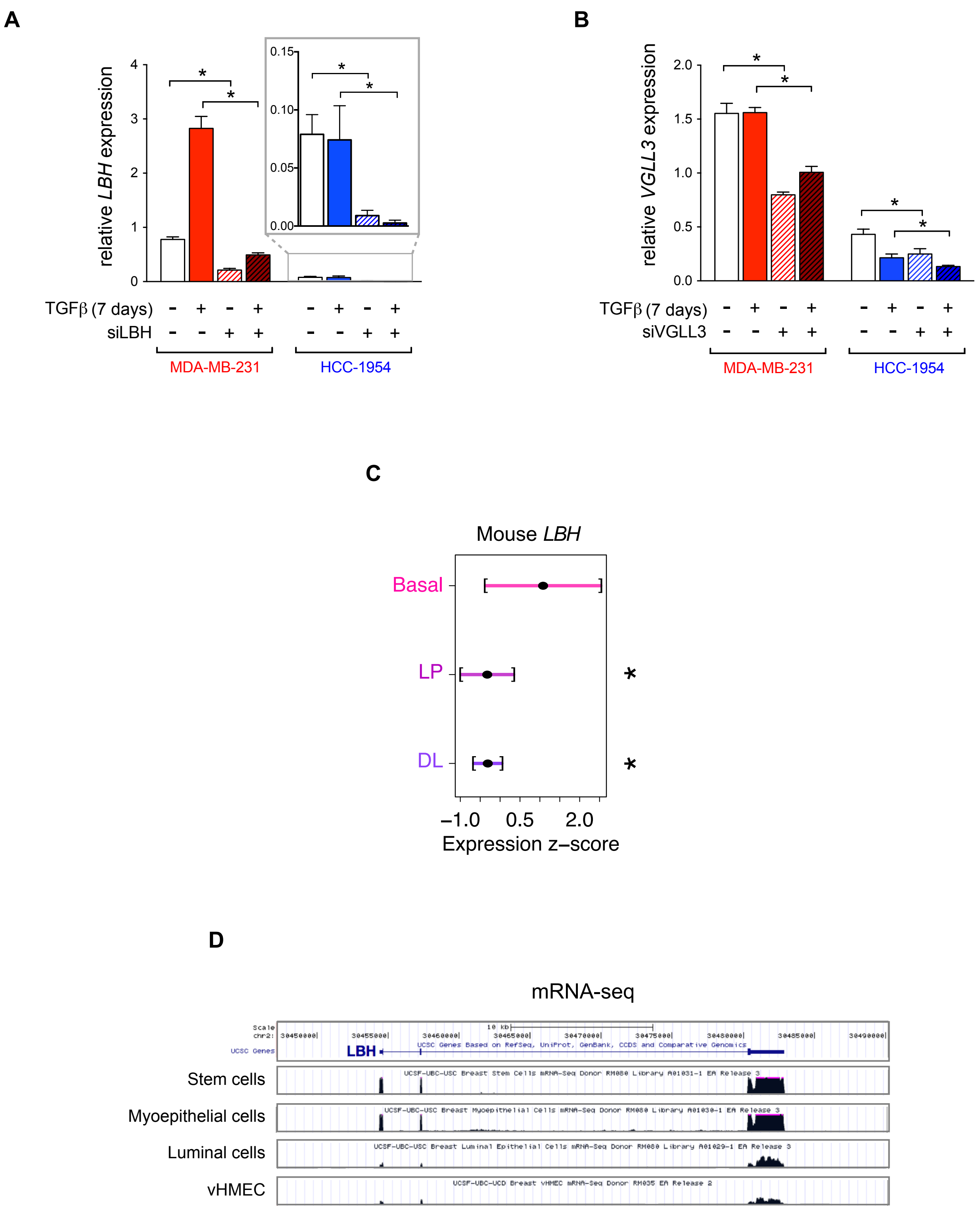
A
relative LBH expression
0
1
2
3
4
*
*
0.00
0.05
0.10
0.15
TGFβ (7 days) - + - + - + - +
siLBH - - + + - - + +
MDA-MB-231
HCC-1954
B
relative VGLL3 expression
0.0
0.5
1.0
1.5
2.0
TGFβ (7 days) - + - + - + - +
siVGLL3 - - + + - - + +
MDA-MB-231
HCC-1954
C
Mouse LBH
Basal
LP
DL
*
*
−1.0
0.5
2.0
Expression z−score
D
mRNA-seq
Scale
chr2:
10 kb
UCSC Genes
LBH
UCSC Genes Based on RefSeq, UniProt, GenBank, CCDS and Comparative Genomics
Stem cells
Myoepithelial cells
Luminal cells
vHMEC

Supplemental Figure Legends

Figure S1. SMAD3 Binds to Multiple Diverse DNA Motifs in BTICs. Related to Figure 2.

(A) Identification of SMAD3 motifs in the SMAD3 ChIP-seq datasets. Motif analysis was performed in MEME.

(B) Putative SMAD3 partner TFs corresponding to DNA motifs detected in C (see below). Each circle represents putative co-factors (identified in MEME) whose motifs were found under MDA-unique (red), HCC-unique (blue) or Shared (grey) SMAD3 peaks. Note that even cell type-unique SMAD3 binding sites (peaks) can possess similar or identical DNA motifs, to which then identical TFs could bind, resulting in the overlap of the circles.

(C) Distinct DNA motifs detected under MDA-MB-231-unique SMAD3 binding sites, HCC-1954-unique SMAD3 binding sites and shared SMAD3 binding sites. Red boxes indicate primary and secondary SMAD3 motifs identified by MEME, yellow boxes mark manually identified SMAD-binding elements, whereas grey boxes indicate motifs unknown to associate with any factors to date. Motif enrichment analysis was performed in MEME. All motifs were significantly enriched in the corresponding datasets (e-value < 0.05).

(D) Manually identified SMAD motifs from (C). The nucleotide sequence shows experimentally determined SMAD binding sequence context and the corresponding studies are indicated above the sequence bar. CAGA-box is highlighted in yellow. Manually detected SMAD binding motifs are labelled below the sequence bar. All motifs that contain an uninterrupted AGAC sequence are marked with black bars.

(E) Enrichment analysis of the CCAGACA SMAD binding motif under SMAD3 binding sites unique to MDA, unique to HCC and those shared between BTICs. Analysis was performed in HOMER (See Supplemental Experimental Procedures for details).

Figure S2. SMAD3 Binding Patterns in the Gene Distal Space. Related to Figure 2.

(A) Sketch of the analysis approach used to define SMAD3-bound enhancers by proximity association. All SMAD3 peaks falling within 20 kb up- and downstream of the gene boundaries were annotated to that gene.

(B) Genome-wide analysis showing the enrichment of each of the four binding modes of SMAD3-bound distal enhancers on TGFβ-dependent genes. Gene expression data from 6h and 24h time-points were used. Enrichment was calculated over SMAD3 binding distribution in the TGFβ-independent, background gene set. Note that the common binding mode (3) does not exclude SMAD3 binding sites that are annotated to the same genes but occur on different sites in the two BTICs.

(C) Schematic of the nearest gene association approach. Each SMAD3 peak was associated with the nearest gene, excluding SMAD3 peaks in the gene-proximal space. For each TGFβ-dependent gene the number of context-specific SMAD3 binding sites (red and blue) and shared binding sites (grey) were then calculated and represented as a composite profile.

(D) and (E) Analysis of the SMAD3-bound enhancer profiles associated with context-specific TGFβ-dependent genes (performed as outlined in C). TGFβ-dependent genes 24h post-TGFβ stimulation were used. Genes are aligned along the x-axis, and grouped into distinct categories based on their SMAD3 composite profiles. SMAD3 binding modes are indicated below the plot in grey. Light blue box marks genes with mutually exclusive SMAD3 binding patterns, and yellow those with predominantly similar or identical SMAD3 binding patterns in both BTICs.

(F) Sketch of the potential limitations of the nearest gene association approach, where peaks that functionally represent the same regulatory element - a “superenhancer” (Whyte et al., 2013) might be annotated to different genes.

Figure S3. MDA-MB-231 and HCC-1954 BTICs Harbour Global Epigenetic Differences. Related to Figure 3.

(A) Genomic annotation of the profiled chromatin factors. Peak annotation was performed in annovar and promoter overlap was computed using the validated promoter list from Weber et al., 2007.

(B) Density plots showing the pair-wise comparison of the intensity of detected peaks in MDA-MB-231 and HCC-1954 BTICs. Data are represented as the $\log_2$ of the reads per total.

(C) Average binding profiles of each of the factors around SMAD3 binding sites, in a genomic window of 1 kb.

(D) Maps showing the occupancy of Pol II, H3K27ac, H3K4me3, H3K27me3 and DNA methylation around genomic SMAD3 binding sites, in the windows of 10 kb. SMAD3 peaks are grouped into three categories (top to bottom): peaks shared between both BTICs, peaks unique MDA-MB-231 BTICs and peaks unique to HCC-1954 BTICs.

(E) Gene set enrichment analysis showing the under-representation of genes with differential levels of Pol II and epigenetic modifications between BTICs within the MDA-unique (left) and HCC-unique (right) gene sets. The significance of under-representation is shown as a p-value on a bi-symmetrical x-axis, the left and the right sides of the axis corresponding to the depletion of genes with more binding of the corresponding mark in HCC-1954 (blue) and MDA-MB-231 (red), respectively. p-value cut-offs were set at 0.05 ($-\log_{10}(0.05) = 1.30$) (dashed lines). Analysis was performed as in Figure 3A. See Supplemental Experimental Procedures for details.

Figure S4. TGFβ-dependent Regulation of *LBH* and *VGLL3* Depends on SMAD2/3 and DNA Methylation. Related to Figure 5.

(A), (B), (C) and (D) RT-qPCRs measuring the levels of *LBH* (A), *VGLL3* (B), *SMAD2* (C) and *SMAD3* (D) transcripts upon siRNA-mediated knock-down of *SMAD2* alone, *SMAD3* alone, or *SMAD2* and *SMAD3* in combination. The cells were transfected upon seeding, allowed to form mammospheres for 7 days, and then treated with TGFβ for 6h (6h time point was chosen in order to assess the effects of SMAD2/3 depletion on *LBH* and *VGLL3* simultaneously, as *LBH* is induced by TGFβ at 3h of treatment and *VGLL3* at 6h of treatment). Gene expression was normalised to the housekeeping (*RBM22*) transcript levels. Mean of three biological replicates with SD is shown, asterisks indicate significant differences, ns = not significant (two-way ANOVA).

(E) RT-qPCR showing the expression of *VGLL3* transcript upon 5-aza-dC and TGFβ treatment in MDA-MB-231 BTICs. The samples used are identical to those used in the experiments on Figure 5D. The data were normalised to the housekeeping *PSMC4* transcript levels, and presented as mean ± standard deviation (SD), asterisks indicate significant differences (two-tailed t-test). The purpose of this experiment was to ensure that the diminished *VGLL3* induction in MDA-MB-231 upon 5-aza-dC treatment is not a result of a technical problem or 5-aza-dC affecting the expression of the *RBM22* housekeeper.

(F) and (G) Targeted bisufite sequencing measuring DNA methylation levels across *LBH* (F) and *VGLL3* (G) promoters, in MDA-MB-231 (top) and HCC-1954 (bottom) BTICs, upon treatment of cells with 5-aza-dC. Asterisks indicate significant differences ($p<0.1$) as determined by FDR-corrected logistic regression test.

Figure S5. *LBH* and *VGLL3* Transcript Levels Are Depleted by siRNA-mediated Knock-down and Change with the Developmental Status. Related to Figure 6.

(A) and (B) RT-qPCRs measuring *LBH* and *VGLL3* transcript levels, respectively, upon siRNA-mediated knockdown of *LBH* and *VGLL3*. The cells were treated with TGFβ and transfected with siRNAs at the moment of seeding, and then allowed to form mammospheres for 7 days. Gene expression was normalised to the housekeeping (*PSMC4*) transcript levels. Mean of three replicates with SD is shown, asterisks indicate significant differences (one-way ANOVA).

(C) *LBH* transcript expression in different cell compartments of the mouse mammary gland. Basal compartment, luminal progenitors (LP) and differentiated luminal cells (DL) are shown. Significance was determined by a linear model (ANOVA) comparing LP and DL expression to the basal group. Data from Shehata et al., 2012.

(D) Genome browser screenshots showing transcript expression (mRNA-seq) signals over the *LBH* locus in distinct cell types of the normal mammary gland and in the variant human mammary epithelial (vHMEC) cells. Publicly available data were obtained from the Roadmap Epigenomics project (Kundaje et al., 2015). All tracks are presented on the same scale for all the samples (not shown for simplicity).

Supplemental Tables

Table S1. Dynamics of TGFβ-mediated Gene Expression in BTICs. Related to Figure 1.

An Excel file providing the lists of TGFβ-dependent genes at four different time points (1h, 3h, 6h and 24h) upon pathway induction, in MDA-MB-231 and HCC-1954 BTICs.

Table S2. SMAD3-high, Open Chromatin-high and DNA Hypo-methylation Gene Sets. Related to Figure 3.

An Excel file providing the lists of genes in the SMAD3-high, open chromatin-high and DNA hypo-methylation sets for each BTIC type.

Supplemental Experimental Procedures

Cell Propagation in Adherent Cultures

Breast cancer cell lines were first grown as adherent cultures for the purpose of propagation. MDA-MB-231 was grown in DMEM supplemented with 10% fetal bovine serum (FBS) and 100 U/ml penicillin-streptomycin (Life Technologies), and HCC-1954 was grown in RPMI supplemented with 10% FBS and 100 U/ml penicillin-streptomycin. When reaching around 80% confluence, the cells were washed with PBS, then collected and singularised using 0.05% trypsin (Invitrogen). Trypsin was inactivated with the supplemented DMEM or RPMI media and the cells were collected and washed twice with PBS. After washing and for the purpose of seeding mammosphere cultures, the cells were re-suspended in an appropriate volume of DMEM-F12 media supplemented with 0.1 x B27, 20 ng/μl fibroblast growth factor (FGF), 20 ng/μl epidermal growth factor (EGF) and 100 U/ml penicillin-streptomycin.

Mammosphere Cultures

Mammosphere cultures were seeded at 1×10^5 cells/ml density. Mammospheres were grown in DMEM-F12 media supplemented with 0.1 x B27, 20 ng/μl FGF, 20 ng/μl EGF and 100 U/ml penicillin-streptomycin in ultra-low attachment plates (Corning).

TGFβ Pathway Manipulation

To stimulate the TGFβ pathway recombinant TGFβ1 protein (R&D Systems) was used at a final concentration of 0.1 nM (of the protein dimer). Duration of the stimulus is indicated in each experiment.

5-Aza-2'-deoxycytidine Treatment

To reduce global levels of DNA methylation, attached cells prior to mammosphere seeding were treated with 5-aza-2'-deoxycytidine (Sigma-Aldrich) at 1 μM final concentration during two

consecutive passages. The third spike-in of 5-aza-2'-deoxycytidine was added immediately after seeding mammospheres. The spheres were grown for 7 days and then treated with TGFβ for 24h. Mammospheres were collected, washed twice with PBS and then each sample was split in two, one half for DNA extraction and one half for RNA extraction. For RNA extraction, pellets were lysed in Qiazol and RNA was extracted using miRNeasy kit (Qiagen). DNA was extracted using phenol-chloroform extraction and ethanol precipitation.

siRNA-mediated Knock-down Experiments

To knock-down *LBH*, *VGLL3*, *SMAD2* and *SMAD3* in mammospheres, for each gene pools of 4 targeting siRNAs were used (GE Healthcare) at 25 nM final concentration. Non-targeting siRNA (GE Healthcare, D-001810-01-20) was used as a control. The cells were transfected immediately after seeding mammospheres at 1×10^5 cells/ml density and Dharmafect I (GE Healthcare) was used as the transfection reagent, according to the manufacturer's protocol. DMEM-F12 medium with 0.1 x B27, 20 ng/μl FGF, 20 ng/μl EGF but without any antibiotics was used for the whole duration of the experiment. In experiments with *LBH* and *VGLL3* knock-downs, TGFβ was added to the cells at 0.1 nM final concentration two hours after transfection, and mammospheres were then allowed to form for 7 days. In *SMAD2* and *SMAD3* knock-down experiments, the mammospheres were allowed to form for 7 days, and TGFβ pathway was then activated for 6h by addition of exogenous TGFβ at 0.1 nM final concentration. RNA was extracted using miRNeasy kit (Qiagen).

MS-IC and CFC Assays

To assess modulation of self-renewal and proliferation capacity induced by *LBH* and *VGLL3* knock-downs and TGFβ treatment, mammosphere initiating cell (MS-IC) assays and colony forming cell (CFC) assays were performed in parallel. siRNA transfections were performed as outlined above.

Seven days old mammospheres were span down at 1300 g and washed with PBS once. To obtain single cells, 1 ml of 0.05% trypsin was added to mammosphere pellets followed by incubation at 37°C for 2 minutes. Cells were then singularized by gentle pipetting. Trypsin was inactivated with 1 μl of 1000 x Trypsin Inhibitor (Roche) and diluted in 10 ml of PBS. Cells were centrifuged and the pellet was resuspended in an appropriate volume of DMEM-F12 media supplemented with 0.1x B27, 20 ng/μl FGF, 20 ng/μl EGF and 100 U/ml penicillin-streptomycin, to yield required dilutions of cells (1:2 for MDA-MB-231 and 1:4 for HCC-1954). Second generation spheres were seeded in ultra low attachment 96-well plates (Corning).

To image and count the number of mammospheres, a colorimetric assay was performed, where live cells were labeled on the 6th day from seeding with 1X tetrazolium dye 3-(4,5-dimethylthiazol-2-yl)-2,5-diphenyltetrazolium bromide (MTT), allowed to incorporate the dye over night, and imaged the following day on the Gel Count scanner (Oxford Optronix). Spheres were counted using automated Gel Count software. Data was analyzed with Prism 6.0 (GraphPad Software) and statistical significance was determined by ANOVA.

CFC assay was performed in parallel with second generation mammosphere assay. Singularized cells were seeded in 6 cm round collagen coated dishes (Fisher Scientific) at cell line specific densities (1:120 for MDA-MB-231 and 1:80 for HCC-1954), in 3 ml DMEM-F12 media supplemented with 5% FBS, 20 ng/μl FGF and 100 U/ml penicillin-streptomycin. The growth of colonies was monitored daily, and when reaching the appropriate density, all plates were washed twice with PBS, then fixed with methanol:acetone (1:1) for 30 s. The plates were then stained with 1:10 diluted Giemsa dye for 30 min, washed twice with PBS, allowed to air dry, and imaged on the Gel Count scanner (Oxford Optronix).

Targeted Bisulfite Sequencing

200 ng of DNA from 12 samples (3 biological replicates of each of the following conditions: MDA-MB-231 Control, MDA-MB-231 treated with 5-aza-dC, HCC-1954 Control and HCC-1954 treated with 5-aza-dC), were bisulfite converted and then column-purified using DNA methylation Gold kit (Zymo Research). This converted DNA template was used to amplify 10 regions of interest (spanning *LBH* and *VGLL3* promoters) using PfuTurbo Cx Hotstart DNA Polymerase (Agilent Technologies) (50 μl reaction: 5 μl 10X Pfu Turbo Cx Buffer, 1.25 μl dNTPs (10 mM each), 5 μl F+R primer mix (2.5

μM each), 1 μl PfuTurbo Hotstart Cx Polymerase, water up to 50 μl) in 40 PCR cycles. Primers were designed in Bisulfite Primer Seeker (Zymo Research), and for each primer pair the annealing temperatures were first optimised by testing temperature gradients (data not shown). The following 10 PCR primer pairs were used (when used across 12 samples giving a total of 120 PCR reactions):

	Region	Forward Primer	Reverse Primer	T(°C)
1.	LBH_1	TTATAGGGGYGTGTGTTAG TTTGTTTTAGG	AATTCACACRTAACCCCTA ACTCCCCC	59.8
2.	LBH_2	GAGYGTTGAAGTTATTTAT GATTTTGG	AATATATAAACATAAAATC CTAAAACAAACTAACACA C	54.2
3.	LBH_3	TTGGTGAYGTTATTTTAGG AGTGGG	AATATATAAACATAAAATC CTAAAACAAACTAACACA C	54.2
4.	LBH_4	AGGGGTTAYGTGTGAATTT TTTTTAATG	CCAATCCCRACCCCCACCA ATATAC	55.5
5.	LBH_5	GGTGGGGGGAGGGGGTGT TGAGAATATTTAGATAAA G	CACCCCCCCRAAACTCTAC AAACCCTAC	63.4
6.	LBH_6	GGAGAAGAYGTGGGAGTT AAGGATGGGG	CCCTCTAAAACRTTTATTC CCCATACTAAACCTCTCTC	63.1
7.	VGLL3_1	GTYGGTTGATAGTAGGTTG TGGGGTAGGTTG	CAAAAAACATCCRAAAAA ACTAAAAATAAAAATACC	55.5
8.	VGLL3_2	GGTAGGTTGTYGTTGTTAT GGGGTTGGGTAGATATTG	CAAAAAACATCCRAAAAA ACTAAAAATAAAAATACC	55.5
9.	VGLL3_3	GTTYGGTGATTTATTTGTT GGTTAGGTTGGGG	TAAACCCCCRCTAATTACC AATCCCTCCC	61.5
10.	VGLL3_4	GGGGTYGGGAGGGATTGG TAATTAG	TACAAACCRAAACTAAACT CCCCAC	56.9

PCR products were purified using 2X volume of solid phase reversible immobilisation (SPRI) beads (Illumina), two 80% ethanol washes and eluted in 25 μl of 10mM Tris-HCl, pH 8.5. Amplicons were then end-repaired and A-tailed in 30 μl reactions (1 μl of Klenow 5'->3' exo- (NEB), 3 μl of 10X NEB2 buffer, 1 μl of the dNTP solution (1mM dCTP, 1mM dGTP, 1mM dTTP and 10 mM ATP (in excess for A-tailing))), by incubation at 30°C for 20 min and 37°C for 20 min. Amplicons were purified from this reaction by addition of 2X volume of SPRI as above however at the last step the beads were retained in the 20 μl 10mM Tris-HCl, pH 8.5 elute and carried over through the subsequent reaction.

Amplicons were then ligated to barcoded methylated DNA adapters (TruSeq LT, Illumina) by adding 2 μl of 1:20 diluted TruSeq Illumina adapters, and then the master mix containing 1 μl T4 DNA Ligase (400,000 U/ml) (NEB), 3 μl T4 DNA Ligase buffer (NEB) and 4 μl nuclease-free water, giving the total 30 μl ligation reaction per sample. Barcoding was performed in such way that all amplicons from the same sample harbour one, same barcode. Ligation was carried out over night at 16°C and the following day it was inactivated by incubation at 65°C for 20 min. Ligation products were purified by

addition of double volume of PEG-NaCl solution (20% w/v PEG 8000, 2.5M NaCl) to the bead-containing ligation reaction, two 80% ethanol washes and elution in 10 µl of 10mM Tris-HCl, pH 8.5.

After purifying these 120 barcoded amplicons the quantity of each one was assessed using qPCR (KAPA Biosystems). These measurements were used to normalize sample amounts for pooling: amplicons representing the same region (e.g. LBH_1) from 12 different samples were pooled in equimolar ratios, giving rise to 10 pools, each representing a different genomic region. These 10 pools were then combined (without normalizing their amounts across each other, as some of them had very low yields), and purified with 2X volume of SPRI, two 80% ethanol washes and elution in 100 µl of 10mM Tris-HCl, pH 8.5, in order to bring down the volume of the solution.

This pooled library was then amplified with PfuTurbo Cx Hotstart DNA Polymerase (Agilent Technologies) in 14 PCR cycles (reaction conditions identical as above apart from the reaction volume which was now 200 µl). The amplified library was then purified with two consecutive SPRI purifications, one with 1.2X bead-to-sample volume ratio, and another with 1.5X bead-to-sample volume ratio, and both using two 80% ethanol washes and elution in 10mM Tris-HCl, pH 8.5. A small aliquot of the final library was taken and diluted for quality controls (1:10 dilution for Bioanalyser HS and 1:4,000 for qPCR (KAPA Biosystems)).

Sequencing was performed on MiSeq (Illumina) using 150 bp paired end sequencing (CRUK CI Genomics Core). Quality control and trimming was performed with FastQC and trim galore (Andrews, 2010) and reads were aligned to the Human Genome Build 37 (hg19) using Bismark (Krueger and Andrews, 2011). Methylation of individual CpG sites was called using Bismark too. Downstream data analysis was performed in R and detection of differentially methylated CpGs was done fitting a logistic regression model to each CpG and correcting the p-values using FDR with a threshold of 0.1.

Reverse Transcription Quantitative PCR (RT-qPCR)

To generate cDNA, 100-400 ng of RNA per sample was mixed with 1µl of 50 µM custom made Oligo-$(dT)_{16}$ (5'-$d(T)_{16}$VN-3', V= dA or dG or dC; N = dA or dG or dC or dT), and denatured at 65°C for 5 min. The samples were snap cooled on ice and reverse transcription was performed with Transcriptor First Strand cDNA Synthesis Kit (Roche), as recommended by the manufacturer. The samples were incubated at 25°C for 10 min, 55°C for 30 min and 85°C for 5 min.

Generated cDNAs were diluted with nuclease-free water in 1:10 ratio, and qPCR was performed using the TaqMan chemistry. Gene-specific TaqMan probes and TaqMan Fast Universal PCR Master Mix (Applied Biosystems) were used, as recommended by the manufacturer. The plates were run on Applied Biosystems 7900HT Fast Real-Time PCR system, with the fast cycling conditions (20s of denaturation at 95°C, followed by 40 cycles of 95°C for 1s and 60°C for 20s). Data were analyzed in SDS 2.4 software (Applied Biosystems), Excel (Microsoft) and Prism 6.0 (GraphPad Software). Statistical significance was determined using ANOVA.

Gene Expression Profiling using Illumna HumanHT-12 BeadChips

Gene expression analysis was performed on HumanHT-12 Expression BeadChips from Illumina, using 200 ng of RNA as a starting material. For each condition, biological triplicates were used.

Gene Expression Data Analysis

Gene expression data were analyzed with the beadarray package (Dunning et al., 2007). First, quality assessment was performed. Then, spatial artifacts were removed using BASH (Cairns et al., 2008), and probes were summarized and quantile normalized. Probe re-annotation was done using the IlluminaHumanv4.db package (Barbosa-Morais et al., 2010) and only probes that were a perfect match to their target were kept. A linear model comparing the expression of TGFβ-treated cells vs non-treated cells within each replicate was fit using the limma package (Smyth, 2005) and lists of differentially expressed genes for each cell line were obtained using a threshold of 0.1 FDR.

Chromatin Immunoprecipitation and Sequencing

Roughly 7×10^7 cells per condition at 1×10^5 cells/ml density were seeded as mammosphere cultures for SMAD3 ChIPs. For all other ChIPs (Pol II and histone modifications) 2×10^7 cells were used per condition. Due to the large scale of the experiment, cells were grown in 500 ml volume units in low attachment spinner flasks (Corning) that provide constant mixing thereby preventing aggregation of cells. Mammospheres were allowed to form for 7 days. For SMAD3 experiments only the cells were treated with TGFβ at 0.1 nM final concentration for 3h. For all other experiment untreated mammospheres were used.

Mammospheres were aliquoted in 50 ml falcon tubes, centrifuged and washed 3 x with PBS. Following the final wash, mammospheres were crosslinked in 30 ml of 1.66 mM Di(N-succinimidyl) glutarate (DSG) solution (dissolved in PBS) per condition, and incubated for 45 min at room temperature on the turning wheels. Mammospheres were washed 3 x with PBS and resuspended in 20 ml of PBS. 2 ml of freshly prepared formaldehyde solution (50 mM Hepes-KOH pH 7.5, 100 mM NaCl, 1 mM EDTA, 0.5 mM EGTA, 11% (v/v) formaldehyde) were then added to each sample and the samples were incubated for 30 min at room temperature with tumbling. The reaction was then quenched with 1/10 of the volume of 1.25 M glycine, incubated at room temperature for 5 min, and crosslinked mammospheres were washed 2 times with ice-cold PBS.

To remove cytosol and extract nuclei, mammosphere pellets were washed three times for 5 min with Nuclear extraction buffer (20 mM Tris-HCl pH 8.0, 10 mM NaCl, 2 mM EDTA, 0.5% (v/v) Igepal CA630, 1x protease inhibitor cocktail (PIC, Roche)). From this point, all centrifugation steps were carried out at 4°C. The pellets were then re-suspended in 2 ml of Sonication buffer (20 mM Tris-HCl pH 7.5, 150 mM NaCl, 2mM EDTA, 1% (v/v) Igepal CA630, 0.3% (v/v) sodium dodecyl sulfate (SDS), 1x PIC) and transferred to 5 ml Covaris tubes (LGC Genomics). Chromatin shearing was performed on Covaris S-220, for the total duration of 8-9 min.

To remove debris, samples were transferred to 15 ml Falcon tubes and centrifuged at maximum speed (4,000 g) at 4°C for 20 min. The supernatants were transferred to new tubes containing 1 volume (2 ml) of the ChIP dilution buffer (20 mM Tris-HCl pH 8.0, 150 mM NaCl, 2 mM EDTA, 1% (v/v) Triton X-100, 1x PIC). At this point 1% of each sample volume was set aside to serve as an input, and stored at -20°C until further processing.

To each sample, 45 μl of 10 % bovine serum albumin (BSA), 0.5 ml of the pre-blocked Protein G Agarose beads (Santa Cruiz, 60 μl of beads of stock per condition, washed twice and then blocked for several hours in 500 μl ChIP buffer containing 0.1 % (w/v) BSA) and 10 μg of the corresponding antibody were added. The following antibodies were used: anti-SMAD3 (ab28379), anti-RNA Pol II (ab5408), anti-H3K4me3 (Millipore, 05-1339), anti-H3K27me3 (Millipore, 07-449) and anti-H3K27ac (ab4729). Immunoprecipitation was carried over night at 4°C with tumbling.

The beads were then span down at 40 g for 5 min at 4°C, the supernatant containing the unbound chromatin fraction was removed, the beads were re-suspended in 1 ml of ChIP buffer (20 mM Tris-HCl pH 8.0, 150 mM NaCl, 2 mM EDTA, 1% (v/v) Triton X-100, 0.15% (w/v) SDS, 1x PIC) and transferred to DNA LoBind tubes (Eppendorf). This was followed by one wash with Wash buffer 2 (20 mM Tris-HCl pH 8.0, 500 mM NaCl, 2 mM EDTA, 1% (v/v) Triton X-100, 0.1 % (w/v) SDS, 1x PIC), one wash with Wash buffer 3 (20 mM Tris-HCl pH 8.0, 250 mM LiCl, 2 mM EDTA, 0.5 % (v/v) Igepal CA630, 0.5 % (w/v) sodium deoxycholate, 1x PIC) and two washes with TE buffer (10 mM Tris-HCl pH 7.4, 1 mM EDTA), all at 4°C.

After the final wash the beads were re-suspended in 250 μl of freshly prepared, pre-warmed (37°C) Elution buffer (100 mM $NaHCO_3$, 1% (w/v) SDS) and incubated with shaking at room temperature for

15 min. The beads were span down at 1,000 rpm and the supernatant fraction (elute) was saved in a new DNA LoBind tube. Another 250 μl of pre-warmed (37°C) Elution buffer were then added to the beads and the second elution was performed with shaking at room temperature for 15 min. The beads were then span down, and the supernatant (second elute) was combined with the first one. 500 μl of the combined elute was then also span down to eliminate any remaining agarose beads, and the supernatant (480 μl) was transferred to a new DNA LoBind tube.

To reverse the crosslink between the DNA and proteins, 20 μl of 5 M NaCl was added to each 480 μl elute (for immunoprecipitated samples) and to input samples which were first set to 480 μl by adding water. These reactions were incubated at 65°C over night. The following day, to digest proteins, 20 μl of 1M Tris-HCl pH 8.0, 10 μl of 0.5 M EDTA and 5 μl of 10 mg/ml Proteinase K were added to each 500 μl sample, and the mixtures were incubated at 55°C for 1h. DNA was purified from this reaction by phenol-chloroform extraction and overnight ethanol precipitation (with addition of glycogen or glycoblue as carriers) at -20°C.

Libraries for Illumina sequencing were prepared with TruSeq LT ChIP kit, as recommended by the manufacturer. Size selection of the DNA libraries was performed on 2% TAE agarose gels, selecting for fragments in the size range 200-600 bp. Sequencing was performed on HiSeq 2000 (Illumina), with 40 bp single end reads. For each of the marks, multiplexed pools of 12 samples were sequenced on multiple lanes, to meet the following coverage criteria: SMAD3 – 30 x 10^6 reads per sample, Pol II - 60 x 10^6 reads per sample, H3K27ac – 30 x 10^6 reads per sample, H3K4me3 – 30 x 10^6 reads per sample, H3K27me3 – 45 x 10^6 reads per sample.

ChIP-seq Data Analysis

Briefly, bases were called and the general quality of the sequencing run was assessed by the FastQC pipeline (Andrews, 2010). The reads were then filtered based on sequencing quality and aligned to the Human Genome Build 37 (hg19) using BWA (Li and Durbin, 2009).

The peaks were called using MACS (Zhang et al., 2008) for SMAD3, and SICER (Zang et al., 2009) for all other factors profiled. Only peaks common in both biological replicates were kept.

Read counting in specific genomic features was performed with the R package Rsubread (Liao et al., 2013).

Motif analyses were performed using MEME (Machanick and Bailey, 2011) and HOMER (Heinz et al., 2010). HOMER version 4.7 was run to find the distribution of the motif CCAGACA around MDA-MB-231 unique SMAD peaks, HCC-1954 unique SMAD peaks and SMAD peaks common to both cell lines. The command annotate peaks.pl was run with a bin size of 25 and a maximum distance of +/- 2500 bases around the peak centre.

DNA Methylation Profiling by MBD-sequencing

DNA methylation profiles were obtained from untreated, 7 days old MDA-MB-231 and HCC-1954 mammospheres. Methylated DNA was enriched using recombinant methyl binding domain (MBD2b/MBD3L1) protein complex as part of MethylCollector Ultra kit (Active Motif), following manufacturer's recommendations. Briefly, 1 μg of sonicated DNA was used in each MBD pulldown reaction, with high-salt buffer (AM7 buffer) in order to increase the stringency of binding conditions. Nickel-coated magnetic beads were used to precipitate His-tagged MBD complex bound to methylated DNA fragments. This pulldown reaction was incubated for 2 hours at 4°C on a turning wheel. This was followed by precipitation of beads on a magnetic stand and 4 rounds of washing with AM7 buffer. After the final wash, all residual AM7 buffer was removed. DNA was eluted from the beads and MBD complex in elution buffer containing proteinase K, for 45 min at 50°C in a thermomixer, with intermittent vortexing. DNA was further purified by phenol-chloroform extraction and ethanol precipitation. Libraries for Illumina sequencing were prepared with TruSeq LT kit (Illumina). Size selection of the DNA libraries was performed on 2% TAE agarose gels, selecting for fragments in the size range 200-600 bp. Sequencing was performed on HiSeq 2000 (Illumina), with 40 bp single end reads. Multiplexed pools of 12 samples were sequenced on a single lane to yield roughly 16 x 10^6 reads per sample.

MBD-seq Data Analysis

Briefly, bases were called and the general quality of the sequencing run was assessed by the FastQC pipeline (Andrews, 2010). The reads were then filtered based on sequencing quality and aligned to the Human Genome Build 37 (hg19) using Burrows-Wheeler Aligner (BWA) (Li and Durbin, 2009). Post-alignment, Bi-asymmetric-Laplace model (BALM) was used to call methylation peaks (Lan et al., 2011), and (MeD)IP-seq data analysis (MEDIPS) (Lienhard et al., 2014) was used for quantitative analysis, whereby the data was normalised to the CG content. Reads per kilobase per million reads (rpkm) were also calculated. Genomic regions of interest were defined based on published annotations of genomic elements (Wang et al., 2010), promoter annotation was obtained from the validated list in (Weber et al., 2007) and lifting over to hg19 was performed as previously described (Hinrichs et al., 2006).

Integration of ChIP-seq, MBD-seq and Gene Expression Data

The integration of binding sites and differentially expressed genes was performed using two different approaches. The first approach (used for Figures 1 and 2) was to simply find if a SMAD3 peak overlapped with the gene position. The gene positions were obtained from The UCSC Genome Browser Database (assembly GRCh37) and 1,500 bases upstream of the transcription start sites were added. Second approach (used for Figures 3 and S3E) was to test for differential binding between the two cell lines, in the aforementioned gene-unit spanning 1500 bases upstream of the transcription start sites to gene ends, except for DNA Methylation, where a window of -/+ 1500 bases around the TSS was taken. This was conducted using edgeR (Robinson et al., 2010) and setting the following thresholds: FDR < 0.1, absolute log Fold Change >= 0.5 and log Count per Million reads >=4.

For the analysis of distal SMAD3-bound elements, two approaches were used. In the first one (Figures S2A and S2B), peaks were annotated to a gene if they fell within a window spanning 20 kb from gene boundaries. In the second approach (Figures S2C, S2D, S2E and S2F), each SMAD3 peak was annotated to the nearest gene. The rest of the analysis was performed as for the gene-proximal SMAD3 peaks.

Calculating Enrichment of SMAD3 Binding Modes on TGFβ-dependent Genes

Each gene in the genome was annotated into one of the four defined SMAD3 binding modes, as well as into one of the four gene groups (three TGFβ-dependent gene sets: MDA-unique, HCC-unique and shared, and one TGFβ-independent gene set, containing all the other genes in the genome). Annotations were then compared to derive frequencies of each binding mode within each of the gene groups. Enrichment scores were calculated by normalising mode frequencies from TGFβ-dependent gene sets over frequencies in the TGFβ-independent gene set.

Integration of Differential Binding and Gene Set Enrichment Analyses

Enrichment of differentially bound genes in the lists of differentially expressed genes was done considering all the genes annotated in the Illumina array as universe, and the list of uniquely differentially expressed genes between TGFβ and Control in each cell line as the signature to test. Enrichment was computed using the GOSeq package (Young et al., 2010) to take into account biases due to different gene lengths.

Analysis of Gene Expression Data from Primary Tissues

Classification of Claudin^{low} samples was done following the classifier in Prat et al., 2010. A linear model was fitted to test if expression levels were different amongst groups. If the number of comparisons was larger than two, simultaneous testing was used following the R package multcomp (Hothorn et al., 2008).

Kaplan-Meier estimates and log-rank tests were computed using the R survival package (Therneau, 2014).

Supplemental References

Andrews, S. (2010). FastQC: A quality control tool for high throughput sequence data.

Barbosa-Morais, N.L., Dunning, M.J., Samarajiwa, S.A., Darot, J.F., Ritchie, M.E., Lynch, A.G., and Tavare, S. (2010). A re-annotation pipeline for Illumina BeadArrays: improving the interpretation of gene expression data. Nucleic Acids Res *38*, e17.

Cairns, J.M., Dunning, M.J., Ritchie, M.E., Russell, R., and Lynch, A.G. (2008). BASH: a tool for managing BeadArray spatial artefacts. Bioinformatics *24*, 2921-2922.

Dunning, M.J., Smith, M.L., Ritchie, M.E., and Tavare, S. (2007). beadarray: R classes and methods for Illumina bead-based data. Bioinformatics *23*, 2183-2184.

Heinz, S., Benner, C., Spann, N., Bertolino, E., Lin, Y.C., Laslo, P., Cheng, J.X., Murre, C., Singh, H., and Glass, C.K. (2010). Simple combinations of lineage-determining transcription factors prime cis-regulatory elements required for macrophage and B cell identities. Mol Cell *38*, 576-589.

Hinrichs, A.S., Karolchik, D., Baertsch, R., Barber, G.P., Bejerano, G., Clawson, H., Diekhans, M., Furey, T.S., Harte, R.A., Hsu, F.*, et al.* (2006). The UCSC Genome Browser Database: update 2006. Nucleic Acids Res *34*, D590-598.

Hothorn, T., Bretz, F., and Westfall, P. (2008). Simultaneous inference in general parametric models. Biom J *50*, 346-363.

Krueger, F., and Andrews, S.R. (2011). Bismark: a flexible aligner and methylation caller for Bisulfite-Seq applications. Bioinformatics *27*, 1571-1572.

Kundaje, A., Meuleman, W., Ernst, J., Bilenky, M., Yen, A., Heravi-Moussavi, A., Kheradpour, P., Zhang, Z., Wang, J., Ziller, M.J.*, et al.* (2015). Integrative analysis of 111 reference human epigenomes. Nature *518*, 317-330.

Lan, X., Adams, C., Landers, M., Dudas, M., Krissinger, D., Marnellos, G., Bonneville, R., Xu, M., Wang, J., Huang, T.H.*, et al.* (2011). High resolution detection and analysis of CpG dinucleotides methylation using MBD-Seq technology. PLoS One *6*, e22226.

Li, H., and Durbin, R. (2009). Fast and accurate short read alignment with Burrows-Wheeler transform. Bioinformatics *25*, 1754-1760.

Liao, Y., Smyth, G.K., and Shi, W. (2013). The Subread aligner: fast, accurate and scalable read mapping by seed-and-vote. Nucleic Acids Res *41*, e108.

Lienhard, M., Grimm, C., Morkel, M., Herwig, R., and Chavez, L. (2014). MEDIPS: genome-wide differential coverage analysis of sequencing data derived from DNA enrichment experiments. Bioinformatics *30*, 284-286.

Machanick, P., and Bailey, T.L. (2011). MEME-ChIP: motif analysis of large DNA datasets. Bioinformatics *27*, 1696-1697.

Prat, A., Parker, J.S., Karginova, O., Fan, C., Livasy, C., Herschkowitz, J.I., He, X., and Perou, C.M. (2010). Phenotypic and molecular characterization of the claudin-low intrinsic subtype of breast cancer. Breast Cancer Res *12*, R68.

Robinson, M.D., McCarthy, D.J., and Smyth, G.K. (2010). edgeR: a Bioconductor package for differential expression analysis of digital gene expression data. Bioinformatics *26*, 139-140.

Smyth, G.K. (2005). Limma: linear models for microarray data. In Bioinformatics and computational biology solutions using R and Bioconductor, V.C. R. Gentleman, S. Dudoit, R. Irizarry, W. Huber, ed. (New York, Springer Science+Business Media), pp. 397-420.

Therneau, T. (2014). A Package for Survival Analysis in S.

Wang, K., Li, M., and Hakonarson, H. (2010). ANNOVAR: functional annotation of genetic variants from high-throughput sequencing data. Nucleic Acids Res *38*, e164.

Weber, M., Hellmann, I., Stadler, M.B., Ramos, L., Paabo, S., Rebhan, M., and Schubeler, D. (2007). Distribution, silencing potential and evolutionary impact of promoter DNA methylation in the human genome. Nat Genet *39*, 457-466.

Young, M.D., Wakefield, M.J., Smyth, G.K., and Oshlack, A. (2010). Gene ontology analysis for RNA-seq: accounting for selection bias. Genome Biol *11*, R14.

Zang, C., Schones, D.E., Zeng, C., Cui, K., Zhao, K., and Peng, W. (2009). A clustering approach for identification of enriched domains from histone modification ChIP-Seq data. Bioinformatics *25*, 1952-1958.

Zhang, Y., Liu, T., Meyer, C.A., Eeckhoute, J., Johnson, D.S., Bernstein, B.E., Nusbaum, C., Myers, R.M., Brown, M., Li, W., *et al.* (2008). Model-based analysis of ChIP-Seq (MACS). Genome Biol *9*, R137.
